# Lactic acid photosynthesis via C–C cross-coupling over atomically dispersed Ba

**DOI:** 10.1038/s41467-026-73727-4

**Published:** 2026-05-29

**Authors:** Wei Wang, Yonghua Tang, ZhuiZhui Su, Huanmin Liu, You-Nian Liu, Dingguo Tang, Peng Zhou

**Affiliations:** 1 School of Environment and Energy, Peking University Shenzhen Graduate School, Shenzhen, China; 2 School of Physics and Optoelectronics, Xiangtan University, Xiangtan, China; 3 College of Chemistry and Chemical Engineering, Central South University, Changsha, China; 4 Key Laboratory of Catalysis and Energy Materials Chemistry of Ministry of Education, School of Chemistry and Materials Science, South-Central Minzu University, Wuhan, China; 5 Eco-environment and Resource Efficiency Research Laboratory, School of Environment and Energy, Peking University Shenzhen Graduate School, Shenzhen, China; 6 Guangdong Provincial Key Lab of Nano-Micro Material Research, Peking University Shenzhen Graduate School, Shenzhen, China

**Keywords:** Photocatalysis, Catalyst synthesis

## Abstract

Photocatalytic carbon–carbon coupling offers a route for converting plastic-derived chemicals into valuable multi-carbon products, yet uncontrolled coupling of intermediate limits efficiency and selectivity. Here, we report atomically dispersed barium species on titanium dioxide that act as frustrated Lewis pairs, enabling efficient and selective generation intermediates during the co-oxidation of ethylene glycol and methanol toward lactic acid. The barium species create interstitial states near the Fermi level, enhancing charge separation and surface redox activity. Meanwhile, barium sites promote the formation of transient •CHOHCH_2_OH radicals from ethylene glycol, while lattice O generates stable *CH_3_ intermediates from methanol. Their directed cross-coupling yields lactic acid at rate of 2.04 ± 0.08 mmol g^–1^ h^–1^ with 81.8 ± 2.4 % carbon selectivity, alongside H_2_ evolution at 5.85 ± 0.23 mmol g^–1^ h^–1^. In this work, we show an alkaline-earth frustrated Lewis pairs for selective solar-driven cross-coupling reaction.

## Abstract (web summary)

Atomically dispersed barium species on titanium dioxide function as frustrated Lewis pairs, enabling the efficient and selective formation of key intermediates during the cross-coupling of ethylene glycol and methanol toward lactic acid.

## Introduction

Global plastic consumer goods are rising rapidly due to enormous demand, but inadequate waste management results in over 100 million metric tons of plastic waste being discarded annually, causing significant harm to ecosystems^1–3^. A fact, plastics like polyethylene terephthalate (PET) constitute valuable resources, which can be degraded into high-quality monomer units, including ethylene glycol and terephthalic acid, through chemical or biological recycling^4,5^. Photo- and electro-chemical strategies enable the further conversion of ethylene glycol into valuable commodity chemicals, such as C_1_ (e.g., formates) and C_2_ products (e.g., glyoxal, glycolaldehyde, glycolic acid, and oxalic acid) products^6–8^. However, the synthesis of C_3+_ compounds via C–C bond formation remains underdeveloped.

**Fig. 1:**
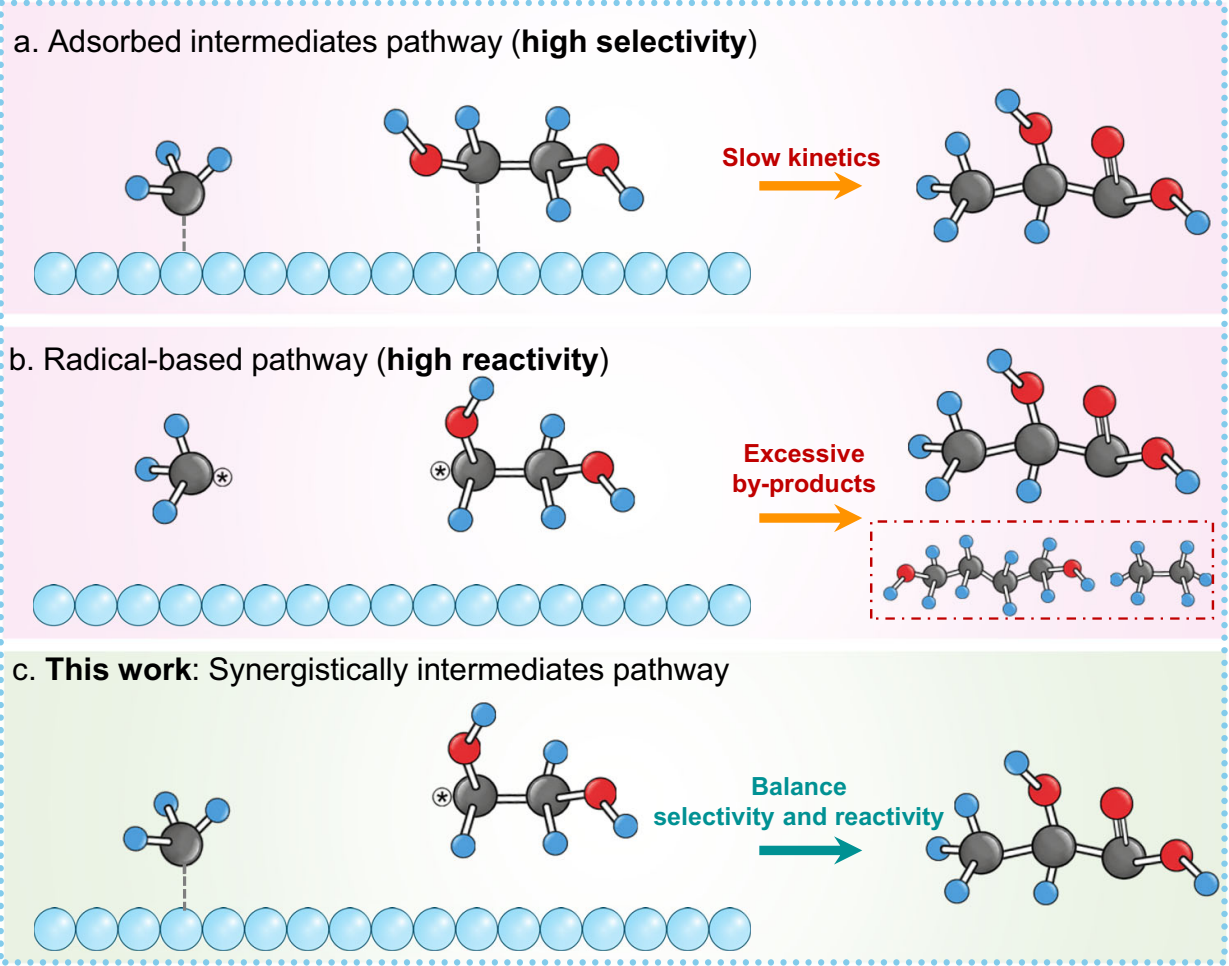
Schematic diagram. Illustration for the coupling of **a** stable-stable intermediates (adsorbed intermediates pathway), **b** transient-transient intermediates (radical-based pathway), and **c** stable-transient intermediates (synergistically intermediates pathway) for the synthesis of C_3_ compounds from ethylene glycol. The light blue balls, blue balls, dark gray balls, and red balls represent surface atoms of catalysts, H, C, and O, respectively; the asterisk and dash line represent radical and adsorbed molecules, respectively.

Recent efforts have attempted to convert ethylene glycol and methanol into valorized C_3_ chemicals, such as lactic acid^9,10^. However, electrochemical routes suffer from an intrinsic trade-off, i.e., low applied biases fail to activate inert C–H and C–O bonds, whereas high biases lead to excessive oxidation and significant selectivity losses. Heterogeneous photocatalysis offers a promising, milder alternative that enables bond activation and molecular coupling under ambient conditions^11–13^. Nevertheless, the success of such photocatalytic C–C coupling reactions strongly depends on the selective generation and regulation of key intermediates. Productive C–C coupling typically proceeds through either adsorbed alkyl species (*R) or transient radical species (•R)^14–16^. Coupling between two surface-bound intermediates is kinetically slow due to their limited reactivity (Fig. 1a)^17,18^, while coupling between two radicals is difficult to control because of their short lifetimes and the tendency for uncontrolled radical-radical recombination (Fig. 1b)^19,20^. These competing pathways often result in low coupling efficiency and poor product selectivity.

Therefore, the central scientific challenge lies in establishing a catalytic system that can simultaneously generate (i) a table surface-anchored intermediate with moderated reactivity and (ii) a transient radical species with sufficient activation energy, while ensuring their rapid and selective cross-coupling (Fig. 1c). From a structural standpoint, the conversion of methanol and ethylene glycol inherently requires the cleavage of both C–H and C–O bonds. A catalyst featuring two distinct and cooperatively functioning active sites would, in principle, enable differentiated activation of these bonds. Frustrated Lewis pairs (FLPs), consisting of spatially separated Lewis acidic and basic sites, provide an enticing conceptual framework for enabling such dual-site chemistry^21,22^. However, achieving the concurrent activation of C–H bonds in ethylene glycol and selective C–O bond cleavage in methanol via FLP-like motifs within an oxide photocatalyst remains a major unmet goal^23,24^. Previous studies suggest that alkaline-earth metals can enrich the Lewis basicity of metal-oxide surfaces, stabilize photo-generated charge carriers^25,26^, and enhance alkyl radical generation^27,28^, indicating their potential to deliver the desired dual-site environment.

In this work, we develop a photocatalytic strategy for upgrading methanol and PET-derived ethylene glycol into lactic acid through radical cross-coupling, enabled by alkaline-earth-metal-modified TiO_2_. Another PET-derived monomer unit, i.e., terephthalic acid, is first assembled into a MIL-125(Ti) framework, which allows confined incorporation of alkaline earth ions; subsequent annealing yields atomically dispersed alkaline-earth metal-modified TiO_2_ (M:TiO_2_) with FLP sites. These sites promote methanol deoxygenation to form surface-bound *CH_3_ intermediates and simultaneously regulate ethylene glycol adsorption through double M–O coordination, enabling controlled generation of •CHOHCH_2_OH radicals. Among the alkaline-earth series, Ba provides the most effective dual-site (FLPs) environment, delivering the highest rate of radical-surface coupling. Thus, Ba:TiO_2_ achieves a lactic acid production rate of 2.04 ± 0.08 mmol g^−1^ h^−1^ with 81.8 ± 2.4% carbon selectivity, accompanied by anaerobic H_2_ evolution at 5.85 ± 0.23 mmol g^−1^ h^−1^. Spectroscopic and theoretical analyses show that alkaline-earth incorporation elevates the band structure and lowers the thermal activation barrier for detrapping photogenerated carriers, thereby enhancing charge-carrier separation, with Ba exhibiting the strongest effect. In situ measurements further confirm that the engineered pairing of stabilized *CH_3_ species with transient radicals underlies the high selectivity.

## Results

### Reaction energy barriers of alkaline earth metal modified TiO_2_ (M:TiO_2_)

**Fig. 2:**
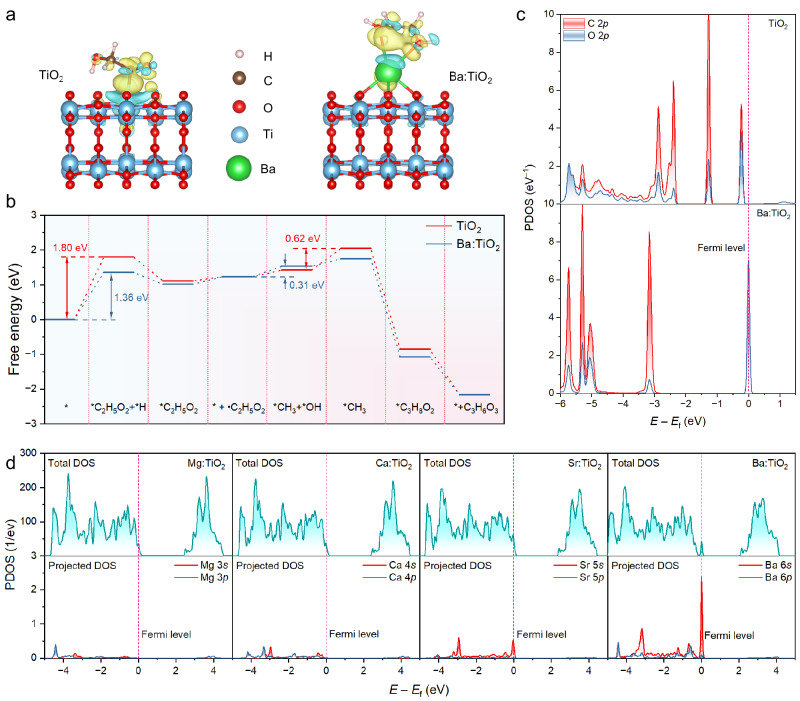
Investigation of thermochemical reaction energetics. **a** Charge density difference mapping between *CHOHCH_2_OH intermediate and catalyst surface on TiO_2_ (left) and Ba:TiO_2_ (right) generated by VESTA visualization software^63^, the skyblue and yellow isosurfaces stand for the negative and positive charges, respectively; the isosurface of charge density is set to 0.002 e Å^−3^; the pink balls, brown balls, red balls, light blue balls and green balls represent H, C, O, Ti, and Ba atom, respectively. **b** Free energy profile of lactic acid (LA) production on TiO_2_ and Ba:TiO_2_ at pH = 7 and *U* = 0 V vs. SHE. **c** Projected density of states (projected DOS) of C and O 2*p* states of *CHOHCH_2_OH intermediates, the dashed red line stands for the Fermi level. **d** Total density of states (total DOS) and PDOS of Mg-, Ca-, Sr-, and Ba-modified bulk TiO_2_, the dashed red line indicates the Fermi level.

Here, the reaction energy barriers of the cross-coupling reaction between methanol (CH_3_OH) and ethylene glycol (EG, CH_2_OHC_2_HOH) on M:TiO_2_ for the production of D,L-lactic acid (LA) were investigated by using density functional theory (DFT) calculations. We used an anatase structure for TiO_2_ and M:TiO_2_, because the anatase type TiO_2_ has superior catalytic activity among the different phases^29,30^. Additionally, the thermodynamically stable anatase (1 0 1) facets were considered in the simulation. The alkaline-earth metals tend to coordinate with five O atoms in anatase (1 0 1) facets. The structures of intermediates in the reaction pathway are shown in Fig. 2a and Supplementary Figs. 1 and 2. When conducting electronic performance calculations, the atomic coordinates of all the optimized computational models are contained in the Supplementary Data 1–7.

Free energy diagrams for pristine TiO_2_ and Ba:TiO_2_ (an example) are presented in Fig. 2b. Firstly, CH_2_OHC_2_HOH is oxidatively dissociated on the metal–O pair (Ti–O or Ba–O) to form *CHOHCH_2_OH intermediate. Meanwhile, a bridged hydroxy is produced, which is deprotonated in the following step. Then, the *CHOHCH_2_OH intermediate is desorbed to form •CHOHCH_2_OH free radical (transient intermediates). To produce the methyl-containing LA molecule, CH_3_OH is oxidatively dissociated on the M–O pair to form *CH_3_ (stable intermediates) and *OH intermediates by cleaving the C–O bond, in which the *OH intermediate is reduced into H_2_O. Meanwhile, *CH_3_ is left on bridged oxygen to bond with •CHOHCH_2_OH transient intermediates through C–C cross-coupling reaction. Thus, a *CH_3_CHOHCH_2_OH intermediate is formed. Under strongly alkaline conditions, the formed *CH_3_CHOHCH_2_OH intermediate can be successively base-catalyzed and photo-catalytically oxidized into LA^31^. It should be noted that CH_3_OH and EG would be directly converted into formic acid and glycolic acid when the base catalysis is dominant in the first deprotonation of the hydroxy group in alcohols. For LA production, the potential rate-determining step on TiO_2_ and Ba:TiO_2_ is suggested to be the formation of *CHOHCH_2_OH. According to the results obtained (Fig. 2b), the theoretical LA activities differ significantly between the TiO_2_ and M:TiO_2_. In the presence of atomically dispersed Ba, the energy barrier of oxidative dissociation of EG is reduced from 1.80 to 1.36 eV, compared with pristine TiO_2_. The existence of Mg, Ca, and Sr shows a lower energy barrier of oxidative dissociation of EG than TiO_2_, which suggests the introduction of alkaline earth modification to significantly enhance LA activity (Supplementary Fig. 3). Additionally, to evaluate the competing production of some byproducts from non-C–C coupling reaction, the free energy profiles of generating formic acid from CH_3_OH and glycolic acid from EG were also calculated on Ba:TiO_2_ (Supplementary Figs. 4 and 5). The results obtained show that the generation processes of formic acid and glycolic acid involve two high-barrier reaction steps with reaction energies exceeding 1 eV. However, apart from the rate-determining step, other reaction steps in the generation process of LA over Ba:TiO_2_ exhibit reaction energies lower than 0.3 eV, suggesting that the generation of LA is prior to those of formic acid and glycolic acid over Ba:TiO_2_. Therefore, the addition of Ba can not only theoretically improve the LA generation rate of LA but also endow TiO_2_ with high selectivity for LA over formic acid or glycolic acid.

Calculated charge density difference maps indicate that the promotion effect induced by alkaline-earth atoms arises from the formation of double metal–O bonds. For instance, Ba–O bonds (as shown in Fig. 2a) from between the *CHOHCH_2_OH intermediate and the Ba site, which significantly reduces the energy required for EG dissociation. Projected density of states (PDOS) plots of *CHOHCH_2_OH intermediate further corroborate this finding (Fig. 2c). The C 2*p* and O 2*p* states of *CHOHCH_2_OH on Ba:TiO_2_ exhibit a more localized distribution, indicating a more stable chemical interaction between the intermediate and Ba:TiO_2_. Furthermore, the edge of 2*p* states of the bridged oxygen atom adjacent to the alkaline-earth atom are negative, which favors the formation of the O–H bond during EG dissociation (Supplementary Fig. 6). The influence of alkaline-earth metals within the TiO_2_ lattice was also examined. Based on the structural characteristic of bulk TiO_2_, Ba species tend to substitute for the Ti atom, which are coordinated with six O atoms. Band structure analysis reveals that only the Ba species generates strong interstitial states slightly above the valence band (Fig. 2d). The Fermi level is positioned within the Ba 6*p* states, suggesting that the Ba site can effectively capture electrons from the valence band to promote charge separation^32,33^. The calculation results provide qualitative insights into the reaction pathway and the potential role of alkaline-earth metals sites. However, simplified models cannot fully capture catalytic structural complexity and dynamics; experimental studies are necessary to validate the proposed mechanisms and clarify their effects on key intermediates in EG–CH_3_OH cross-coupling.

### Preparation and characterization of alkaline-earth metal-modified TiO_2_ (M:TiO_2_)

**Fig. 3:**
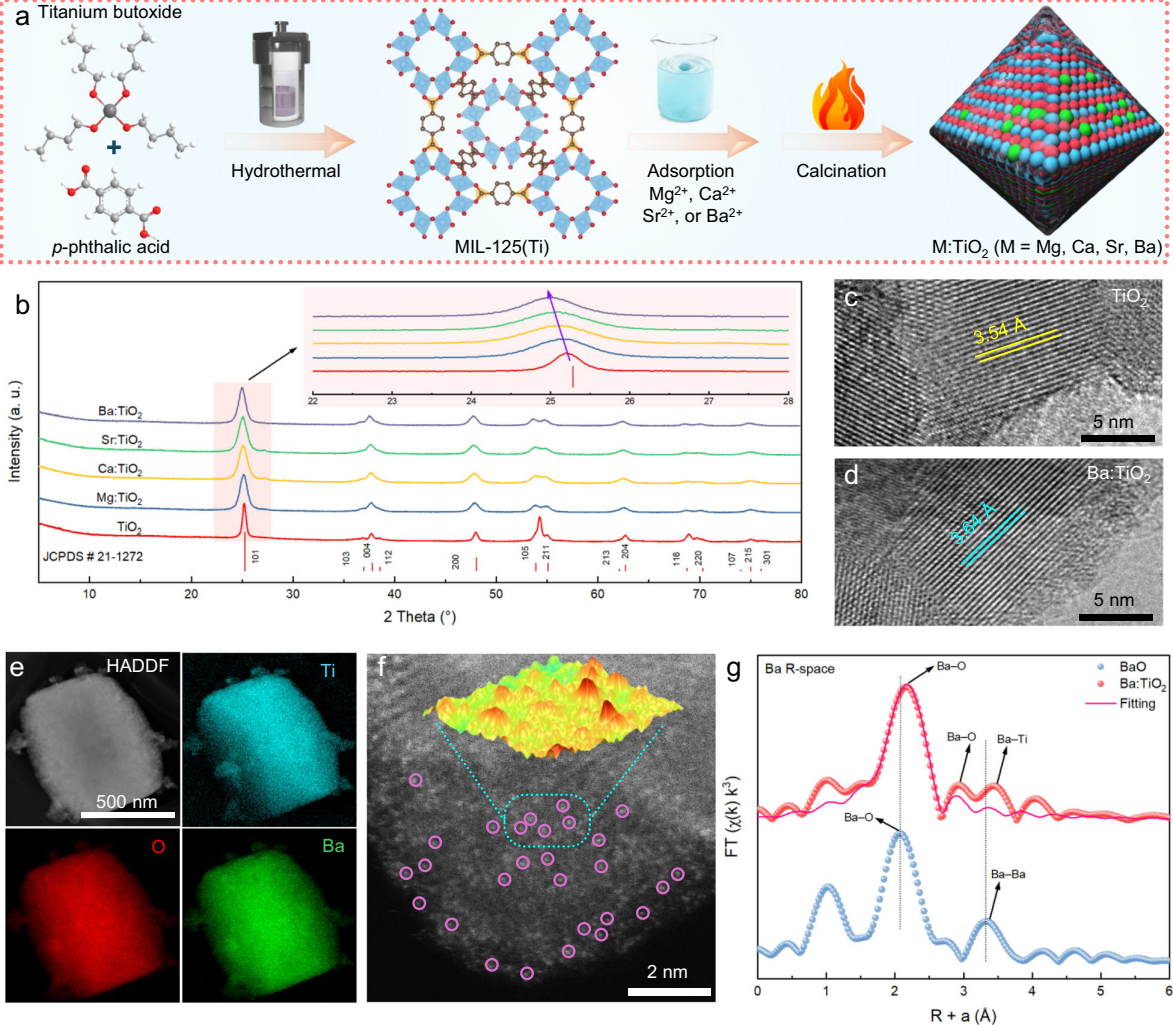
Preparation and structural characterization of TiO_2_ and M:TiO_2_ photocatalysts. **a** Schematic of the process leading to the fabrication of TiO_2_ samples. **b** PXRD spectra of TiO_2_ and M:TiO_2_, inset: enlarged view of PXRD spectra from 22° to 28°. HRTEM lattice images of **c** TiO_2_ and **d** Ba:TiO_2_ with crystalline TiO_2_ (1 0 1). **e** HAADF-TEM of Ba:TiO_2_ and the corresponding element mapping. **f** AC-STEM image of Ba:TiO_2_, inset: 3D color map of the cyan-outline region, the protruding tip being Ba single atoms. **g** Fourier transform of k^3^-weighted EXAFS spectra at Ba K-edge of Ba:TiO_2_ and BaO.

To evaluate the theoretical prediction of high LA activity and selectivity of M:TiO_2_ photocatalysts, we prepared them using atomic-level catalyst design strategy, with porous MIL-125(Ti) as a substrate and an ion-coordination method (Fig. 3a)^34^. First, we dissolved terephthalic acid and titanium butoxide in a mixture solvent of N,N-dimethylacetamide and ethanol to form MIL-125(Ti) via a hydrothermal process. Alkaline earth cations were then added to the dispersion of MIL-125(Ti) in alcohol to form M-MIL-125(Ti). Finally, M:TiO_2_ powder was obtained by calcination under an oxygen atmosphere. From inductively coupled plasma-atomic emission spectrometry (ICP-AES) analysis, we determined the alkaline earth addition amount of M:TiO_2_ to be about 0.92 wt% vs. TiO_2_ (Supplementary Table 1). Moreover, because it was annealed at high temperature in an oxygen atmosphere (Supplementary Fig. 7), the M:TiO_2_ photocatalyst exhibited no significant oxygen vacancies and residual carbon (Supplementary Figs. 8 and 9).

Powder X-ray diffraction (PXRD) analysis showed that the pristine TiO_2_ and M:TiO_2_ are the typical crystallographic structure of anatase (Fig. 3b)^35,36^. Notably, with the incorporation of alkaline earth metals and the increase in their atomic radii, the characteristic PXRD peak at (1 0 1) shifts toward lower 2 theta angles (from 25.2° to 25.0°). This indicates that the alkaline earth metals enter the TiO_2_ lattice, causing significant lattice expansion^37^. This result is also supported by high-resolution transmission electron microscopy images, where the lattice fringes of 0.354 and 0.364 nm are attributed to the (1 0 1) crystal planes of TiO_2_ (Fig. 3c) and Ba:TiO_2_ (Fig. 3d), respectively.

Furthermore, the X-ray photoelectron spectroscopy (XPS) depth profiling and the focused ion beam-scanning electron microscopy (FIB-SEM) cross-sectional elemental distribution analysis revealed that the Ba atom exhibits uniform distribution within Ba:TiO_2_ (Supplementary Figs. 10 and 11). Meanwhile, the high-angle annular dark field image (HADDF-TEM) and EDS mapping of Ba:TiO_2_ show the uniform distribution of Ba, O, and Ti elements on the disk-like nanoparticles (Fig. 3e). These results demonstrate that in the M:TiO_2_ prepared by our method, the alkaline earth metals are not merely a surface-adsorbed phase but are deeply incorporated into the interior of the nanoparticles. The aberration-corrected scanning transmission electron microscopy (AC-STEM) reveals that disc-like Ba:TiO_2_ is nanoporous, and bright dots on the TiO_2_ matrix are assigned to Ba single atoms (Fig. 3f and Supplementary Fig. 12). These nanopores with around 1–3 nm originated from the non-collapsed pore structure of MIL-125(Ti) (Supplementary Fig. 13). The coordination environment of Ba species in Ba:TiO_2_ was further investigated by Ba K-edge X-ray absorption near edge structure and extended X-ray absorption fine structure (EXAFS) analysis (Supplementary Figs. 14 and 15). The k^3^-weighted Fourier-transformed EXAFS spectra show that Ba:TiO_2_ only has a dominant peak at 2.18 Å, assigned to Ba–O bond (Fig. 3g). Notably, the peak at 2.93 Å can be attributed to the scattering signals of Ba–O bonds with different bond lengths. Meanwhile, the R-space spectra of Ba:TiO_2_ exhibit only a weaker feature assignable to Ba–Ti scattering at 3.45 Å, while the second-shell Ba–Ba scattering contribution of BaO is clearly observed at 3.32 Å. The overall suppression of the Ba–Ba coordination signal indicates that Ba species predominantly exist as atomically dispersed single atoms within the TiO_2_ matrix. In particular, the EXAFS fitting results further confirm the atomically dispersed nature of Ba, coordinated to 5.88 O atoms. According to the above models, the surface and bulk Ba species are coordinated to five and six O atoms, respectively. The theoretical average coordination number of Ba in the whole TiO_2_ is between five and six, consistent with the fitting result of EXAFS.

Meanwhile, as shown in Supplementary Fig. 16, the Raman spectra further confirm that all TiO_2_ samples exhibit an anatase structure. Similarly, the *E*_g_ band (144 cm^−1^) exhibits a greater blue shift with increasing atomic radius of the introduced alkaline earth metal, and the maximum change occurs with Ba introduction. The existence of the Ba–O bond would lead to the local extrusion stress in the neighboring Ti–O bond, resulting in local distortion and bond-strength redistribution (Supplementary Fig. 17)^38–40^. Further, the XPS spectra of O 1*s* reveal that the signals of lattice oxygen (O^2–^) of M:TiO_2_ have a slight blue shift in comparison with that of TiO_2_ (Supplementary Fig. 18a). Meanwhile, the XPS spectra of Ti 2*p* can be deconvoluted into Ti^4+^ 2*p*_1/2_ (485.5 eV) and Ti^4+^ 2*p*_3/2_ (484.3 eV), respectively, both of which exhibit blue shifts on M:TiO_2_ relative to TiO_2_ (Supplementary Fig. 18b). Similarly, these shifts increase with the atomic radius of alkaline earth metals. This evidence indicates that alkaline-earth-metal inject a significant number of electrons into the TiO_2_ conduction band, lowering the Fermi level and increasing the binding energies of the Ti 2*p* and O 1 *s*^41,42^. Alkaline-earth metals with larger atomic radii typically exhibit stronger electron-donating properties. Consequently, as the atomic radius of alkaline earth metals increases, the effect of electron injection into the conduction band becomes more pronounced (electronegativity, Mg: 1.31, Ca: 1.00, Sr: 0.95, and Ba: 0.89), leading to an enhanced blue shift in the O 1*s* and Ti 2*p* binding energies. Such alterations may enhance the surface redox properties and photo-generated carriers separation characteristics of TiO_2_.

### Evaluation of photochemical C–C cross-coupling activity

**Fig. 4:**
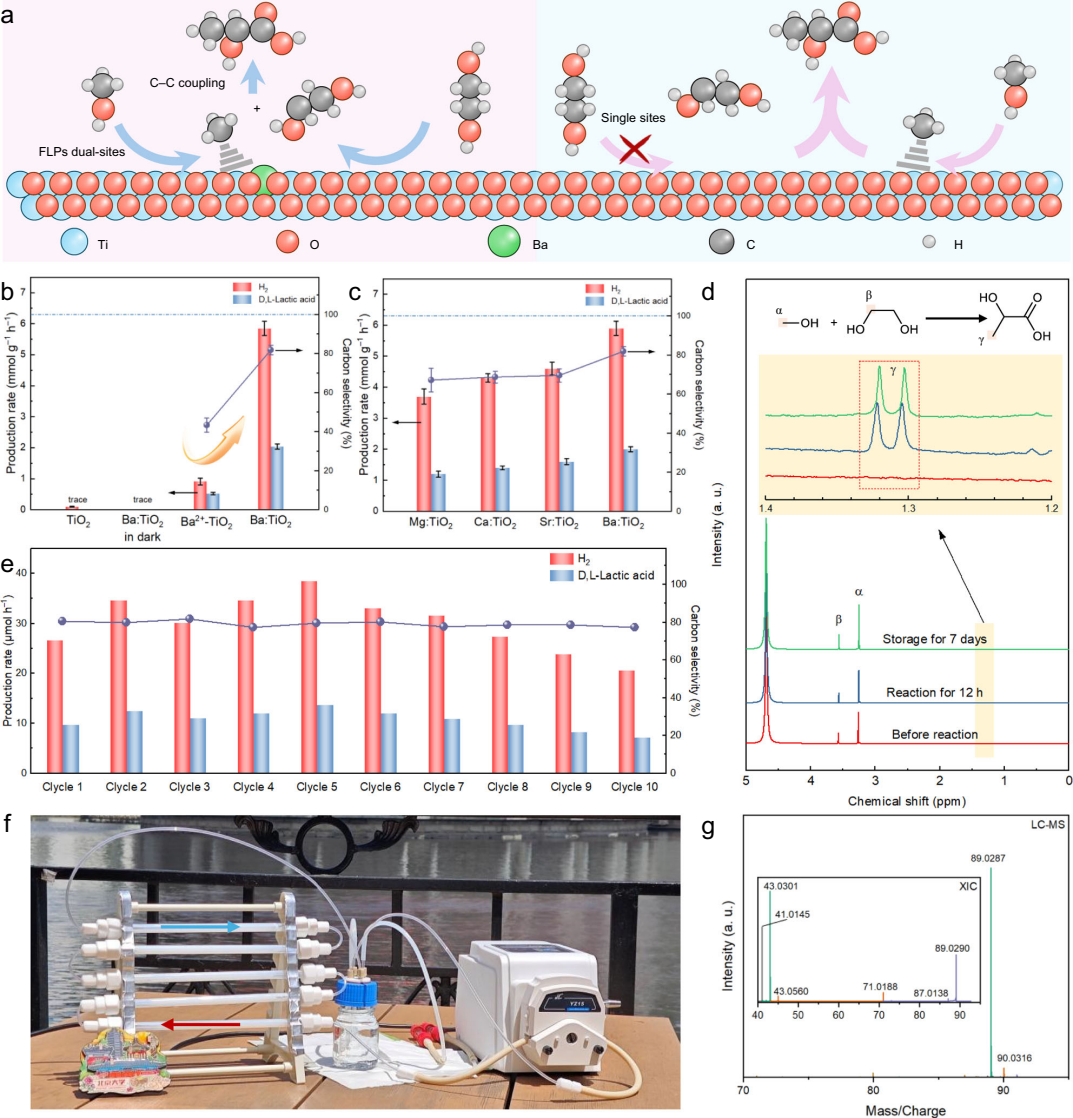
Photocatalytic synthesis of LA from MeOH and EG. **a** Scheme for photochemical C–C cross-coupling reaction of CH_3_OH and EG to LA on frustrated Lewis acid (FLPs) dual sites (M:TiO_2_) and single sites (TiO_2_), where the light blue balls, red balls, green balls, dark gray balls, and gray balls represent Ti, O, Ba, C, and H atoms, respectively. **b** LA and H_2_ production rate over different types of Ba relative samples and pristine TiO_2_, and **c** different kinds of alkaline earth metal introduction; the bar chart uses the left vertical axis, while the point plot uses the right vertical axis, as indicated by these arrows. Reaction conditions: 5 mg of photocatalysts, 20 mL of reaction mixture (50 vol% CH_3_OH, EG with molar ratio to CH_3_OH 1:5, and KOH with concentration is 4.0 M), *T* = 25 °C, 1 bar Ar, *t* = 2 h, and Xe lamp irradiation (360 mW cm^–2^), a minimum of three replicate measurements was performed for each material group to ensure reproducibility. **d** ^1^H NMR of the reaction system using Ba:TiO_2_ as a catalyst before reaction, after 12-h reaction, and after 7-day storage at room temperature following 12-h reaction, inset: enlarged view of ^1^H NMR spectra, and the α-, β-, and γ-labeled sites refer to the hydrogen atoms and correspond to the NMR signals of methanol, ethylene glycol, and lactic acid, respectively. **e** Activity and selectivity results during ten cycles of the Ba:TiO_2_ catalyst, each cycle lasting 2 h, the bar chart uses the left vertical axis, while the point plot uses the right vertical axis. **f** Photograph of the continuous-flow photoreactors. **g** LC-MS of the continuous-flow reaction system after a full-day (from 9:00 to 17:00) outdoor test (22°35′49′′ N, 113°58′22′′ E), inset: extracted ion chromatogram (XIC) of mass/charge from 40 to 100.

Above theoretical calculations predict that the introduction alkaline earth metals in TiO_2_ promotes the conversion of ethylene glycol into alkyl radicals (transient intermediates), thereby facilitating C–C cross-coupling reaction with adsorbed *CH_3_ groups to yield lactic acid (LA), as shown in Fig. 4a. Direct photocatalytic C_3_ products (LA) synthesis was attempted in an aqueous solution of CH_3_OH and EG, which was quantitatively analyzed through high-performance liquid chromatography (HPLC, Supplementary Fig. 19). The obtained activity results show that the experimental trend is consistent with the thermodynamic analysis of various M:TiO_2_ and pristine TiO_2_ samples. As shown in Fig. 4b, pristine TiO_2_ only produced a trace amount of lactic acid via HPLC analysis. However, when Ba was introduced as a cation into the reaction solution, the measurable production rates of H_2_ (0.91 ± 0.11 mmol g^−1^ h^−1^) and LA (0.52 ± 0.04 mmol g^−1^ h^−1^) were observed, with a LA carbon selectivity of 54.0 ± 3.6%. When Ba was directly incorporated into TiO_2_ photocatalysts, the production rates of H_2_ and LA dramatically increased to 5.85 ± 0.23 and 2.04 ± 0.08 mmol g^−1^ h^−1^, respectively, with a carbon selectivity of 81.8 ± 2.4% (Supplementary Table 2). The photocatalytic coupling performance of Ba:TiO_2_ was further valuated using action spectra, namely the apparent quantum yield (AQY). As shown in Supplementary Fig. 20, the AQY (for the synthesis of LA) of Ba:TiO_2_ exhibits a positive correlation with its UV–vis DRS spectrum, demonstrating an AQY of 2.81 ± 0.04% at 350 nm (5 mg of photocatalysts). Besides, both M:TiO_2_ (such as Mg:TiO_2_, Ca:TiO_2,_ and Sr:TiO_2_) demonstrated similar dramatic increases in reactivity and selectivity, though the Ba:TiO_2_ showed the highest improvement (Fig. 4c). Notably, this catalytic system was achieved without the addition of any precious metals. Our photocatalytic approach achieves direct C–C coupling under light-driven conditions, representing a complementary and sustainable strategy. The aforementioned product formation rates were obtained through optimized reaction conditions shown in the Supplementary Figs. 21–27.

It should be further clarified that, in the dark or in the absence of reactants, only trace amounts of the corresponding oxidation (LA) and reduction (H_2_) products can be detected in the Ba:TiO_2_ catalyzed system (Fig. 4b and Supplementary Fig. 28). This indicates that the C–C cross-coupling reaction between EG and CH_3_OH is a photosensitive reaction, rather than a conventional heterogeneous catalytic reaction. Further evidence is provided by liquid ^1^H nuclear magnetic resonance (NMR) testing shown in Fig. 4d. After storing a reaction mixture at room temperature (~30 °C, close to the temperature in the photocatalysis process) for 7 days, the characteristic peak area around 1.3 ppm for the methyl group of LA remained virtually unchanged upon subsequent ^1^H NMR. This also suggests the reaction is not a heterogeneous catalysis process.

The reducibility and stability are critical parameters for practical applications. Through ICP-AES analysis, the Ba content in Ba:TiO_2_ remains essentially unchanged after a 12-h reaction (0.91 wt%) compared to the initial value (0.92 wt%), indicating negligible Ba leaching and compositional variation during operation. In cycling measurement, Ba:TiO_2_ maintains steady product selectivity over 10 consecutive runs (Fig. 4e), although a gradual decrease in activity is observed. The activity loss may partially originate from catalyst mass loss during centrifugal recovery, while surface deactivation of Ba:TiO_2_ cannot be excluded. To demonstrate the integration of a photocatalytic device, we developed a tandem photocatalytic comprising a continuous-flow reactor powered by natural sunlight. The Ba:TiO_2_ powder was immobilized in the continuous-flow reactor to produce LA (Fig. 4f), thereby avoiding the mass loss during the process relative to catalyst separation from the reaction solution. Through a full day of outdoor sunlight exposure (from 9:00 to 17:00) (Supplementary Fig. 29), approximately 270 µmol of LA were generated (Fig. 4g and Supplementary Fig. 30).

### Band structure and charge transfer behaviors

The contribution of alkaline earth metals to the generation of photo-generated electrons and holes in TiO_2_ was investigated via band structure analysis. As shown in Supplementary Fig. 31, the light absorption ability of the as-prepared TiO_2_ samples was determined by UV–vis diffuse reflectance spectra (UV–vis DRS). The incorporation of alkaline earth metals enhances the Ti–O bond strength. As a consequence, the absorption edge of M:TiO_2_ shifts to approximately 380 nm, exhibiting a slight blue shift compared to pristine TiO_2_ (ca. 410 nm). Notably, the incorporation of Ba with a much larger atomic radius than Ti induces lattice distortion and local electrostatic potential redistribution, which collectively contribute to a modest blue shift of the optical absorption. The band gap energies of these TiO_2_ samples were estimated to be about 2.94 (TiO_2_), 3.03 (Mg:TiO_2_), 3.09 (Ca:TiO_2_), 3.05 (Sr:TiO_2_), and 3.05 eV (Ba:TiO_2_), respectively, according to the Tauc plots (Supplementary Fig. 32). To understand the valence band (VB) of TiO_2_ samples, the VB-XPS spectra of each counterpart were acquired^43^, as shown in Supplementary Fig. 33. The determined VB potential for both TiO_2_ and M:TiO_2_ samples was located at about 2.65 (TiO_2_), 2.61 (Mg:TiO_2_), 2.55 (Ca:TiO_2_), 2.49 (Sr:TiO_2_), and 2.46 V vs. RHE (Ba:TiO_2_). It should be noted that the measurement error is eliminated by a formula in the Supplementary Eq. 1. Combining with the obtained band gaps, the conduction band potentials of TiO_2_ and M:TiO_2_ can be determined to be −0.29 (TiO_2_), −0.42 (Mg:TiO_2_), −0.54 (Ca:TiO_2_), −0.56 (Sr:TiO_2_), and −0.59 V vs. RHE (Ba:TiO_2_), with the band structure comparison shown in Supplementary Fig. 34. Apparently, the introduction of alkaline earth metals did not reduce the band gap of TiO_2_. Instead, the entire band structure of TiO_2_ was shifted upward, thereby improving its thermodynamic activity for hydrogen evolution reactions.

**Fig. 5:**
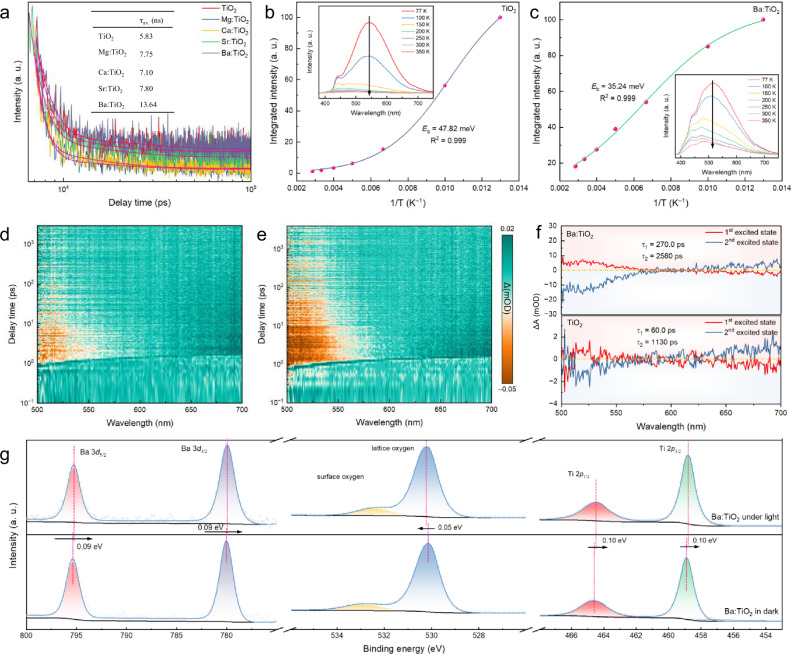
Separation and transfer behaviors of photo-generated carriers. **a** Transient PL spectra of TiO_2_ and M:TiO_2_ samples (λ_ex_: 350 nm, λ_monitoring_: 450 nm), inset: average fluorescence lifetime (*τ*_av._) of various samples. Fitting results of temperature-dependent PL spectra for **b** TiO_2_ and **c** Ba:TiO_2_, the *E*_b_ is the thermal activation energy, λ_ex_: 350 nm, inset: temperature-dependent PL spectra and these arrows represent the direction of temperature increase. TAS spectra of **d** TiO_2_ and **e** Ba:TiO_2_ under 350-nm excitation, and **f** corresponding SAS spectra. **g** In situ XPS spectra of Ba:TiO_2_ under illumination for 15 min, these arrows represent the direction of peak shift.

To understand the exciton dissociation ability of TiO_2_ and M:TiO_2_ photocatalysts, the photoluminescence (PL) behaviors of the samples were carefully analyzed. The steady state PL spectra shows that the emission intensity observed from M:TiO_2_ is weaker than that of TiO_2_, and the degree of weakening increases significantly with increasing alkaline earth atomic radius, reaching its maximum in Ba:TiO_2_ (Supplementary Fig. 35). The PL quenching observed on M:TiO_2_ implying that the intrinsic radiative recombination of photo-generated electron-hole pairs has been substantially inhibited, since the strong interstitial states are formed around Fermi level (Fig. 2d). The transient PL decay profiles monitored at the steady-state emission peaks demonstrate a markedly prolonged carriers lifetime for Ba:TiO_2_, with an average lifetime (*τ*_av._) of 13.64 ns, compared with TiO_2_ (5.83 ns), Mg:TiO_2_ (7.75 ns), Ca:TiO_2_ (7.10 ns), and Sr:TiO_2_ (7.80 ns). This extended lifetime indicates enhanced charge separation and transfer efficiency in the Ba-modified sample Fig. 5a and Supplementary Table 3)^44–46^. In short, bulk-incorporated alkaline earth metals, particularly Ba, facilitate electron capture and redistribution, thereby suppressing radiative recombination and modulating the optical response.

Besides, as shown in Fig. 5b, c, investigated the thermal activation energy for charge carrier release via temperature-dependent PL spectra. As the temperature increased from 77 to 350 K, the integrated PL intensity of TiO_2_ and Ba:TiO_2_ decreased monotonically, which was mainly attributed to a thermally activated non-radiative recombination process. Given that the PL emission occurs at energies significantly below the band gap, the signal likely originates from trap states rather than direct band-edge recombination. By fitting the experimental data with the Arrhenius equation, the thermal activation energies (*E*_b_) of TiO_2_ and Ba:TiO_2_ were determined to be 47.82 and 35.24 meV, respectively. This lower activation energy in Ba:TiO_2_ indicates a reduced barrier to charge carrier release from trap states and subsequent migration, leading to less energy loss during charge migration. These results demonstrate that the introduction of an alkaline earth metal can reduce the effective thermal activation barrier, thereby facilitating the generation and transport of photogenerated carriers in TiO_2_.

Furthermore, the dynamic information on the excited state charge in TiO_2_ and M:TiO_2_ (Ba:TiO_2_) was studied using femtosecond transient absorption spectroscopy (TAS). The samples were photo-excited by 350-nm pump pulses, and the TAS was acquired with a probe pulse. Background subtraction and chirp correction were performed on the data. As shown in Fig. 5d, e, TiO_2_ and Ba:TiO_2_ exhibit instantaneous negative signals around 500 nm upon photoexcitation, which can be primarily attributed to ground-state bleaching (GSB) with possible contributions from simulated emission (SE)^47,48^. For TiO_2_, the negative feature gradually narrows and decreases in intensity over time. In contrast, Ba:TiO_2_ exhibits a progressive redshift of the negative band over time. To quantitatively analyze the carrier dynamics, global fitting was performed using a sequential kinetic model^49^, yielding the species-associated spectra shown in Fig. 5f. Both samples exhibit two dominant kinetic components within the measured time window. For TiO_2_, the long-lived component decays within 1.1 ns, indicating relatively fast carrier recombination and limited excited-state persistence. In comparison, Ba:TiO_2_ approximately 2.3-fold increase in lifetime (2.6 ns). This prolonged excited-state lifetime suggests that Ba incorporation effectively retards electron-hole recombination dynamics, thereby stabilizing photogenerated charge carriers over longer timescales. Such behavior is consistent with the enhanced photocatalytic performance observed for Ba:TiO_2_.

Furthermore, the in situ XPS results under illumination show hole accumulation on lattice oxygen and electron accumulation on Ti or Ba, suggesting that the bulk-incorporated Ba species play a critical role beyond surface chemistry (Fig. 5g). The bulk Ba species likely create a distributed network of hole-stabilizing centers throughout the TiO_2_ lattices, work in synergy with the surface FLP sites to achieve efficient bulk charge separation and directed surface delivery of reactive holes.

The rapid separation of photo-generated carriers was further validated by electrochemical impedance spectroscopy (EIS) measurements shown in Supplementary Fig. 36. The EIS and corresponding fitting results (Supplementary Table 4) show the fastest charge transfer kinetics for Ba:TiO_2_, with the order being TiO_2_ < Mg:TiO_2_ < Ca:TiO_2_ < Sr:TiO_2_ < Ba:TiO_2_. As a result, in transient photo-current tests, the order of photocurrent response for samples is TiO_2_ < Mg:TiO_2_ < Ca:TiO_2_ < Sr:TiO_2_ < Ba:TiO_2_ (Supplementary Fig. 37). Photochemical properties and electrochemical performances indicate that the alkaline earth metal introduction can improve the separation of photogenerated carriers and direct the transport of photogenerated electrons and holes to metal and lattice oxygen sites.

### Generation and coupling mechanism of the key intermediates

**Fig. 6:**
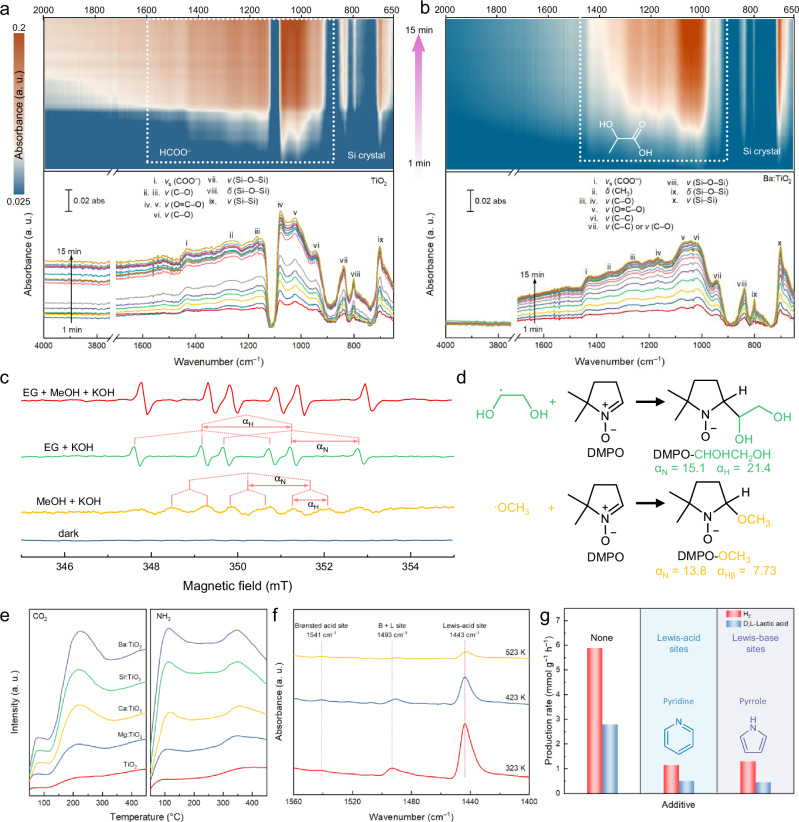
Generation and transformation mechanism of the key intermediates. Time-dependent IR signals for **a** pristine TiO_2_ and **b** Ba:TiO_2_ under our reaction conditions, the absorption signals within the white dashed box indicate the formation of specific products; these arrows represent the direction of time increases. **c** 5,5-dimethyl-1-pyrroline N-oxide (DMPO)-trapping in situ ESR experiments and **d** corresponding radical characteristic values, where EG and MeOH represent ethylene glycol and methanol, respectively. **e** CO_2_ and NH_3_ TPD profiles of pristine TiO_2_ and M:TiO_2_. **f** Py-IR for Ba:TiO_2_. **g** Photocatalytic performance of Ba:TiO_2_ without adsorption and with pyrrole or pyridine adsorption.

To identify the critical step of the co-oxidation of MeOH (CH_3_OH) and EG (CH_2_OHCH_2_OH). Variable-controlled in situ attenuated total reflection Fourier transform infrared spectroscopy (ATR-FTIR) investigations are conducted by comparing IR signals from pristine TiO_2_ and M:TiO_2_ (Ba:TiO_2_). As the co-oxidation over TiO_2_ proceeds (Fig. 6a), the gradual consumption of C–OH (1100 cm^−1^) is observed, which generates the corresponding oxidative (C–O of O=C–O at 1266, 1165, 1079, 1020, and 946 cm^−1^, and other C–O at 943 cm^−1^)^50,51^. Besides, the spectra show a broad band centered at 1430 cm^−1^ corresponding to the O=C–O asymmetric stretching mode of formate^52,53^. The bands at 840, 800, and 702 cm^−1^ detected in the uppermost spectrum are caused by the optical interference from the stretching mode of Si–O and other vibrations in the Si@TiO_2_ substrate^50^. As shown in Supplementary Fig. 38, these substrate-derived signals are also detectable during in situ ATR-FTIR testing using a neat ATR crystal (i.e., single-crystal silicon). So far, we can definitively state that the peroxidation of MeOH and EG, yielding carboxylic acid products, is represented by formic acid. Qualitative analysis by HPLC further indicates that significant amounts of formic acid, glycolic acid, and other products are generated during this process (Supplementary Fig. 39).

Compared to the spectra using TiO_2_ as a catalyst, the spectra over Ba:TiO_2_ serving as the catalyst did not immediately show the significant depletion of the C–OH bond (Fig. 6b). Instead, a slight decrease in the intensity of the C–OH-related IR absorption band was observed only after 5 min of illumination, with the intensity remaining stable at 10 min. Meanwhile, two notable differences emerged, i.e., observing the strong absorption band (1350 cm^−1^) of methyl bending vibration and the weak absorption bands for C–C bond stretching vibrations (1010 and 942 cm^−1^)^54^. Additional peaks characteristics of carboxylic acid further confirm the formation of the target product, such as COO^–^ at 1430 cm^−1^, O=C–O at 1070 cm^−1^, and C–O at 1258 and 1163 cm^−1^. Therefore, it is identified that the C–C bond can be effectively generated through the formation of a stable *CH_3_-intermediate, consistent with theoretical calculations. Furthermore, an in situ electron spin resonance (ESR) experiment is conducted to dynamically track the selective C–C cross-coupling pathways and corresponding intermediates, by applying 5,5-dimethyl-1-pyrroline N-oxide (DMPO) as the trapping agent under an Ar atmosphere. The hydroxymethyne radical (•CHOHCH_2_OH, *α*_N_ = 15.1, and *α*_H_ = 21.4) from the EG transformation was detected under our reaction conditions (Fig. 6c, d). Meanwhile, Ba:TiO_2_ generates significant hydroxyl radicals (•OH, *α*_N_ = 14.9, and *α*_H_ = 14.9) under neutral conditions, whereas almost no •OH are detectable under our reaction conditions (Supplementary Fig. 40). This indicates that the holes generated by the catalyst directly react with methanol or ethylene glycol without involving water as a transfer medium, which may reduce energy dissipation. Moreover, methyl radicals (•OCH_3_, *α*_N_ = 13.8, and *α*_H_ = 7.73) originating from MeOH were almost entirely undetectable, indicating that Ba:TiO_2_ in EG-MeOH mixtures predominantly acquires •CHOHCH_2_OH. Notably, compared to pure EG, the EG-MeOH mixture exhibited more pronounced •CHOHCH_2_OH signals, necessitating a deeper analysis of the alkaline-earth-metals-modified TiO_2_ catalyst system.

Theoretical calculations and experimental measurements of material electronic structures indicate that the introduction of alkaline earth metals alters the strength of Ti–O bonds with the TiO_2_ crystal structure and modifies the distribution of localized electrons. Consequently, the Lewis acidic/basic properties of the TiO_2_ surface may undergo significant changes, thereby altering the inherent FLP active site characteristics. The CO_2_ and NH_3_ temperature-programmed desorption (TPD) tests were conducted to elucidate the role of alkaline earth metal introduction on the surface properties of TiO_2_-based materials (Fig. 6e). With the introduction of alkaline earth metal and an increase in their atomic radii, TiO_2_ exhibits higher absorption intensity and larger desorption peak areas compared to pristine TiO_2_ in the CO_2_-TPD. This indicates that introducing an alkaline earth metal into TiO_2_ significantly enhances its Lewis basicity relative to pristine TiO_2_, owing to the increased number of basic sites. Besides, the zeta potential in alkaline solution (0.1 M KOH) shifts toward a positive charge with the addition of alkaline earth metals in TiO_2_ and the increase in their atomic radii (Supplementary Fig. 41). This indicates that surface hydroxyl groups readily undergo protonation, i.e., M:TiO_2_ exhibits stronger surface Lewis basicity compared to pristine TiO_2._

On the other hand, the increase in the number of acidic centers in M:TiO_2_ can be directly attributed to the introduction of alkaline earth metal, which creates strong Lewis acid sites. Especially, the strongest acidity is observed in Ba:TiO_2_. Additionally, the surface acidity of Ba:TiO_2_ was further confirmed by pyridine adsorption infrared spectroscopy (Py-IR). As shown in Fig. 6f, the characteristic peaks at 1443 and 1541 cm^−1^ are attributed to the pyridine adsorbed at the Lewis acid and Brønsted acid sites, respectively, while the absorption peak at 1493 cm^−1^ corresponds to the combination of Lewis acid and Brønsted acid sites, indicating that the acid sites on Ba:TiO_2_ mainly exist in the form of Lewis acid^55^. These results demonstrate that the introduction of alkaline earth metals into TiO_2_ can simultaneously enhance its Lewis acidity and basicity properties, thereby strengthening the surface properties of the FLPs active interface.

To understand the importance of acidic and basic sites in the photocatalytic C–C cross-coupling reaction to produce C_3_ over M:TiO_2_, pyrrole and pyridine (the molar ratio of pyrrole/pyridine to catalyst was 1:20) were used as probe molecules to selectively interact with these sites^56^. Pyrrole, which primarily interacts with basic sites, and pyridine, which binds to acidic sites, were introduced into the catalyst via 12-h stirring adsorption in an ethanol solution of pyrrole/pyridine. As shown in Fig. 6g, the addition of either pyrrole or pyridine completely quenched the photocatalytic activity. Given the atomically dispersed nature of Ba (Figs. 3e–g), it can be inferred that each Ba Lewis acid site is coordinatively surrounded by multiple lattice oxygen Lewis base sites (analogous to the host TiO_2_ crystal structure). This intrinsic spatial proximity, enforced by the host lattice, allows the Ba–O pairs with a length of 2.42 Å to function cooperatively in the simultaneous activation of distinct bonds in the reactants, as evidenced by our DFT calculations (Fig. 2a, b).

**Fig. 7:**
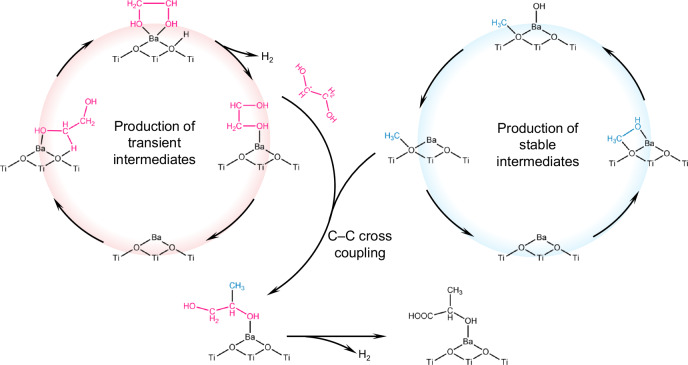
Schematic diagram. Proposed photocatalytic mechanism for C–C cross-coupling reaction to produce C_3_ compounds over Ba:TiO_2_.

Combining the predicted pathway from theoretical calculations with key intermediates and surface acidic/basic properties obtained experimentally, we propose a complete reaction pathway for the photocatalytic EG-MeOH cross-coupling reaction to produce C_3_ products (LA) over M:TiO_2_ (e.g., Ba:TiO_2_) in Fig. 7. First, the frustrated acidic center (Ba) interacts with the electronegative C–OH bond in EG by a bridged hydroxy adsorption, while the frustrated basic center (lattice O^2–^) interacts with the C–H of the methylene group. Upon photoexcitation, the O^2–^ site accumulates photogenerated holes and extracts electrons from the polarized C–H bond, while the Ba captures photogenerated electrons and injects them into the C–O bond, leading to the selective cleavage of the C–H bond and formation of H_2_ and transient intermediates (•CHOHCH_2_OH). Similarly, the selective cleavage of the C–OH bond occurs on MeOH, yielding stable intermediates (*CH_3_) and water. Finally, the •CHOHCH_2_OH accumulated near Ba sites rapidly attacks the *CH_3_ on O^2–^ sites, forming C–C cross-coupling products. After that, the resulting coupled intermediates are readily transformed into lactic acid under alkaline conditions, following successive base-catalyzed and photocatalytic oxidation, and vacating the reaction sites^31^. The rapid vacating and regeneration of the reaction sites facilitate the continuous generation of reactive intermediates, which may account for the enhanced alkyl radical signal observed in the EG-MeOH mixtures (Fig. 6c).

## Discussion

This study demonstrates a solar-driven route for the selective conversion of PET-derived ethylene glycol into C_3_ products, particularly value-added lactic acid, over alkaline-earth-metal-modified TiO_2_ photocatalysts. Incorporation of atomically dispersed Ba species into TiO_2_ enhances the surface FLP characteristics of the catalyst, enabling efficient activation of the C–O bond in methanol and the C–H bond in ethylene glycol. Such cooperative activation promotes selective bond cleavage and generates complementary reaction intermediates, including steady-state *CH_3_ species and transient •CHOHCH_2_OH radicals, thereby facilitating photocatalytic C–C cross-coupling and carbon-chain growth. Combined experimental studies and DFT calculations reveal that the Ba–lattice oxygen FLP sites serve as the dominate active centers, mediating directional charge transfer and enabling high lactic acid activity and selectivity. These findings provide a strategy for solar-driven carbon-growth valorization of plastic-derived oxygenates and highlight the potential of surface FLP engineering in selective photocatalytic cross-coupling reactions.

## Methods

### Materials and reagents

All chemicals used in the present study were commercially available. Titanium butoxide (97%), p-phthalic acid (PTA, 99%), 5,5-dimethyl-1-pyrroline N-oxide (DMPO, 97%), N,N-dimethylformamide (DMF, 99.5%) and phosphoric acid (>85 wt% in H_2_O) were supplied by Aladdin. Methanol (CH_3_OH, 99.5%), ethanol (99.5%), ethylene glycol (EG, 99%), D,L-lactic acid (D,L-LA, ACS), acetonitrile (99.9% for HPLC), perfluorinated resin solution (Nafion-117, 5 wt%), potassium hydroxide (KOH, 95%), magnesium chloride (MgCl_2_, 99%), calcium chloride (CaCl_2_, 99.9%), strontium chloride (SrCl_2_, 98%), and barium chloride dihydrate (BaCl_2_·2H_2_O, 99%) were purchased from Macklin. Deionized water was used for all experiments, unless otherwise specified.

### Preparation of MIL-125(Ti)

MIL-125(Ti) nanoparticles were synthesized by a solvothermal method according to the reported procedure with a slight modification^57^. Briefly, 3 g of PTA was dissolved in 60 mL mixed solution containing 56 mL of DMF and 6 mL of MeOH. Thereafter, 1.2 mL of titanium butoxide was added to the above mixed solution, and the mixture was stirred at room temperature until a clear solution formed. Then, it was sealed in a stainless-steel autoclave (100 mL) and heated at 130 °C for 20 h. The resultant suspension was recovered by centrifugation (8721 × *g*, 5 min) and washed with ethanol three times. The obtained MIL-125(Ti) was dried under vacuum at 60 °C for 12 h and manually ground for the next process.

### Preparation of alkaline-earth metal doing TiO_2_ (M:TiO_2_)

MIL-125(Ti) was well dispersed in MeOH via sonication, followed by the addition of an alkaline-earth metal salt with the designed weight ratio. For example, 100 mg of MIL-125(Ti) was dispersed in 30 mL of MeOH. After that, 60 µL MeOH solution of BaCl_2_·2H_2_O (10 mg mL^−1^) was added to the MIL-125(Ti) suspension, and the suspension was stirred at room temperature in the dark for 3 h. Thereafter, Ba-MIL-125(Ti) nanoparticles were collected with centrifugation at 8721 × *g* for 5 min. The obtained Ba-MIL-125(Ti) nanoparticles were dried at 60 °C for 6 h and ground for the next process.

Ba_1_:TiO_2_ (Ba:TiO_2_) sample was obtained by calcination of the above Ba-MIL-125(Ti) nanoparticles under O_2_ atmosphere at 500 °C for 3 h with a heating rate of 10 °C min^−1^. The TiO_2_ with various metal loadings (including free metal, Mg, Ca, and Sr) was obtained by calcination of the solid at the same conditions. Similarly, Ba:TiO_2_ with various loadings (Ba_0.5_:TiO_2_, Ba_2_:TiO_2_, and Ba_5_:TiO_2_) were prepared by calcinating Ba-MIL-125(Ti). The weight ratios of alkaline-earth metal atoms to TiO_2_ were later determined by inductively coupled plasma atomic emission spectrometry (ICP-AES, Varia 720 ES, Agilent), as shown in Supplementary Table 1.

### General characterization

The morphologies and crystallographic structures of the samples were characterized using a transmission scanning electron microscope (TEM, Talos F200S, Thermo Scientific) at an acceleration voltage of 300 kV. The atom distributions were determined through aberration-corrected high-angle annular dark-field scanning transmission electron microscopy (AC-STEM, Themis Z, Thermo Scientific) and energy-dispersive spectroscopy (EDS, Ultim Max, OXFORD).

Phase structures were characterized by powder X-ray diffraction patterns (PXRD) on a Bruker diffractometer (D8 ADVANCE Da Vinci, Germany) with Cu *K*_α_ radiation (λ = 1.5416 Å) under an operating voltage of 40 kV and a current of 25 mA. Diffraction data were recorded over a 2 theta range from 5° to 80° at room temperature with a step interval of 0.06° and a scan rate of 10° min^−1^. The bonding behavior of the samples was studied using Raman spectroscopy (inVia Reflex, Renishaw) with a 532 nm laser source.

In the XPS (K-Alpha Plus, Thermo Scientific) study, photoelectrons generated by Al *K*_α_ (1486.8 eV) primary radiation (20 kV, 10 mA) were analyzed with a hemispherical analyzer, and core-level XPS for C 1*s*, O 1*s*, Ti 2*p* and Ba 3*d* (Mg 1*s*, Ca 2*s* or Sr 3*d*) were recorded and fitted. The spectra were calibrated using the C 1*s* signal of surface carbon species at 284.8 eV as an internal standard. Quantitative comparisons were conducted through one-way analysis of variance.

The ultraviolet–visible diffuse reflectance spectroscopy (UV–vis DRS) was performed on a Hitachi U-4100 spectrometer. Steady state and transient state photoluminescence (PL) spectra were obtained on an Edinburgh FLS-1000 fluorescence spectrophotometer. Temperature-dependent PL spectra were obtained on an FLS-980 spectrophotometer with excitation wavelength at 350 nm from 77 to 350 K.

Thermogravimetric analysis (TGA) was performed using a PerkinElmer TG-7 instrument, with a heating rate of 10 °C min^−1^ under an air atmosphere. The temperautre programmed desorption (TPD) of NH_3_ and CO_2_ was measured by the Autochem II 2920, a fully automatic chemical adsorption instrument. Pyridine adsorption infrared spectroscopy (Py-IR) was collected by a Bruker Vertex 70 infrared spectrometer. Before testing, all samples were heated to 150 °C under an inert atmosphere for 2 h. Thereafter, the sample was purged with inert gas flow (30 mL min^−1^) for one hour to cool down (reach 30 °C). Pretreated samples were exposed to CO_2_, NH_3_ or pyridine flow (50 mL min^−1^) at 30 °C for 2 h. Then, inert gas flow (30 mL min^−1^) was switched to remove weakly physically adsorbed molecules from the sample surface. For TPD, the investigation proceeded at temperatures ranging from 30 to 500 °C with a heating rate of 5 °C min^−1^. For Py-IR, the signal was collected at 323, 423, and 523 K under inert gas flow (30 mL min^−1^).

### Photocatalytic performance test

The conventional reaction was performed in a 50 mL Pyrex reaction cell. A 300 W Xe lamp (PLS-SXE300D, Beijing Perfectlight) was used as a light source (wavelength: 280–780 nm). Prior to the catalytic measurements, photocatalysts (5 mg) were suspended in 20 mL of CH_3_OH and EG mixed KOH solution (volume ratio of CH_3_OH is 50 vol.%, molar ratio of CH_3_OH to EG is 5:1, concentration of KOH is 4.0 mol L^−1^) under ultrasonication for 10 min. Before the irradiation, the reaction cell was evacuated for 5 min and purged with Ar (99.999%) for 15 min in the dark to remove oxygen from the system. Then, the reactor was placed in a constant temperature water bath at 25 °C and irradiated under an Xe lamp (360 mW cm^−2^) for 2 h. The light intensity was calibrated using a 1919-R optical power meter (Newport). After the reaction, the liquid products were filtered by an NL6 membrane with a pore size of 0.22 µm and analyzed through high-performance liquid chromatography (HPLC), liquid nuclear magnetic resonance (NMR), and liquid chromatography-mass spectrometry (LC-MS). Meanwhile, the gas products were detected by gas chromatography (GC) with Ar as carrier gas. Notably, each reaction was repeated three times and averaged to ensure data repeatability. The data in the graphs with error bars were expressed as the mean ± standard deviation of the sample population.

Since the conversion efficiency of the reactant (CH_3_OH and EG) in the system is lower than 1%, conducting a comprehensive quantitative analysis of consumed reactants and subsequently calculating the selectivity for D,L-LA is challenging. Instead, we evaluated selectivity by calculating the carbon ratio across all products (D,L-LA, formate and glycolic acid). The carbon selectivity (C Sel.) of D,L-LA was calculated through the following equation:

1 $${{\rm{C}} \; {\rm{Sel}}}.\left(\%\right)=\frac{3\times {N}_{({\rm{D}},{\rm{L}}-{\rm{LA}})}}{3\times {N}_{({\rm{D}},{\rm{L}}-{\rm{LA}})}+{N}_{({\rm{formate}})}+2\times {N}_{({\rm{glycolic}}\, {\rm{acid}})}}\times 100\%$$

where *N*_(D,L-LA)_, *N*_(formate)_, and *N*_(glycolic acid)_ are the moles of D,L-LA, formate, and glycolic acid produced in the solution after the reaction.

### Analytical methods

For the CH_3_OH and EG to D,L-LA experiment, the liquid reaction mixture was filtered. And the 100 µL of obtained solution was added to the 400 µL of H_2_SO_4_ aqueous solution (0.5 mol L^−1^) for neutralization, and diluted with another 500 µL of deionized water. The D,L-LA concentration during the reaction was analyzed by HLPC on an Acchrom S6000 system at 37 °C. The HLPC was equipped with a UV–vis detector at 220 nm and a 4.6 × 250 mm Alphasil ES-C18 column. Products were separated and quantified by isocratic HPLC using a 0.12% aqueous phosphoric acid/acetonitrile (98:2, v/v) mobile phase at a flow rate of 0.425 mL min^−1^ over 12 min. Quantification and identification were carried out via external calibration based on standard curves of commercially available reactants and products^58^. A small background signal of glycolic acid may be present before photocatalysis due to partial oxidation of ethylene glycol under aerobic alkaline conditions; this contribution was subtracted during quantitative analysis.

^1^H NMR experiment was performed on a Bruker Avance 300 MHz NMR spectrometer without solvent (water) suppression. The obtained solution (after neutralization and dilution, 540 µL) was mixed with D_2_O (60 µL) for ^1^H NMR analysis (D1 = 20 s; scan number, 128). For the quantitative analysis, product concentration was calculated from their peak area.

The product’s qualitative analysis was further carried out using LC-MS (SCIEX, Triple TOF 5600). The eluent was an aqueous formic acid solution (0.4 vol%), and detection was performed in positive mode.

Products were injected into a GC (GC-2018, Shimazu) equipped with a TCD detector and a 5 Å molecular sieve column (Ar as the carrier gas).

In general, in the one-step excitation process, producing one D,L-LA from EG needs four holes. Then the AQY can be calculated according to the Eq. 2^46^:

2 $${{\rm{AQY}}}(\%)=	 \frac{{\rm{number}} \, {\rm{of}} \, {\rm{reacted}} \, {\rm{electrons}}}{{\rm{number}} \, {\rm{of}} \, {\rm{incident}} \, {\rm{photons}}}\times 100\%\\=	 \frac{{\rm{number}} \, {\rm{of}} \, {\rm{D}},{\rm{L}}-{\rm{LA}} \, {\rm{molecules}}\times 4}{{\rm{number}} \, {\rm{of}} \, {\rm{incident}} \, {\rm{photons}}}\times 100\%$$

Take AQY@350 nm of Ba:TiO_2_ as an example, AQY calculated from Eq. 2:

$${{\rm{N}}}=\frac{{\rm{P}}\times \lambda \times {\rm{t}}}{{\rm{h}}\times {\rm{c}}}=\frac{0.2205\times 350\times {10}^{-9}\times 3600}{6.626\times {10}^{-34}\times 3\times {10}^{8}}=1.40\times {10}^{21}$$

$${{\rm{AQY}}} (\% ) = \frac{{\rm{N}}_{\rm{A}} \times n \times 4}{\rm{N}} \times 100 \%= 2.81 \% $$

The power densities of the monochromatic light at wavelengths of 350, 365, and 380 nm are 22.9, 26.5, and 28.5 mW cm^–2^, respectively.

### Outdoor amplification experiment

Assembly of the flow reactor. Firstly, 50 mg of Ba:TiO_2_ photocatalyst and 300 µL of Nafion-117 solution (5 wt%) were dispersed in a mixed solvent of ethanol (15 mL) and H_2_O (15 mL). The mixture was fully dispersed after 30 min ultrasonication, yielding a dispersion system. Thereafter, the dispersed system was uniformly deposited on the quartz tubes (external diameter: 10 mm, inner diameter: 7 mm, length: 250 mm) through a dip-coating. Finally, the deposited quartz tubes were dried at 80 °C for 12 h and interconnected using polytetrafluoroethylene tubing and peristaltic pump tubing to construct the final flow system of the flow reactor.

Long-term operation for CH_3_OH and EG cross-coupling over Ba:TiO_2_. The 100 mL outdoor amplification experiment (50 mL of CH_3_OH, 13.75 mL of EG, 36.25 mL of H_2_O, 22.4 g of KOH, and 50 mg of Ba:TiO_2_) was carried out in a specially designed flow-through quartz reactor using a peristaltic pump. The solution was purged with Ar for at least 20 min to remove O_2_. Then the solution was circulated within the tubular system via the peristaltic pump under the sunlight. Each hour, the actual light intensity on the reactor surface was measured using a light intensity meter. After sunset, the samples were taken to detect the D,L-LA concentration in the solution.

### Bandgap calculation using UV–vis DRS

Diffuse reflectance spectra were converted using the Kubelka-Munk function:

3 $${\alpha }=\frac{{(1-R)}^{2}}{2R}\times 100\%$$

and the optical band gaps were estimated from Tauc plots based on below equation^46^:

4 $$\alpha {\rm{h}}\nu={\rm{A}}{({\rm{h}} \nu -{E}_{{\rm{g}}})}^{\frac{{\rm{n}}}{2}}$$

where *α* is the absorption coefficient, *R* denotes the reflectance relative to a standard reference, h*ν* represents the photon energy (h is the Planck constant, and the *ν* is the light frequency), A is a proportionality constant, and *E*_g_ corresponds to the optical band-gap energy. The exponent *n* depends on the nature of the electronic transition, with *n* = 1 and 4 corresponding to direct and indirect transitions, respectively.

### Photo-electrochemical measurements

Work electrodes were prepared on indium-tin oxide-coated conductive glass (ITO, 20 × 10 × 1.1 mm^3^), which had been cleaned with ethanol and H_2_O under sonication for 5 min, respectively, followed by drying with a high-pressure nitrogen flow (99.999%). In detail, the sample (8 mg) was ultrasonically dispersed in an ethanol solution (600 µL ethanol, 300 µL H_2_O, and 10 µL Nafion-117 solution) to form a homogeneous ink. The ink (50 µL) was uniformly deposited on a piece of ITO glass to act as the working electrode. The electrochemical properties were measured using an electrochemical workstation (CHI 660D) in a three-electrode cell under light irradiation, with a platinum foil and an Ag/AgCl (3.5 M KCl) electrode as the counter and reference electrodes, respectively. A nitrogen-saturated aqueous solution of Na_2_SO_4_ (0.2 M, pH 6.4 ± 0.15) was used as the electrolyte. The exposed working electrode was fixed at 1 cm^2^. The Ag/AgCl electrode was calibrated with respect to the reversible hydrogen electrode as Eq. 5:

5 $${E}_{{{\rm{RHE}}}}={E}_{{{\rm{Ag}}}/{{\rm{AgCl}}}}+0.059 \, {{\rm{pH}}}+0.205$$

EIS was conducted over a frequency range from 100 kHz to 10 Hz with a sinusoidal amplitude of 10 mV, and 10 min of open circuit potential (OCP) conditioning. All electrochemical tests were carried out in a Faraday cage to avoid interference. Photocurrent measurements were performed using a 100 W LED lamp (PLS-LED 100 C, Beijing Perfectlight) emitting 365 nm light. The photo-responsive signals of the samples were collected under chopped light with a bias potential of 1.6 V vs. RHE.

### In situ electron spin resonance (ESR) measurement

In situ ESR was measured on CIQTEK EPR200-Plus with continuous-wave X-band frequency at room temperature. Firstly, 5 mg of Ba:TiO_2_ were ultrasonically dispersed in the mixture of 20 mL of reaction (10 mL of CH_3_OH, 2.75 µL of EG, 6.25 mL of H_2_O, and 4.48 g of KOH). Then, 2 mL of the mixed solution and 10 µL of DMPO were added to a special quartz vial with a rubber stopper. Additionally, Ar was slowly bubbled into the solution to keep it oxygen-free. Thereafter, the quartz vial was exposed to light from the Xe lamp (300 W) for 5 min. Finally, the electron paramagnetic resonance signals were obtained using a capillary tube to draw the mixed solution after the above reaction. Notably, the fitting of •R radicals was carried out on the EPR-ProPr software (ver. 5.4.0). Meanwhile, the measurements without light irradiation (dark), and no addition of CH_3_OH and EG samples, were measured under the same operation process.

### In situ attenuated total reflection Fourier transform infrared spectroscopy (ATR-FTIR) measurements

The measurements of the in situ ATR-FTIR were carried out on a customized photo-electrochemical cell (Gaossunion, Supplementary Fig. 42). A single reflection ATR crystal (SCI Materials Hub) was mechanically polished using progressively finer alumina slurries (5, 0.3, and 0.05 µm) as polishing agents. The wafer was ultrasonically cleaned in H_2_O for 5 min, then dried under high-pressure nitrogen. A homogeneous ink was obtained by adding 8 mg of the sample to 300 µL of H_2_O and 600 µL of ethanol with 10 µL of Nafion-117 solution (5 wt%) under sonication for 30 min. Thereafter, 100 µL of the ink was evenly coated on the wafer and dried in an oven (60 °C) for 6 h.

In situ FTIR spectra were acquired in p-polarized using a Thermo Scientific Nicolet iS50 spectrometer equipped with a liquid nitrogen-cooled MCT-A detector. Spectral acquisition was carried out at a resolution of 4 cm^−1^ with 32 accumulations for each spectrum. The reaction cell was filled with a mixture of methanol (15 mL), ethylene glycol (4.13 mL), water (10.87 mL), and KOH (6.72 g), after which the Ba:TiO_2_-coated wafer was immersed. The cell was purged with argon for 30 min to remove dissolved oxygen. A reference background spectrum was recorded before illumination. Time-resolved spectra were then collected after initiating light irradiation of the Ba:TiO_2_-coated wafer, with spectra acquired at 1 min intervals over a total period of 15 min^59^.

### Transient absorption spectroscopy (TAS) measurements

TAS measurements were carried out using a Helios pump-probe spectrometer (Ultra-fast Systems LLC) coupled with a femtosecond laser source (Coherent). Excitation at 350 nm was produced through an optical parametric amplifier (TOPAS-800-fs) driven by the 800-nm output of the regenerative amplifier. A white-light continuum probe covering 470–890 nm was generated by focusing a portion of the 800-nm laser pulses (~10 µJ) into a 2-mm sapphire crystal. The temporal evolution of the transient signals was controlled by a motorized optical delay stage that adjusted the arrival time between the pump and probe pulses. During measurements, the excitation and probe beams were spatially overlapped on the sample surface, and the transmitted probe signal was detected using a charge-coupled device detector. The two beams were aligned with an intersection angle of 54.7° to ensure reproducible signal collection. For each sample, 5–10 successive scans were recorded and averaged to improve the signal-to-noise ratio prior to data analysis. The temporal delay between the pump and probe pulses was controlled using a motorized optical delay stage positioned in the pump pathway. Pump excitation was periodically interrupted with a mechanical chopper operating at half of the laser repetition frequency to enable synchronized signal acquisition. The optimal number of kinetic components in the sequential fitting model was determined from the improvement of fitting performance evaluated using the corrected Akaike information criterion^60^.

### Theoretical calculation

The free energy profiles of D,L-LA (C_3_H_6_O_3_) production on pristine TiO_2_ and M:TiO_2_ (M=Mg, Ca, Sr, Ba) were investigated by the Vienna ab-initio simulation package with the revised Perdew–Burke–Ernzerh functional of (RPBE) of the generalized gradient approximation. The PAW pseudo-potential was used to describe the interaction between the ionic core and valence electrons. Considering the atomic radius of Ba and the structural characteristics of the TiO_2_ crystal, a bulk Ba-doped TiO_2_ model was constructed by substituting one Ti atom with a Ba atom in a 2 × 2 × 1 anatase TiO_2_ supercell (Supplementary Fig. 43a), yielding a six-coordinated Ba species. The anatase TiO_2_ surface was represented by its typical and stable (1 0 1) facet, modeled using a 2 × 3 (1 0 1) supercell composed of six O–Ti–O layers. Based on the local structure of the TiO_2_ (1 0 1) surface, Ba is thermodynamically favored to occupy a five-oxygen-coordinated site (Supplementary Fig. 43b), forming five-coordinated Ba species. Consequently, the average coordination number of Ba in the overall TiO_2_ sample is between 5 and 6, consistent with the experimental EXAFS fitting result of 5.88. Given the dominant role of surface Ba species in surface catalytic reactions, bulk Ba species were not placed in the deep subsurface layers, and only surface Ba sites were employed in the calculations of the surface reaction pathway. Besides, given that bulk photoexcitation in photocatalysts is predominantly governed by the bulk structure rather than the surface structure, the influence of surface Ba species on bulk photoexcitation was neglected in the PDOS calculations. Accordingly, surface Ba species were employed in modeling surface catalytic reactions, while bulk Ba species were used to evaluate bulk photoexcitation properties. Geometry optimizations were performed at the gamma point using a plane-wave cutoff energy of 450 eV and a self-consistent convergence criterion of 1.0 × 10^–5^ eV. After structure relaxation, PDOS and charge-density-difference analyses for the adsorbed intermediates were evaluated using a Monkhorst-Pack k-point grid of 2 × 3 × 1 with the same energy cutoff and convergence settings. The optimized atomic configuration employed for the PDOS and charge-density-difference analyses are provided in Supplementary Data 1–7. The Gibbs free-energy variations (Δ*G*) for the elementary reaction steps were estimated within the standard hydrogen electrode framework according to the following formula^61,62^:

6 $$G=E+{\mathrm{ZPE}}{{{-}}}{{\rm{T}}}S$$

where *E* denotes the electronic energy, ZPE corresponds to the zero-point energy correction, and *S* represents the entropy contribution at *T* = 298.15 K.

## Supplementary information

Supplementary Information

Description of Additional Supplementary Files

Supplementary Data 1–7

Transparent Peer Review File

## Source data

Source Data

## Data Availability

The data that support the findings of this study are available within the article and Supplementary Information. Source data are provided with this paper or available from the corresponding author upon request. Source data are provided with this paper.
