# Supplementary material for: Lactic acid photosynthesis via C–C cross-coupling over atomically dispersed Ba
Source: Nat Commun. 2026 May 29;17:6984. doi: 10.1038/s41467-026-73727-4 (PMC13392379; doi:10.1038/s41467-026-73727-4)
Supplement: Supplementary file 1 — Supplementary Information [file 41467_2026_73727_MOESM1_ESM.pdf]

Supplementary Information

Lactic acid photosynthesis via C–C cross-coupling over atomically dispersed Ba

Wei Wang[a], Yonghua Tang[b], ZhuiZhui Su[a], Huanmin Liu[a], You-Nian Liu[c], Dingguo Tang[d], and Peng Zhou*[a,e,f]

[a] School of Environment and Energy, Peking University Shenzhen Graduate School, Shenzhen, Guangdong 518055, China

[b] School of Physics and Optoelectronics, Xiangtan University, Xiangtan 411105, Hunan Province, China

[c] College of Chemistry and Chemical Engineering, Central South University, Changsha 410083, China

[d] Key Laboratory of Catalysis and Energy Materials Chemistry of Ministry of Education, School of Chemistry and Materials Science, South-Central Minzu University, Wuhan 430074, China

[e] Eco-environment and Resource Efficiency Research Laboratory, School of Environment and Energy, Peking University Shenzhen Graduate School, Shenzhen 518055, China

[f] Guangdong Provincial Key Lab of Nano-Micro Material Research, Peking University Shenzhen Graduate School, Guangdong 518055, PR China

* *Corresponding author*: P. Zhou (pengzhou1209@pku.edu.cn)

Contents

Supplementary Fig. 1 Charge density difference mapping between *OHCHCH$_2$OH intermediates and catalyst surface generated by VESTA visualization software [1]. (a) pristine TiO$_2$, (b) Ba:TiO$_2$. The skyblue and yellow isosurfaces stand for the negative and positive charge, respectively. The isosurface of charge density is set to 0.002 e Å^{-3}. The pink balls, brown balls, red balls, light blue balls and green balls represent H, C, O, Ti, and Ba atom, respectively. 5

Supplementary Fig. 2 Optimized structures of *CH$_3$ intermediates upon catalyst surface, the images generated by VESTA visualization software [1]. (a) pristine TiO$_2$, (b) Ba:TiO$_2$. The pink balls, brown balls, red balls, light blue balls and green balls represent H, C, O, Ti, and Ba atom, respectively. 5

Supplementary Fig. 3 Energy barriers of *CHOHCH$_2$OH intermediate formation on TiO$_2$, and Mg, Ca, Sr or Ba-modified TiO$_2$. 6

Supplementary Fig. 4 Free energy profile of formic acid production on Ba:TiO$_2$ at pH = 7 and U = 0 V vs. SHE. 6

Supplementary Fig. 5 Free energy profile of glycolic acid production on Ba:TiO$_2$ at pH = 7 and U = 0 V vs. SHE. 6

Supplementary Fig. 6 Projected density of states (PDOS) of the O 2*p* orbitals for surface oxygen atoms........7

Supplementary Fig. 7 Thermogravimetric analysis result of Ba^{2+}-MIL-125(Ti) in oxygen atmosphere. 7

Supplementary Fig. 8 Electron spin resonance (ESR) spectrum of Ba:TiO$_2$. 8

Supplementary Fig. 9 Raman spectrum of Ba:TiO$_2$, laser wavelength: 532 nm. 8

Supplementary Fig. 10 X-ray photoelectron spectroscopy (XPS) depth profiling analysis of Ba:TiO$_2$. (a) O 1*s*, (b) Ba 3*d*, (c) Ti 2*p* and (d) elements distribution from 0 to 8 nm. These arrows indicate the direction in which the XPS detection depth value increases. 9

Supplementary Fig. 11 Focused ion beam-scanning electron microscopy (FIB-SEM) cross-sectional elemental distribution analysis. (a) Schematic diagram of FIB-SEM, (b) cross-sectional SEM image, (c) cross-sectional EDS image and (d) line scan EDS element distribution (yellow line in Supplementary Fig. 11b). 10

Supplementary Fig. 12 Aberration-corrected scanning transmission electron microscopy (AC-STEM) image of Ba:TiO$_2$, inset: 3D color map of the cyan-outline region, the protruding tip being Ba single atoms. 10

Supplementary Fig. 13 N$_2$ adsorption isotherms of (a) MIL-125(Ti) and (b) TiO$_2$, and (c) pore size distribution of corresponding catalysts........11

Supplementary Fig. 14 X-ray absorption near edge structure (XANES) spectra of Ba K-edge for Ba:TiO$_2$ and BaO........12

Supplementary Fig. 15 Ba K-edge extended X-ray absorption fine structure (EXAFS, points) and fitting curve (line) for Ba:TiO$_2$, shown in k^3-weighted K-space........12

Supplementary Fig. 16 Raman spectra of TiO$_2$ samples, laser wavelength: 532 nm, laser intensity: 1%, exposure time: 10 s. These arrows represent the direction of peak shift. 13

Supplementary Fig. 17 Optimized structures of bulk Ba:TiO$_2$. The red ball, light blue ball and green ball represent O, Ti, and Ba atom, respectively. The images generated by VESTA visualization software [1]. The values pointed to by these arrows represent the lengths of corresponding chemical bonds........13

Supplementary Fig. 18 High-resolution X-ray photoelectron spectroscopy (XPS) spectra of (a) O 1*s* and (b) Ti 2*p* on various TiO$_2$ samples. These arrows represent the direction of peak shift. 14

Supplementary Fig. 19 High performance liquid chromatography (HPLC) of standard solutions of (a) D,L-lactic acid, (b) formic acid, and (c) glycolic acid. Corresponding calibration curves of (d) D,L-lactic acid, (e) formic acid, and (f) glycolic acid. The arrows indicate increasing concentrations of the standard solutions. 15

Supplementary Fig. 20 The apparent quantum yield (AQY) of Ba:TiO$_2$ sample. Reaction conditions: 5 mg of catalysts, 20 mL of reaction mixture (50 vol% methanol, ethylene glycol with molar ratio to methanol 1:5, and KOH with

concentration is 4.0 M), T = 25 °C, 1 bar Ar, t = 2 h. Light intensity: 22.9 mW cm^{-2} (350 nm); 26.5 mW cm^{-2} (365 nm); 28.5 mW cm^{-2} (380 nm). Irradiated area: 9.6 cm^2. A minimum of three replicate measurements was performed for each material group to ensure reproducibility..16

Supplementary Fig. 21 D,L-lactic acid (LA) and H_2 production rate over different amount of methanol (MeOH) addition in the reaction system; the bar chart uses the left vertical axis, while the point plot uses the right vertical axis, as indicated by these arrows. Reaction conditions: 5 mg of catalysts, 20 mL of reaction mixture (ethylene glycol with molar ratio to MeOH 1:5, and KOH with concentration is 4.0 M), T = 25 °C, 1 bar Ar, t = 2 h. A minimum of three replicate measurements was performed for each material group to ensure reproducibility. 17

Supplementary Fig. 22 D,L-lactic acid (LA) and H_2 production rate over different molar ration of methanol (MeOH) to ethylene glycol (EG) in the reaction system; the bar chart uses the left vertical axis, while the point plot uses the right vertical axis, as indicated by these arrows. Reaction conditions: 5 mg of catalysts, 20 mL of reaction mixture (50 v/v% of MeOH, and KOH with concentration is 4.0 M), T = 25 °C, 1 bar Ar, t = 2 h. A minimum of three replicate measurements was performed for each material group to ensure reproducibility. 18

Supplementary Fig. 23 D,L-lactic acid (LA) and H_2 production rate over different concentration of KOH in the reaction system; the bar chart uses the left vertical axis, while the point plot uses the right vertical axis, as indicated by these arrows. Reaction conditions: 5 mg of catalysts, 20 mL of reaction mixture (50 vol% methanol, and ethylene glycol with molar ratio to methanol 1:5), T = 25 °C, 1 bar Ar, t = 2 h. A minimum of three replicate measurements was performed for each material group to ensure reproducibility. 19

Supplementary Fig. 24 D,L-lactic acid (LA) and H_2 production rate over different incorporation amount of Ba in TiO_2; the bar chart uses the left vertical axis, while the point plot uses the right vertical axis, as indicated by these arrows. Reaction conditions: 5 mg of catalysts, 20 mL of reaction mixture (50 vol% methanol, ethylene glycol with molar ratio to methanol 1:5, and KOH with concentration is 4.0 M), T = 25 °C, 1 bar Ar, t = 2 h. A minimum of three replicate measurements was performed for each material group to ensure reproducibility. 20

Supplementary Fig. 25 High performance liquid chromatography (HPLC) spectra of (a) TiO_2, (b) Ba:TiO_2 in dark, (c) Ba^{2+}-TiO_2, and (d) Ba:TiO_2, corresponding to Fig. 4b. Minor invariant peaks observed at specific retention times are attributed to system-related background signals or trance impurities are not associated with photocatalytic reaction products...21

Supplementary Fig. 26 High performance liquid chromatography (HPLC) spectra of (a) Mg:TiO_2, (b) Ca:TiO_2, (c) Sr:TiO_2, and (d) Ba:TiO_2, corresponding to Fig. 4c. Minor invariant peaks observed at specific retention times are attributed to system-related background signals or trance impurities are not associated with photocatalytic reaction products...22

Supplementary Fig. 27 High performance liquid chromatography (HPLC) spectra of (a)$Ba_{0.5}$:TiO_2, (b) Ba_1:TiO_2, (c) Ba_2:TiO_2, and (d) Ba_5:TiO_2, corresponding to Supplementary Fig. 24. Minor invariant peaks observed at specific retention times are attributed to system-related background signals or trance impurities are not associated with photocatalytic reaction products. 23

Supplementary Fig. 28 D,L-lactic acid (LA) and H_2 production rate on Ba:TiO_2 without or with ethylene glycol and methanol. Reaction conditions: 5 mg of catalysts, 20 mL of H_2O or reaction mixture (50 vol% methanol, ethylene glycol with molar ratio to methanol 1:5, and KOH with concentration is 4.0 M), T = 25 °C, 1 bar Ar, t = 2 h. A minimum of three replicate measurements was performed for each material group to ensure reproducibility. 24

Supplementary Fig. 29 Outdoor solar irradiation conditions, collected at 22°35’49’’ N, 113°58’22’’ E, and on Aug 10, 2025. 25

Supplementary Fig. 30 Continuous-flow reaction under a full day of outdoor sunlight exposure (from 9:00 to 17:00), collected at 22°35’49’’ N, 113°58’22’’ E, and on Aug 10, 2025. 25
Supplementary Fig. 31 Ultraviolet-visible diffuse reflectance spectroscopy (UV–vis DRS) spectra of various TiO_2 samples. 26
Supplementary Fig. 32 Tauc plots of various TiO_2 samples. 26
Supplementary Fig. 33 Valence band-XPS (VB-XPS) spectra of various TiO_2 samples. 27
Supplementary Fig. 34 Band structure of TiO_2 and M:TiO_2 calculated by valence band-XPS and band gap, which potential (E) vs. reversible hydrogen electrode (RHE). 27
Supplementary Fig. 35 Steady state photoluminescence (PL) spectra of TiO_2 and M:TiO_2 samples (λ_{ex}: 350 nm). 28
Supplementary Fig. 36 Electrochemical impedance spectroscopy (EIS) plots of TiO_2 samples in Nyquist formats with corresponding fitting data collected over a period of 30 min (after 10 min open-circuit potential stabilization) over a range of frequency from 100 kHz to 10 mHz. Inset: enlarged view of Nyquist plots with Z’ range of 0–4 × 10^3 Ω cm^{-2}, and equivalent circuit diagram for fitting Nyquist plots (R_s: the solution resistance, C_{dl}: double-layer capacitance; R_{ct}: and charge transfer resistance). 29
Supplementary Fig. 37 Transient photocurrent density-time profiles of TiO_2 samples at 1 V (vs. RHE) in 0.2 M Na_2SO_2 under 100 W LED lamp illumination (365 nm), the measurement without iR-correction. 30
Supplementary Fig. 38 In-situ attenuated total reflection Fourier transform infrared spectroscopy (ATR-FTIR) spectra over neat ATR crystal, reaction conditions: 5 mg of photocatalysts, 50 vol% MEOH, molar ratio of MeOH to EG is 5:1, concentration of KOH is 4.0 M, T = 25 °C, 1 bar Ar, and Xe lamp irradiation. 31
Supplementary Fig. 39 High performance liquid chromatography (HPLC) results of the photocatalytic C–C cross-coupling to produce LA system using TiO_2 as a catalyst after a 2-h reaction. 31
Supplementary Fig. 40 5,5 dimethyl-1-pyrroline N-oxide (DMPO)-trapping in-situ electron spin resonance (ESR) experiments. 32
Supplementary Fig. 41 Zeta potential of pristine TiO_2 and M:TiO_2 in neutral water (pH 6.8 ± 0.08) and 0.1 M KOH, ($n \geq 3$). 32
Supplementary Fig. 42 Image of custom-built photocatalytic reactor for in situ attenuated total reflection Fourier transform infrared spectroscopy studies. 33
Supplementary Fig. 43 Optimized structures of (a) bulk and (b) surface incorporation of Ba upon TiO_2. The red balls, light blue balls and green balls represent O, Ti, and Ba atom, respectively. The images generated by VESTA visualization software [1]. 33
Supplementary Table 1. Load amount of alkaline earth metals elements in TiO_2 34
Supplementary Table 2. Production rates of TiO_2 and M:TiO_2. Reaction conditions: 5 mg of catalysts, 20 mL of reaction mixture (ethylene glycol with molar ratio to CH_3OH 1:5, and KOH with concentration is 4.0 M), T = 25 °C, 1 bar Ar, t = 2 h. A minimum of three replicate measurements was performed for each material group to ensure reproducibility. 34
Supplementary Table 3. Transient state photoluminescence fitting results of various samples 35
Supplementary Table 4. Electrochemical impedance spectroscopy (EIS) fitting results of various samples 35
Reference 36

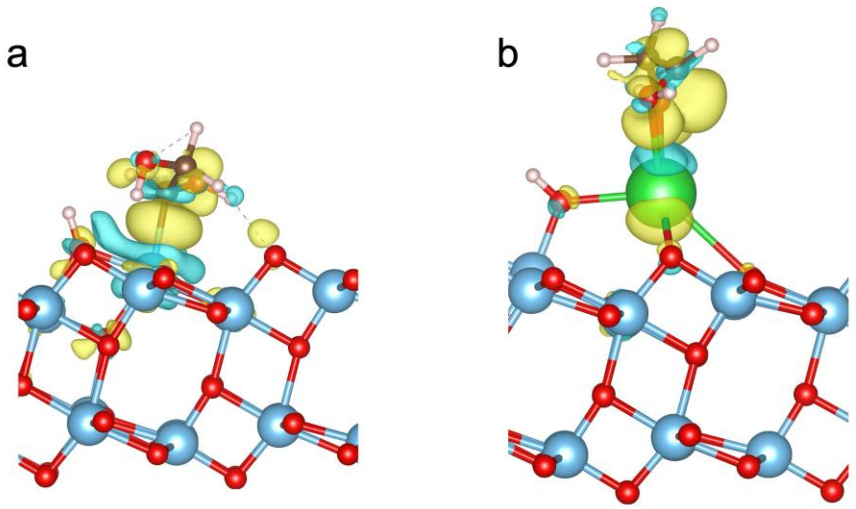

Supplementary Fig. 1 Charge density difference mapping between *$OHCHCH_2OH$ intermediates and catalyst surface generated by VESTA visualization software [1]. (a) pristine TiO_2, (b) Ba:TiO_2. The skyblue and yellow isosurfaces stand for the negative and positive charge, respectively. The isosurface of charge density is set to 0.002 e $Å^{-3}$. The pink balls, brown balls, red balls, light blue balls and green balls represent H, C, O, Ti, and Ba atom, respectively.

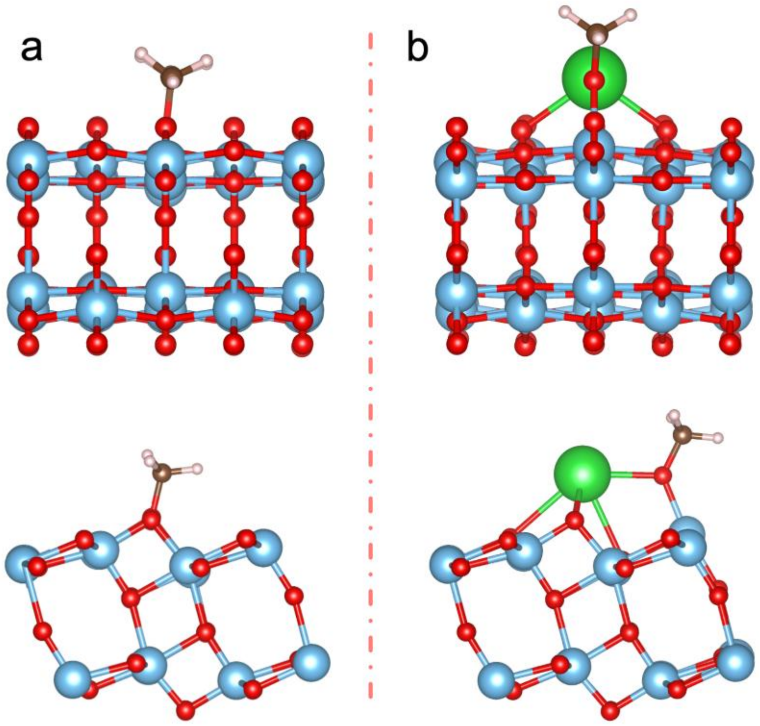

Supplementary Fig. 2 Optimized structures of *CH_3 intermediates upon catalyst surface, the images generated by VESTA visualization software [1]. (a) pristine TiO_2, (b) Ba:TiO_2. The pink balls, brown balls, red balls, light blue balls and green balls represent H, C, O, Ti, and Ba atom, respectively.

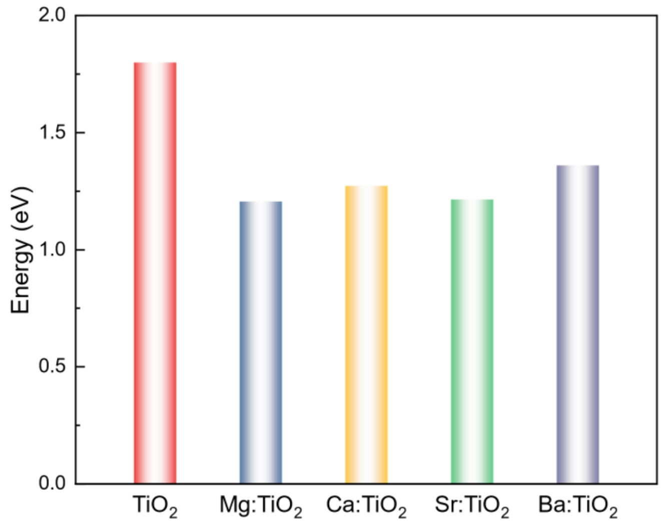

Supplementary Fig. 3 Energy barriers of *CHOHCH$_2$OH intermediate formation on TiO$_2$, and Mg, Ca, Sr or Ba-modified TiO$_2$.

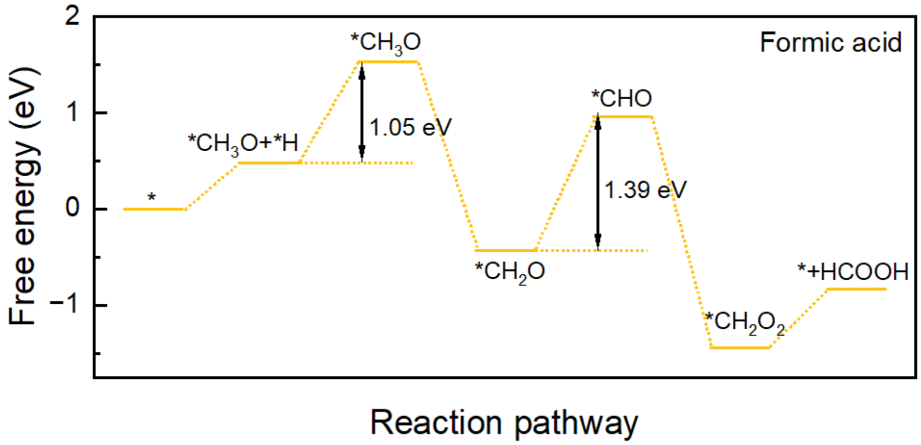

Supplementary Fig. 4 Free energy profile of formic acid production on Ba:TiO$_2$ at pH = 7 and U = 0 V vs. SHE.

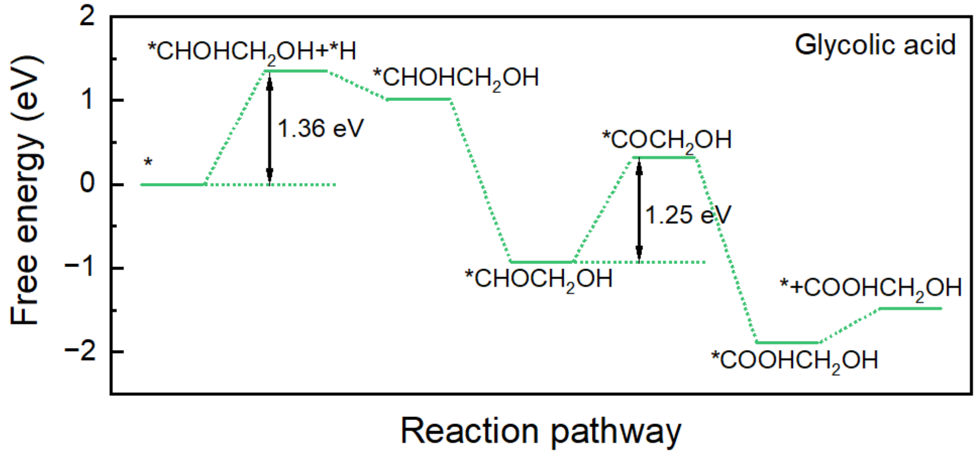

Supplementary Fig. 5 Free energy profile of glycolic acid production on Ba:TiO$_2$ at pH = 7 and U = 0 V vs. SHE.

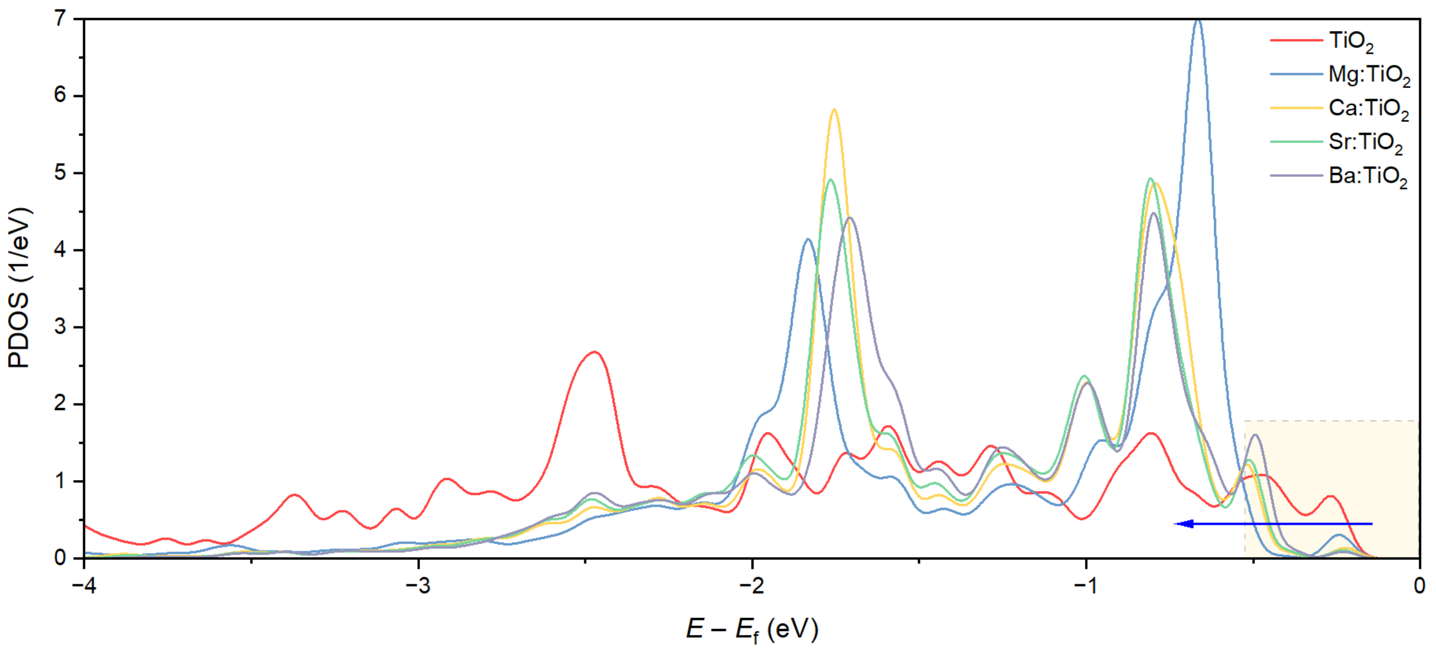

Supplementary Fig. 6 Projected density of states (PDOS) of the O 2*p* orbitals for surface oxygen atoms.

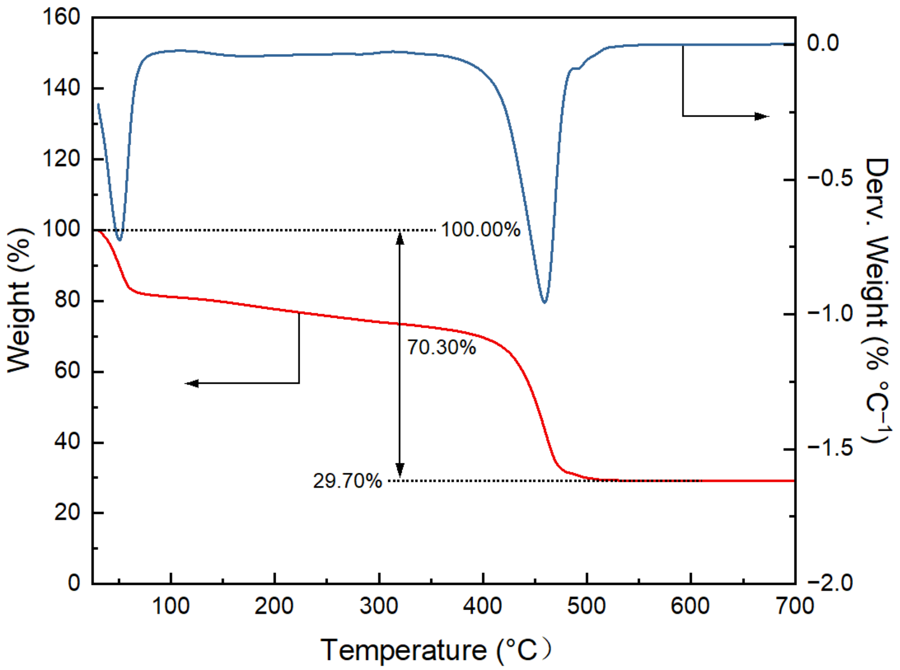

Supplementary Fig. 7 Thermogravimetric analysis result of Ba^{2+}-MIL-125(Ti) in oxygen atmosphere.

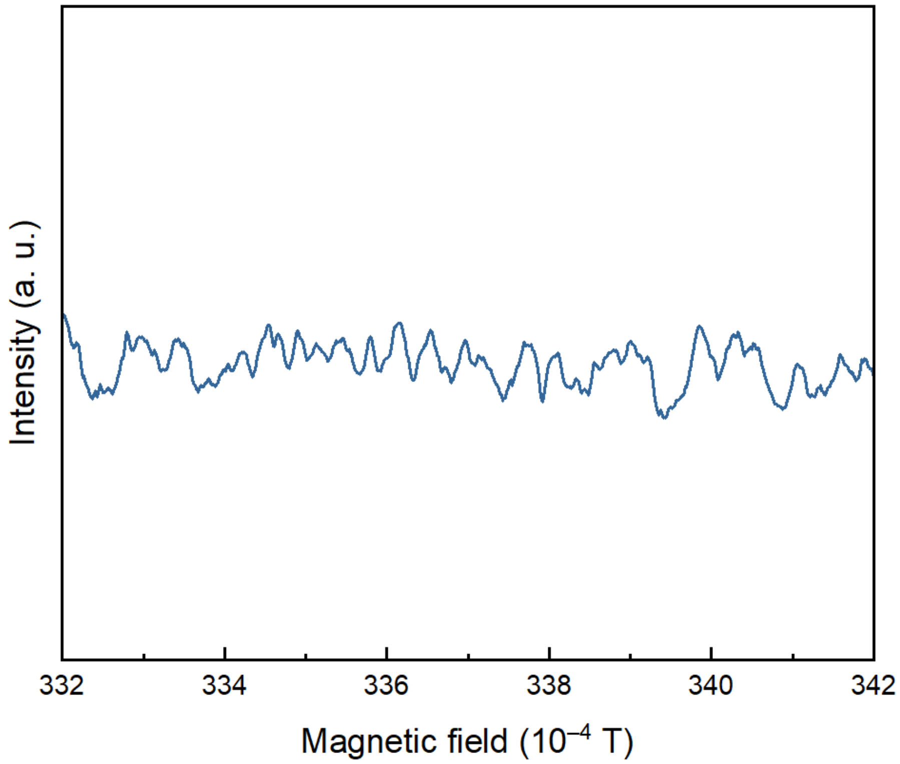

Supplementary Fig. 8 Electron spin resonance (ESR) spectrum of Ba:TiO_2.

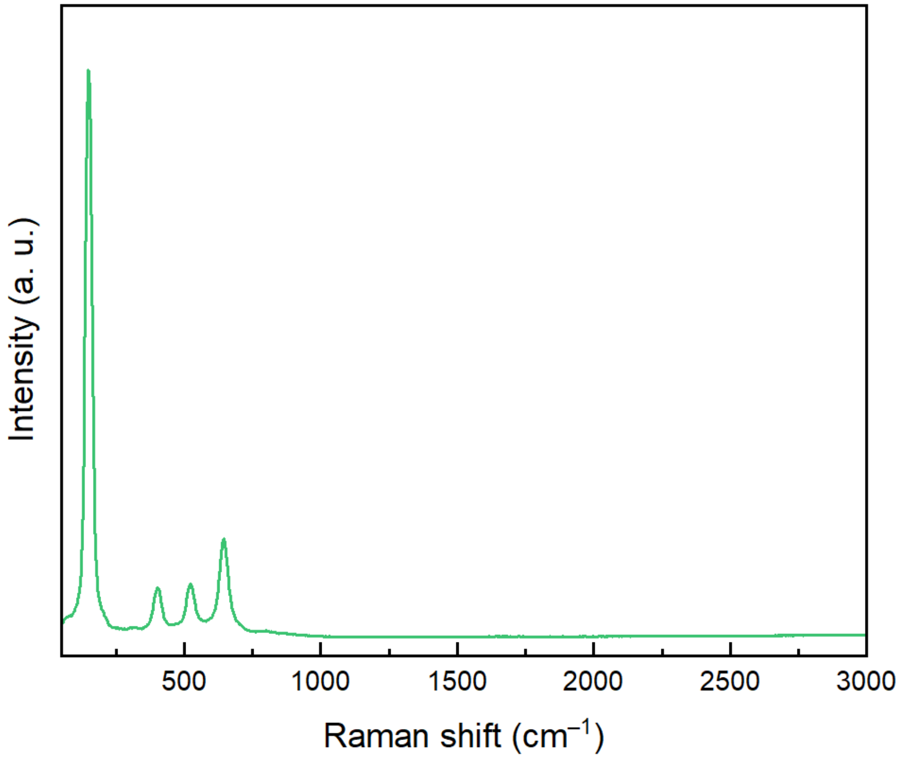

Supplementary Fig. 9 Raman spectrum of Ba:TiO_2, laser wavelength: 532 nm.

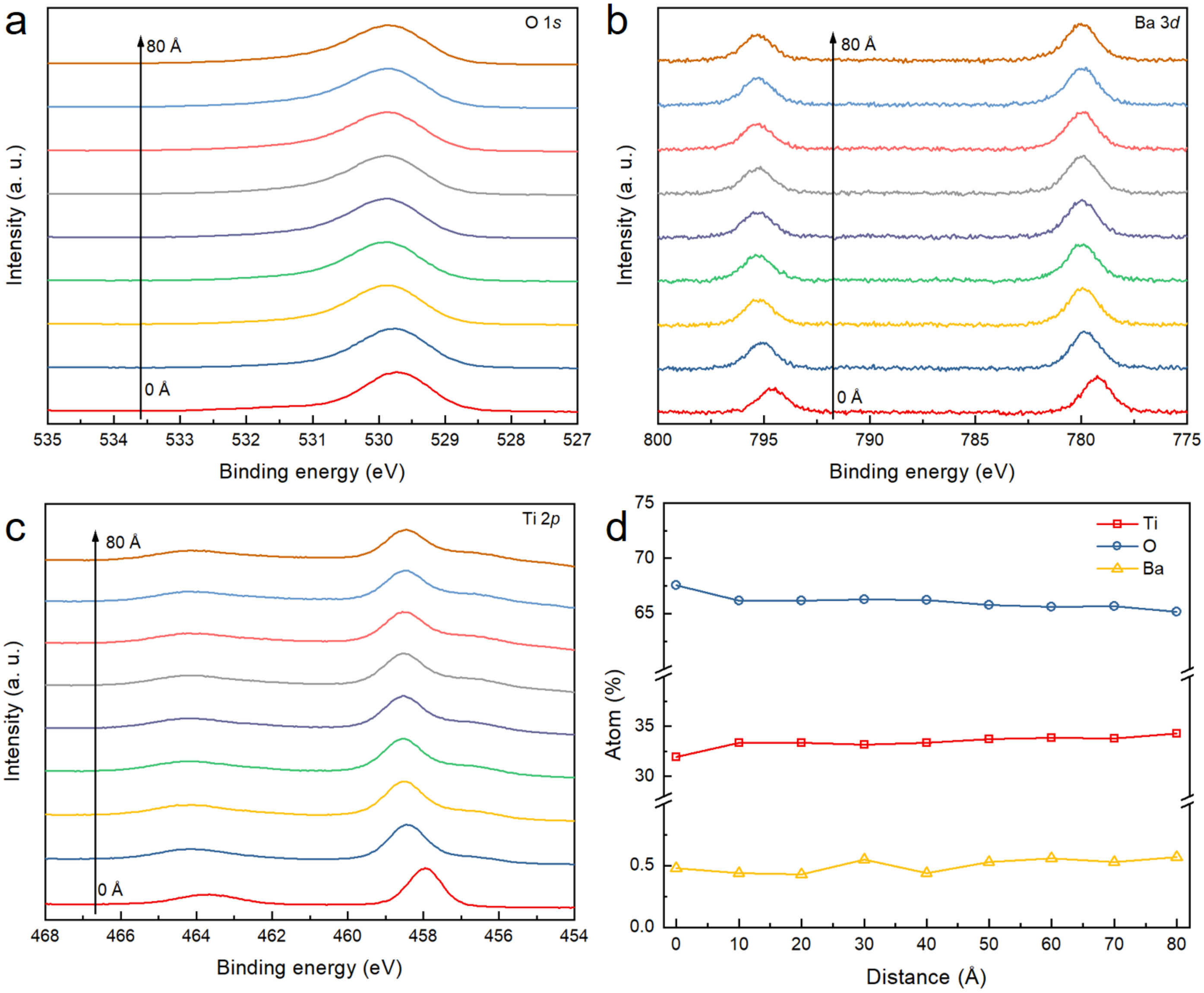

Supplementary Fig. 10 X-ray photoelectron spectroscopy (XPS) depth profiling analysis of Ba:TiO_2. (a) O 1*s*, (b) Ba 3*d*, (c) Ti 2*p* and (d) elements distribution from 0 to 8 nm. These arrows indicate the direction in which the XPS detection depth value increases.

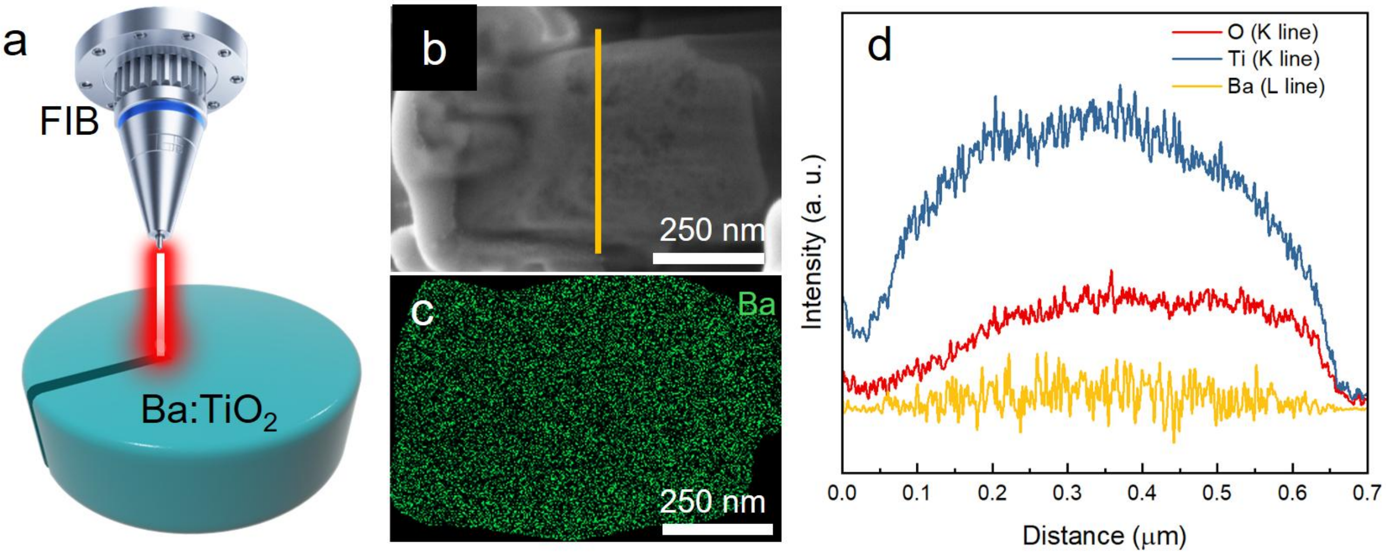

Supplementary Fig. 11 Focused ion beam-scanning electron microscopy (FIB-SEM) cross-sectional elemental distribution analysis. (a) Schematic diagram of FIB-SEM, (b) cross-sectional SEM image, (c) cross-sectional EDS image and (d) line scan EDS element distribution (yellow line in Supplementary Fig. 11b).

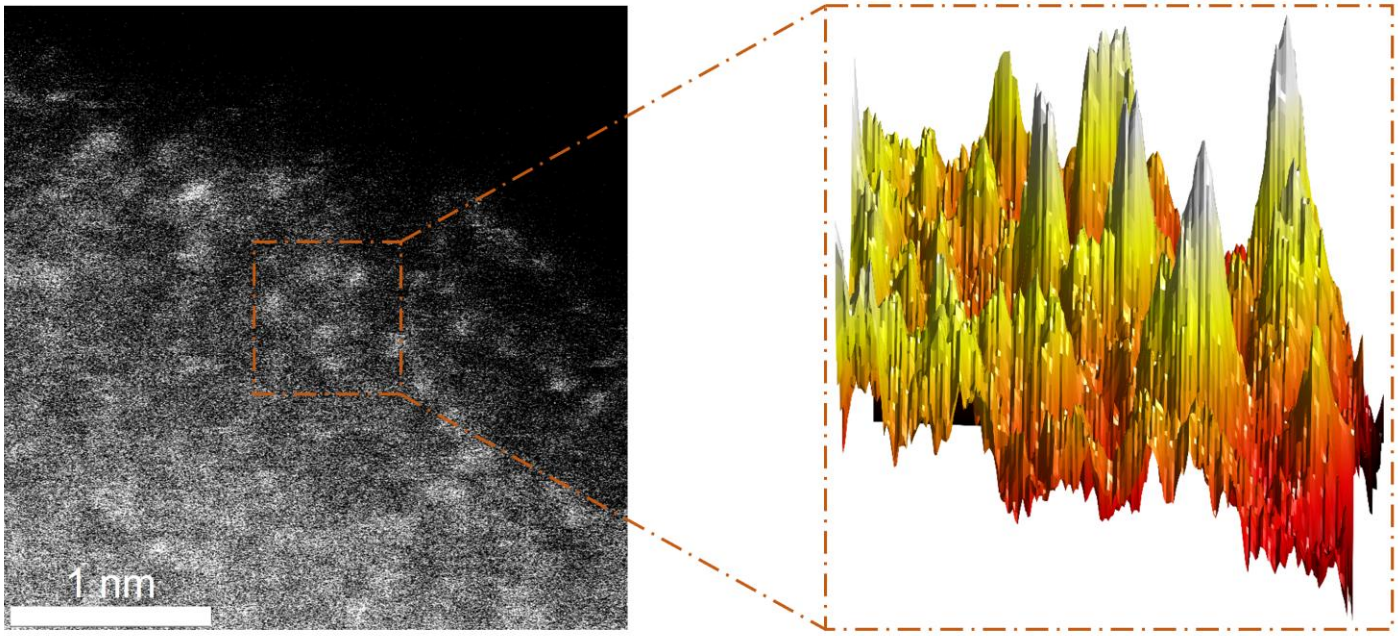

Supplementary Fig. 12 Aberration-corrected scanning transmission electron microscopy (AC-STEM) image of Ba:TiO_2, inset: 3D color map of the cyan-outline region, the protruding tip being Ba single atoms.

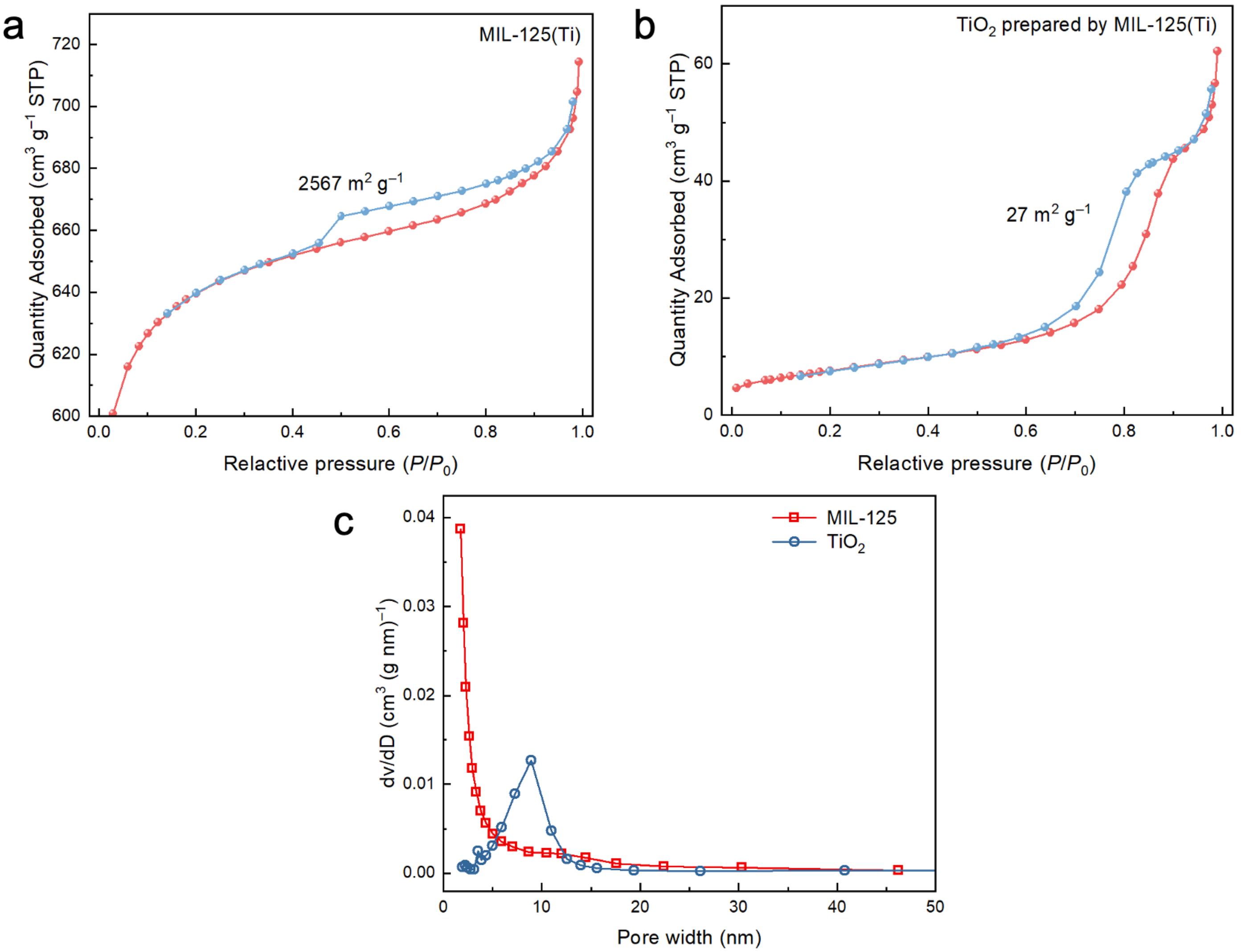

Supplementary Fig. 13 N_2 adsorption isotherms of (a) MIL-125(Ti) and (b) TiO_2, and (c) pore size distribution of corresponding catalysts.

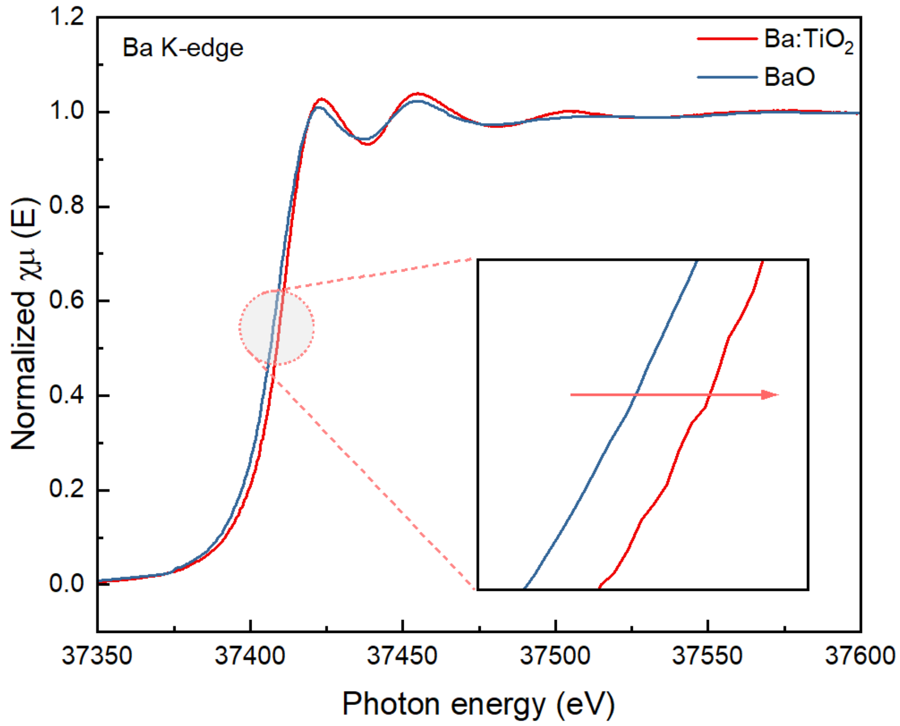

Supplementary Fig. 14 X-ray absorption near edge structure (XANES) spectra of Ba K-edge for Ba:TiO_2 and BaO.

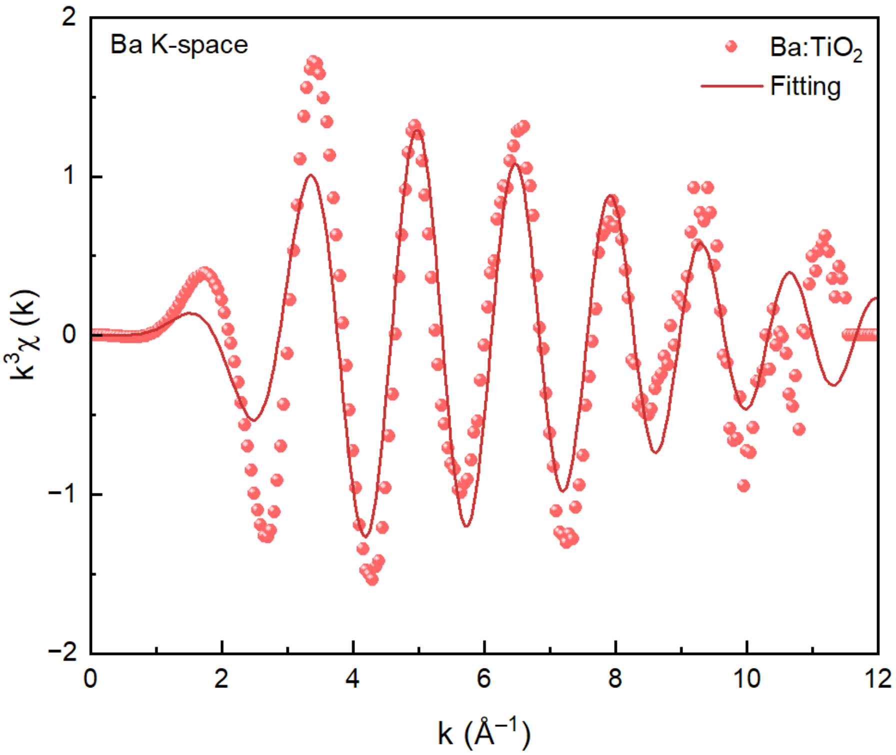

Supplementary Fig. 15 Ba K-edge extended X-ray absorption fine structure (EXAFS, points) and fitting curve (line) for Ba:TiO_2, shown in k^3-weighted K-space.

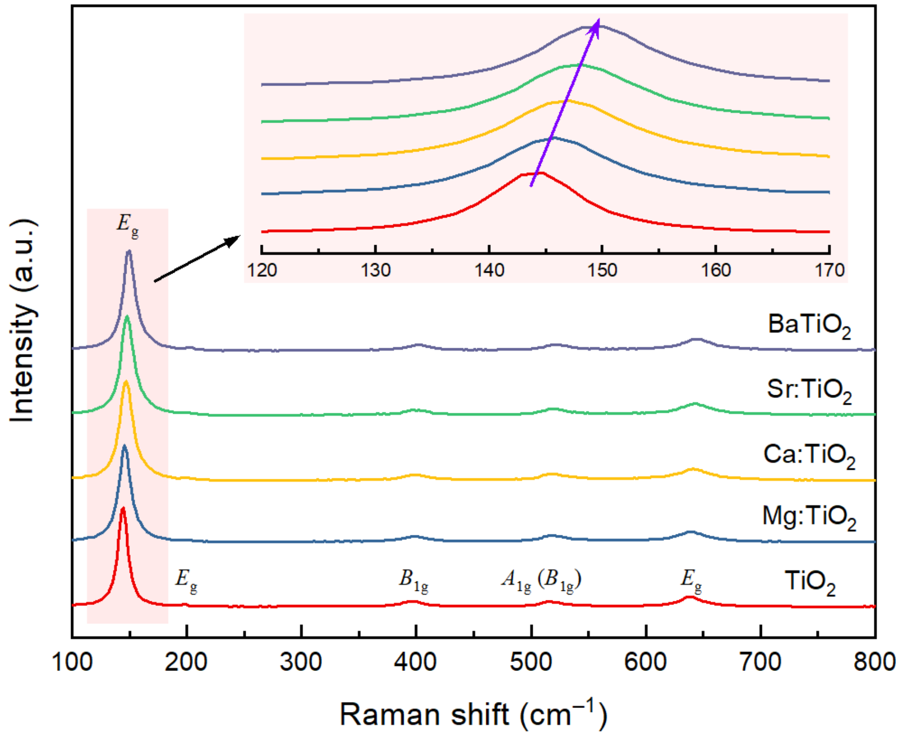

Supplementary Fig. 16 Raman spectra of TiO_2 samples, laser wavelength: 532 nm, laser intensity: 1%, exposure time: 10 s. These arrows represent the direction of peak shift.

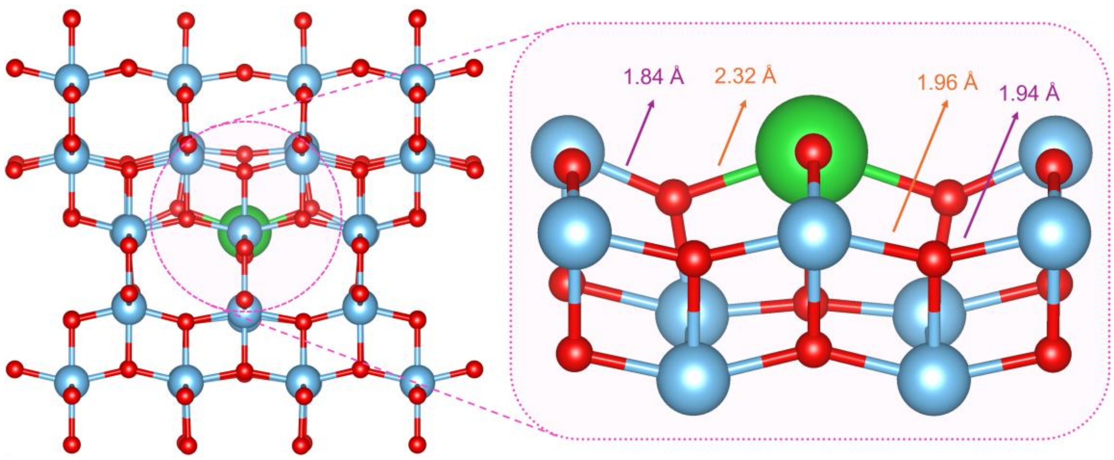

Supplementary Fig. 17 Optimized structures of bulk Ba:TiO_2. The red ball, light blue ball and green ball represent O, Ti, and Ba atom, respectively. The images generated by VESTA visualization software [1]. The values pointed to by these arrows represent the lengths of corresponding chemical bonds.

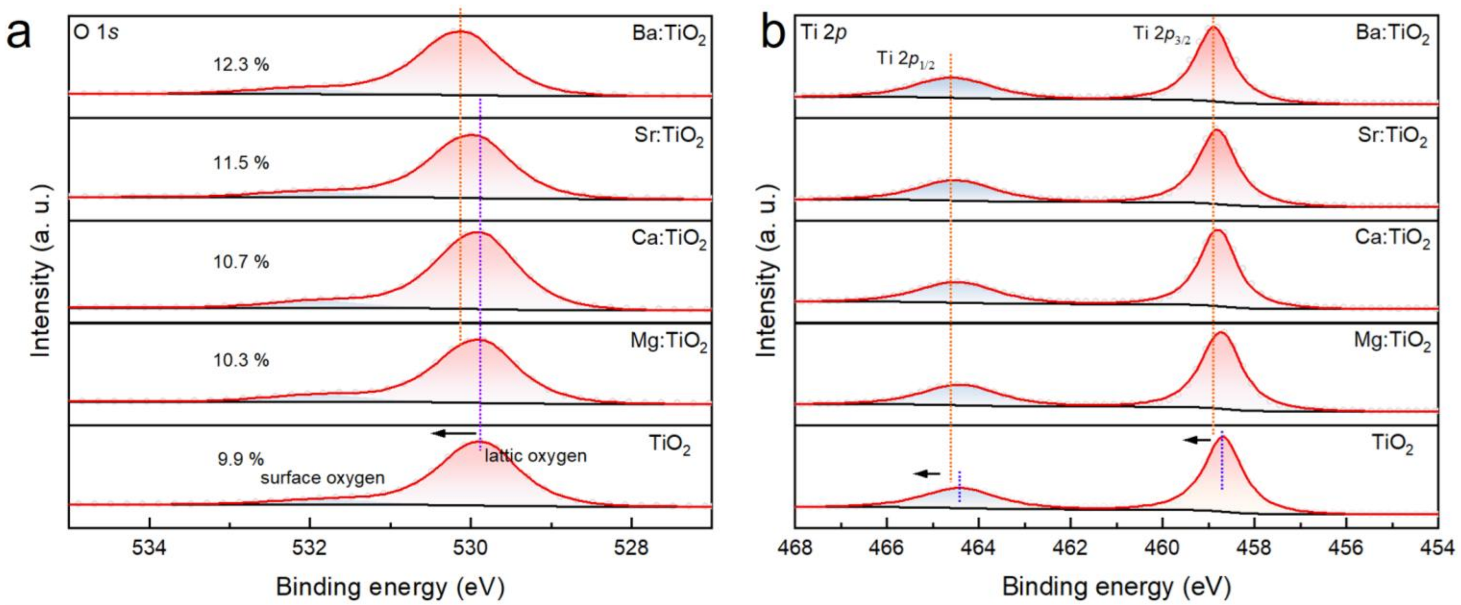

Supplementary Fig. 18 High-resolution X-ray photoelectron spectroscopy (XPS) spectra of (a) O 1*s* and (b) Ti 2*p* on various TiO_2 samples. These arrows represent the direction of peak shift.

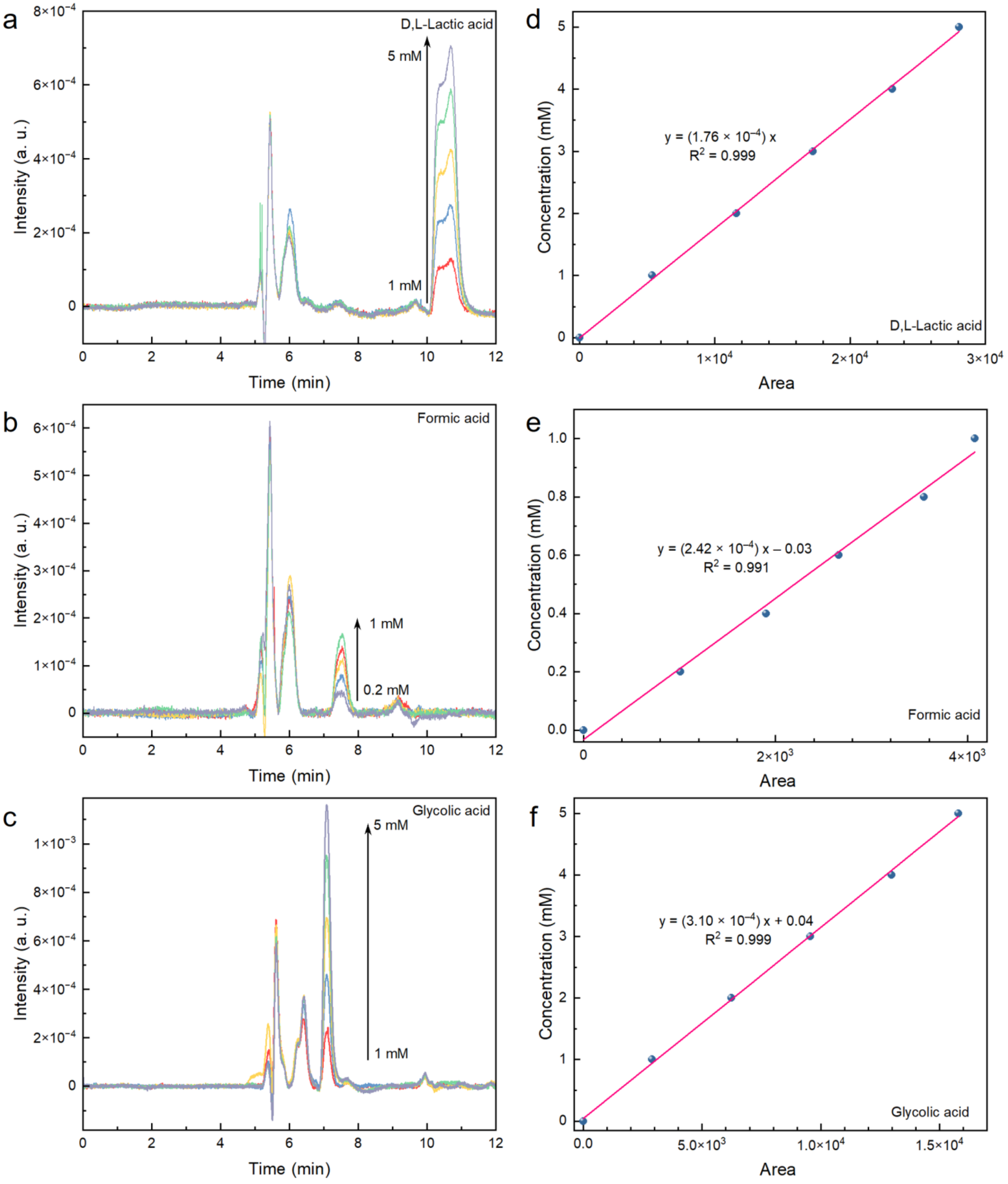

Supplementary Fig. 19 High performance liquid chromatography (HPLC) of standard solutions of (a) D,L-lactic acid, (b) formic acid, and (c) glycolic acid. Corresponding calibration curves of (d) D,L-lactic acid, (e) formic acid, and (f) glycolic acid. The arrows indicate increasing concentrations of the standard solutions.

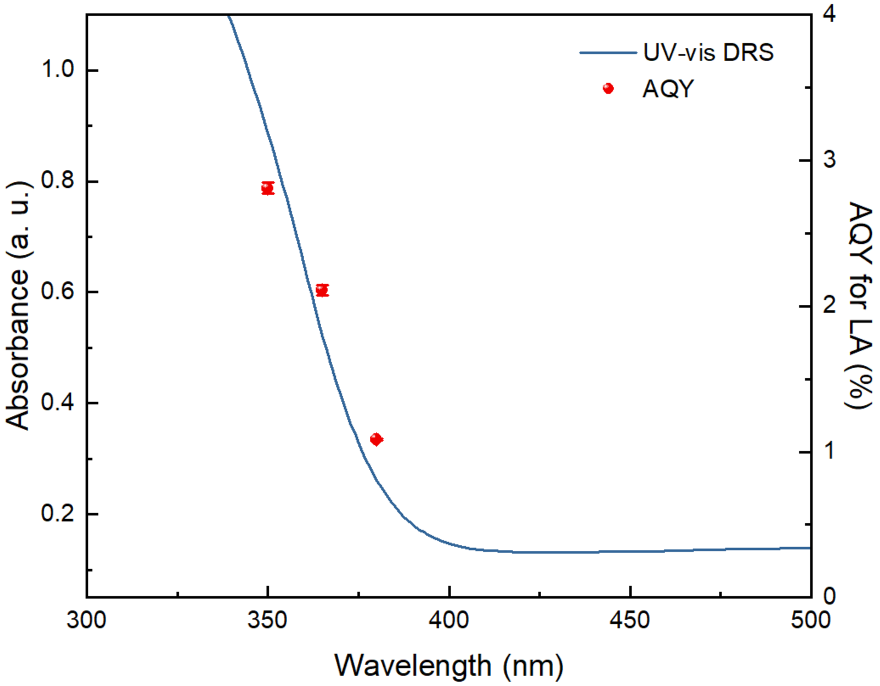

Supplementary Fig. 20 The apparent quantum yield (AQY) of Ba:TiO_2 sample. Reaction conditions: 5 mg of catalysts, 20 mL of reaction mixture (50 vol% methanol, ethylene glycol with molar ratio to methanol 1:5, and KOH with concentration is 4.0 M), T = 25 °C, 1 bar Ar, t = 2 h. Light intensity: 22.9 mW cm^{-2} (350 nm); 26.5 mW cm^{-2} (365 nm); 28.5 mW cm^{-2} (380 nm). Irradiated area: 9.6 cm^2. A minimum of three replicate measurements was performed for each material group to ensure reproducibility.

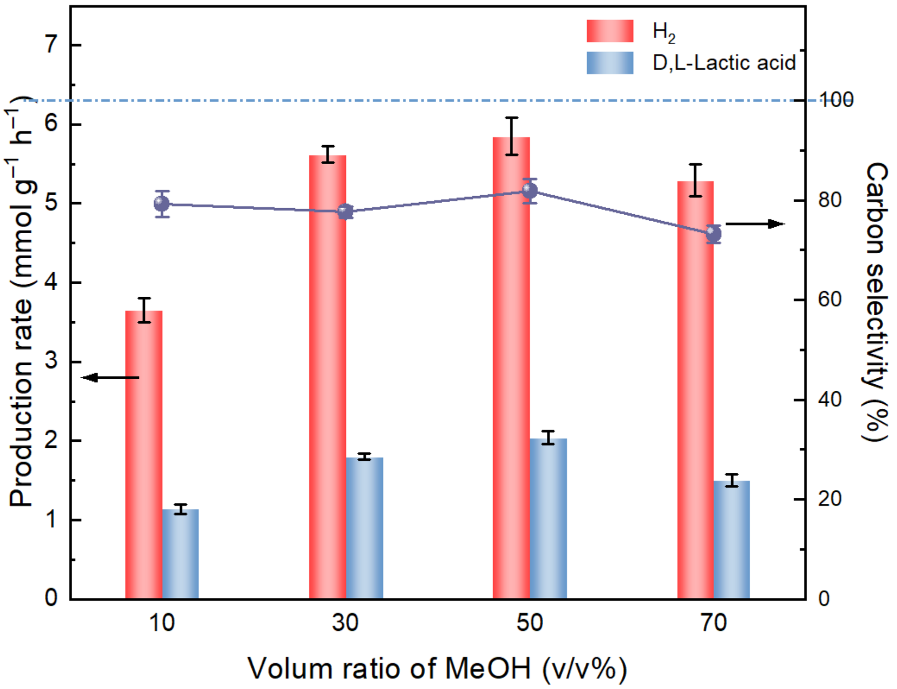

Supplementary Fig. 21 D,L-lactic acid (LA) and H_2 production rate over different amount of methanol (MeOH) addition in the reaction system; the bar chart uses the left vertical axis, while the point plot uses the right vertical axis, as indicated by these arrows. Reaction conditions: 5 mg of catalysts, 20 mL of reaction mixture (ethylene glycol with molar ratio to MeOH 1:5, and KOH with concentration is 4.0 M), T = 25 °C, 1 bar Ar, t = 2 h. A minimum of three replicate measurements was performed for each material group to ensure reproducibility.

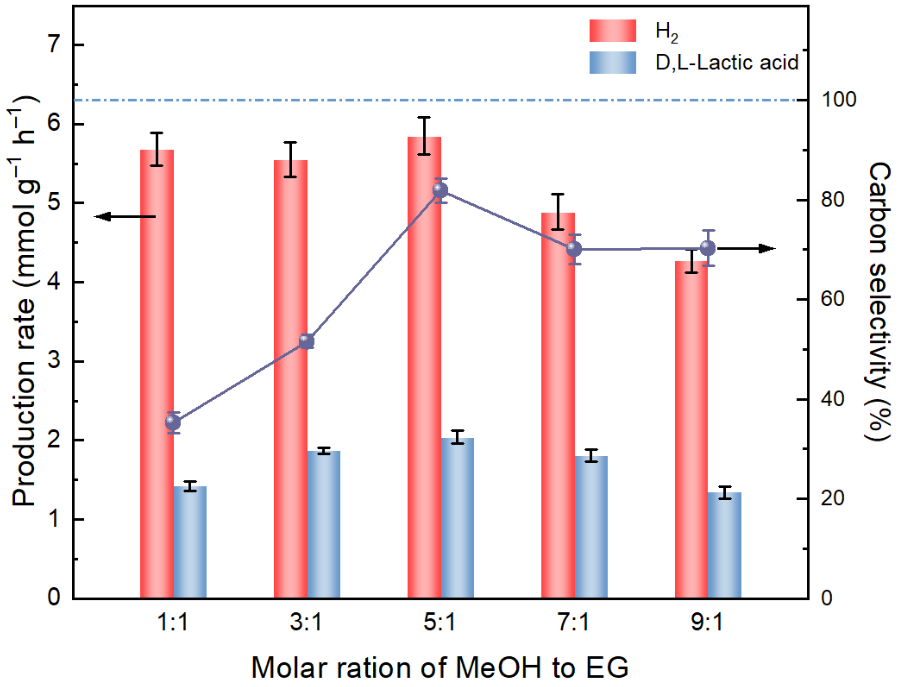

Supplementary Fig. 22 D,L-lactic acid (LA) and H_2 production rate over different molar ration of methanol (MeOH) to ethylene glycol (EG) in the reaction system; the bar chart uses the left vertical axis, while the point plot uses the right vertical axis, as indicated by these arrows. Reaction conditions: 5 mg of catalysts, 20 mL of reaction mixture (50 v/v% of MeOH, and KOH with concentration is 4.0 M), T = 25 °C, 1 bar Ar, *t* = 2 h. A minimum of three replicate measurements was performed for each material group to ensure reproducibility.

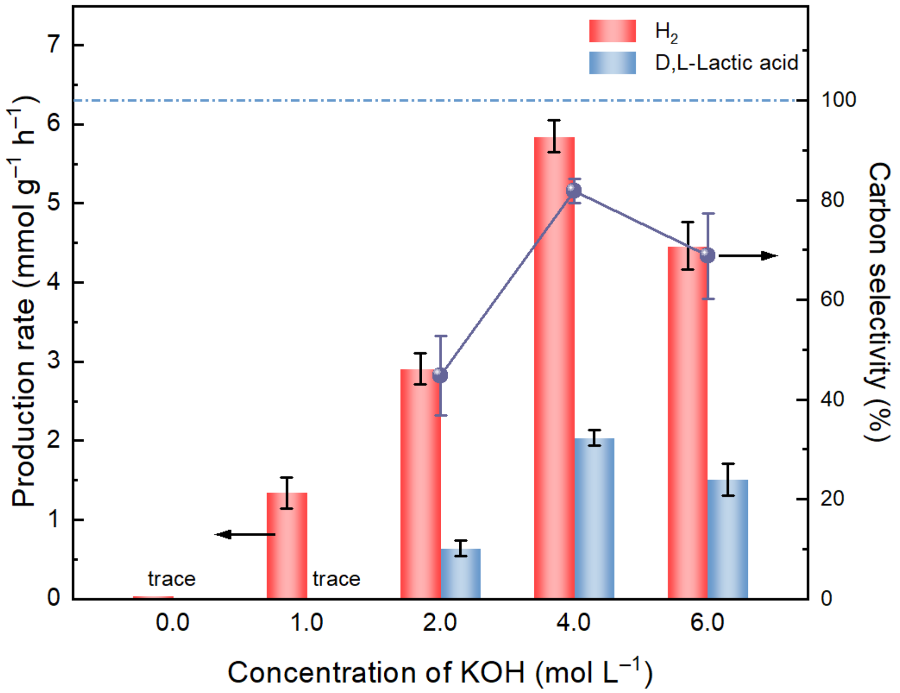

Supplementary Fig. 23 D,L-lactic acid (LA) and H_2 production rate over different concentration of KOH in the reaction system; the bar chart uses the left vertical axis, while the point plot uses the right vertical axis, as indicated by these arrows. Reaction conditions: 5 mg of catalysts, 20 mL of reaction mixture (50 vol% methanol, and ethylene glycol with molar ratio to methanol 1:5), T = 25 °C, 1 bar Ar, *t* = 2 h. A minimum of three replicate measurements was performed for each material group to ensure reproducibility.

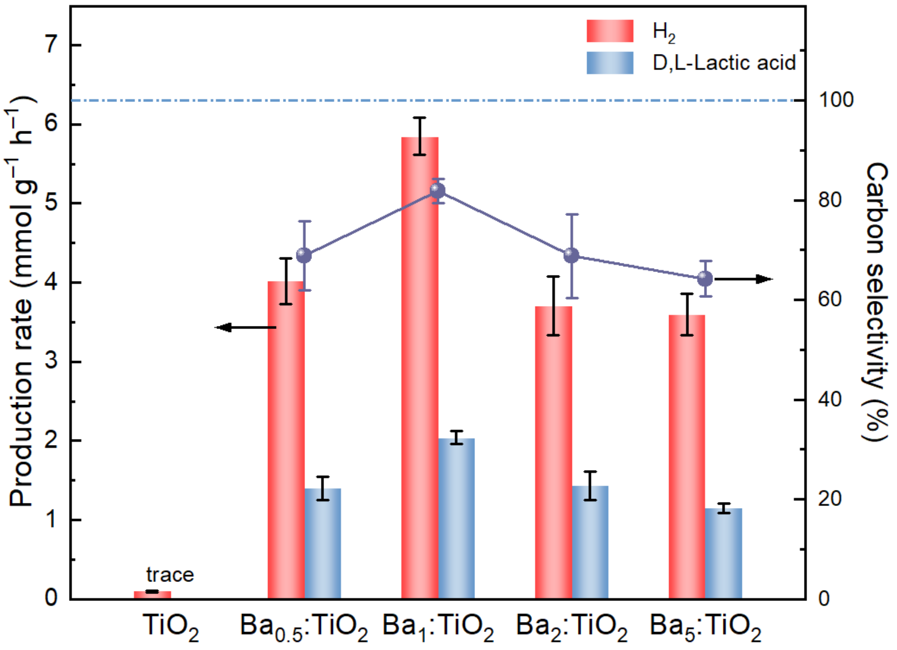

Supplementary Fig. 24 D,L-lactic acid (LA) and H_2 production rate over different incorporation amount of Ba in TiO_2; the bar chart uses the left vertical axis, while the point plot uses the right vertical axis, as indicated by these arrows. Reaction conditions: 5 mg of catalysts, 20 mL of reaction mixture (50 vol% methanol, ethylene glycol with molar ratio to methanol 1:5, and KOH with concentration is 4.0 M), T = 25 °C, 1 bar Ar, *t* = 2 h. A minimum of three replicate measurements was performed for each material group to ensure reproducibility.

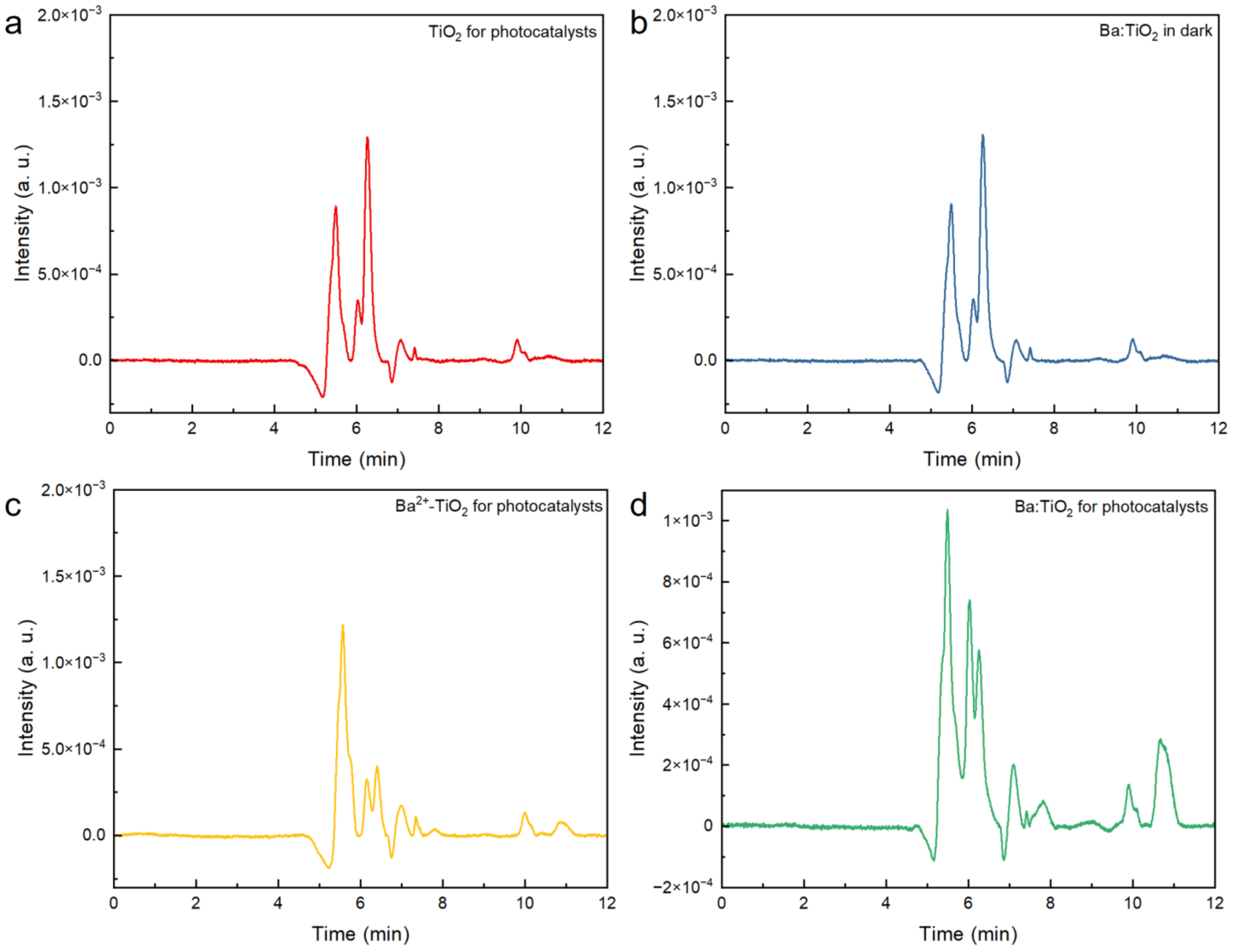

Supplementary Fig. 25 High performance liquid chromatography (HPLC) spectra of (a) TiO_2, (b) Ba:TiO_2 in dark, (c) Ba^{2+}-TiO_2, and (d) Ba:TiO_2, corresponding to Fig. 4b. Minor invariant peaks observed at specific retention times are attributed to system-related background signals or trance impurities are not associated with photocatalytic reaction products.

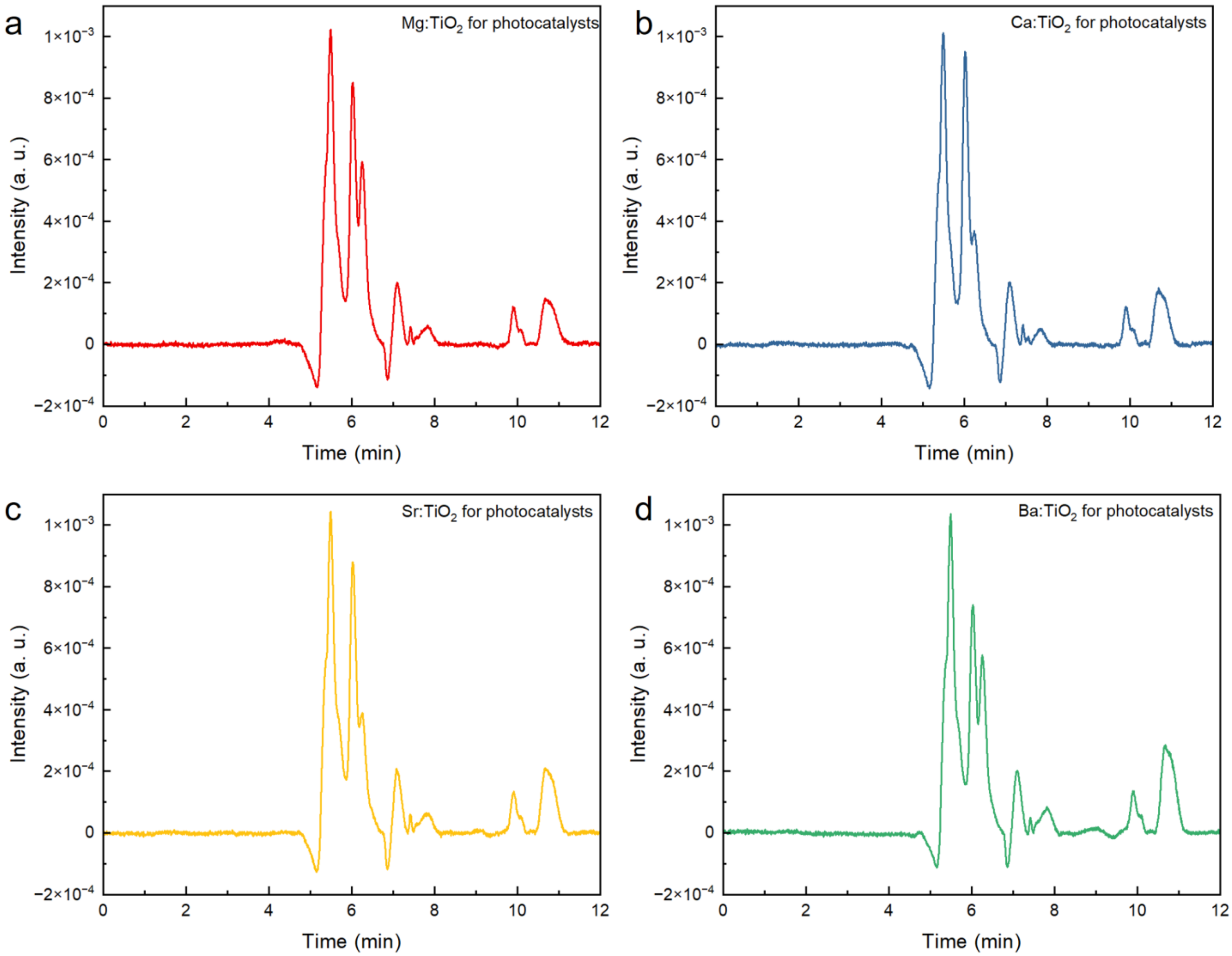

Supplementary Fig. 26 High performance liquid chromatography (HPLC) spectra of (a) Mg:TiO_2, (b) Ca:TiO_2, (c) Sr:TiO_2, and (d) Ba:TiO_2, corresponding to Fig. 4c. Minor invariant peaks observed at specific retention times are attributed to system-related background signals or trance impurities are not associated with photocatalytic reaction products.

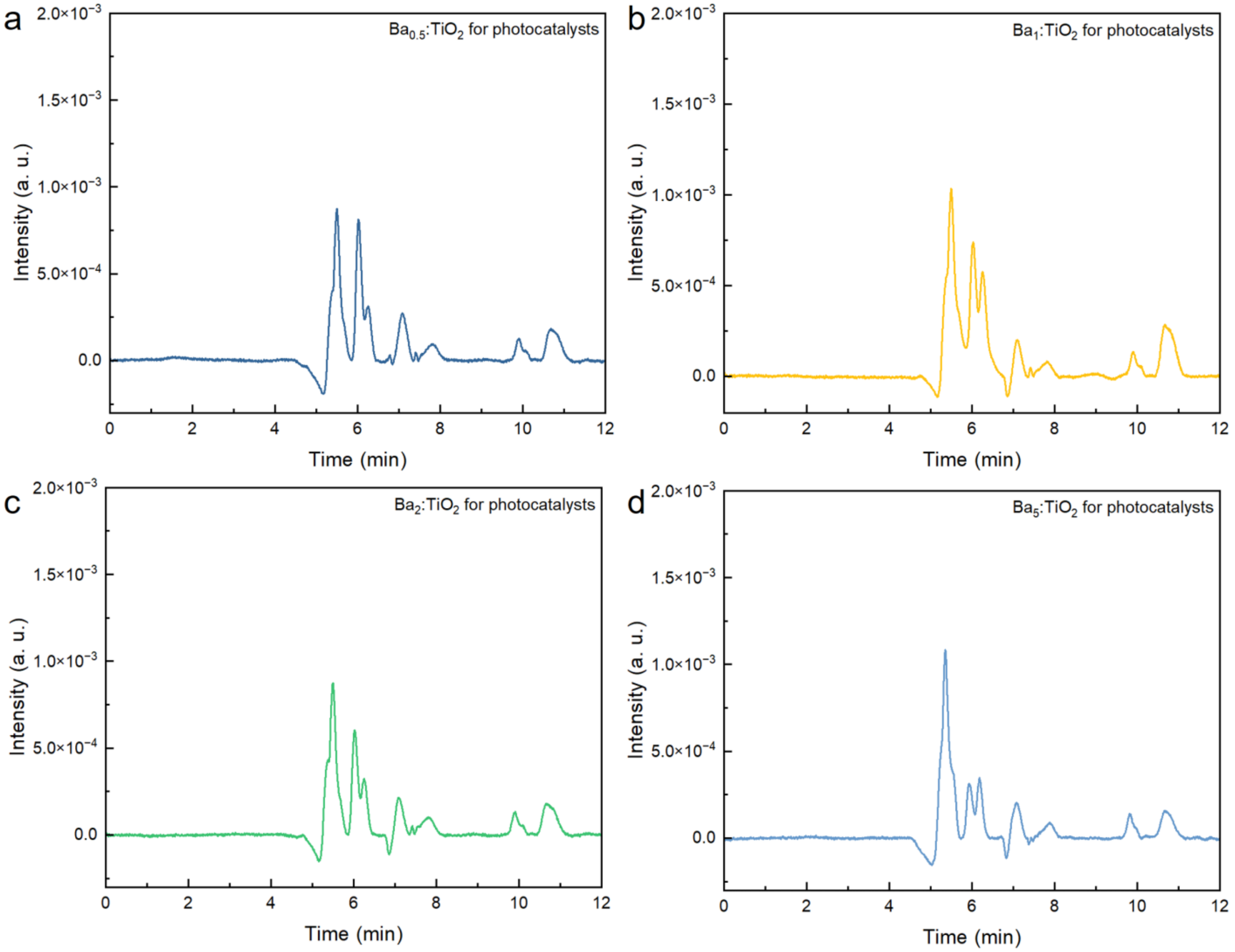

Supplementary Fig. 27 High performance liquid chromatography (HPLC) spectra of (a)$Ba_{0.5}:TiO_2$, (b) $Ba_1:TiO_2$, (c) $Ba_2:TiO_2$, and (d) $Ba_5:TiO_2$, corresponding to Supplementary Fig. 24. Minor invariant peaks observed at specific retention times are attributed to system-related background signals or trance impurities are not associated with photocatalytic reaction products.

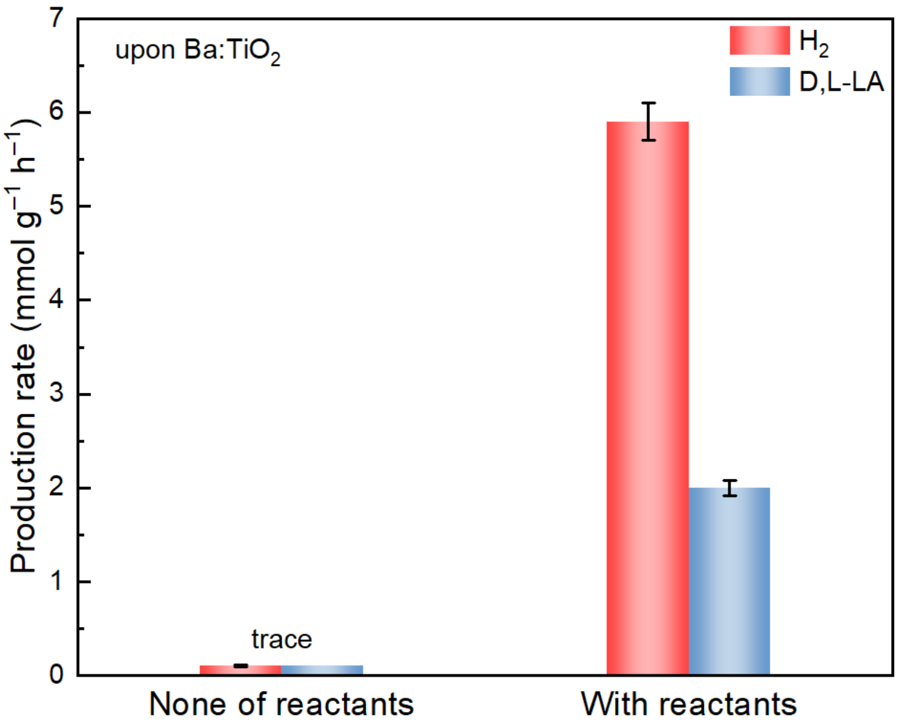

Supplementary Fig. 28 D,L-lactic acid (LA) and H_2 production rate on Ba:TiO_2 without or with ethylene glycol and methanol. Reaction conditions: 5 mg of catalysts, 20 mL of H_2O or reaction mixture (50 vol% methanol, ethylene glycol with molar ratio to methanol 1:5, and KOH with concentration is 4.0 M), T = 25 °C, 1 bar Ar, *t* = 2 h. A minimum of three replicate measurements was performed for each material group to ensure reproducibility.

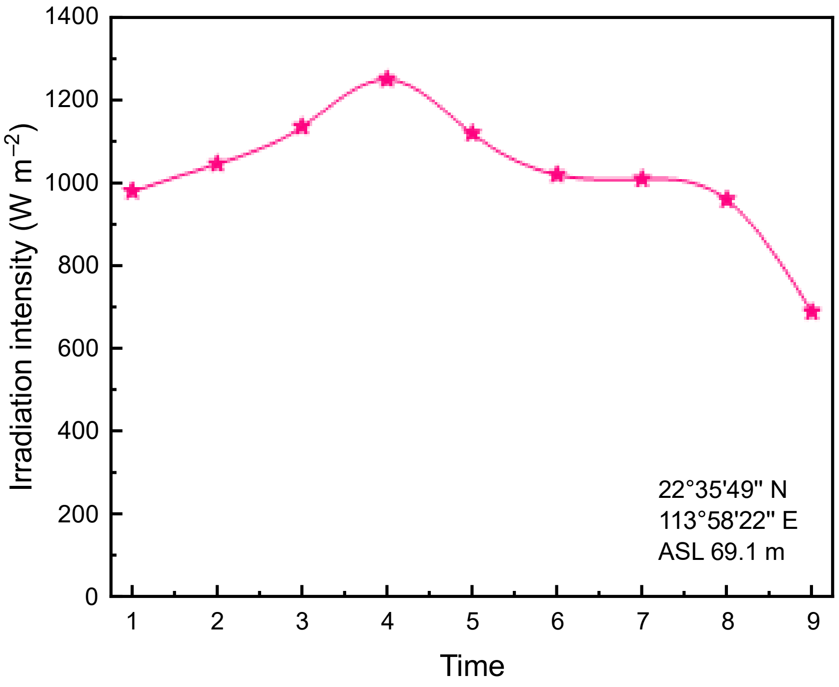

Supplementary Fig. 29 Outdoor solar irradiation conditions, collected at 22°35’49” N, 113°58’22” E, and on Aug 10, 2025.

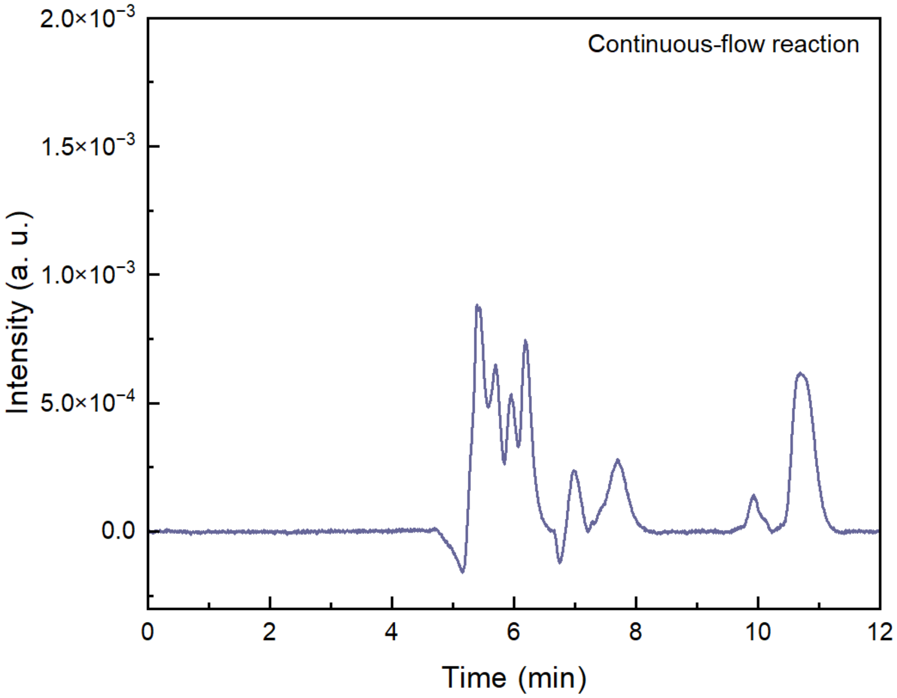

Supplementary Fig. 30 Continuous-flow reaction under a full day of outdoor sunlight exposure (from 9:00 to 17:00), collected at 22°35’49’’ N, 113°58’22’’ E, and on Aug 10, 2025.

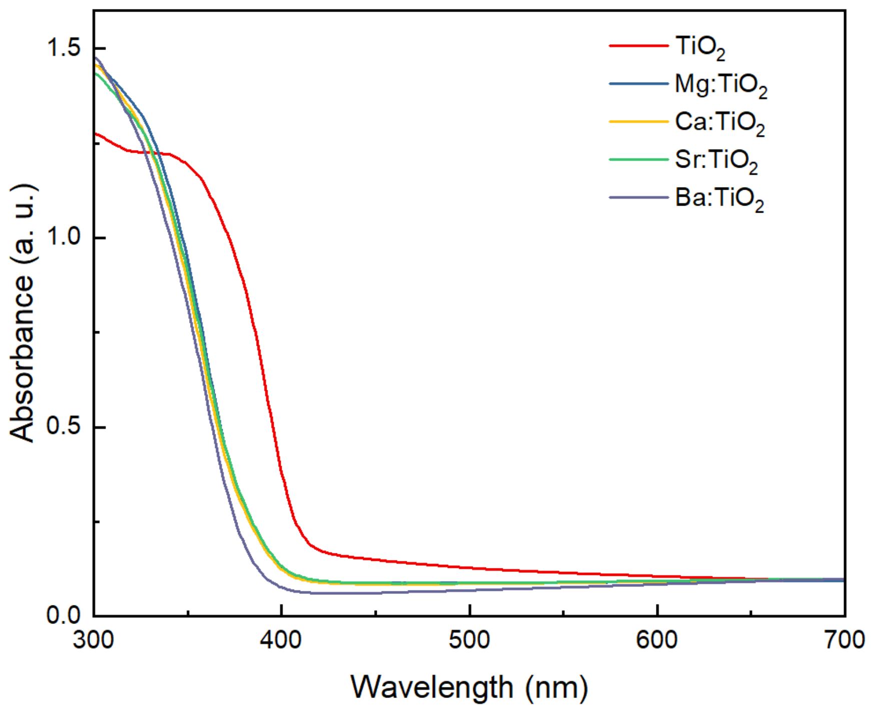

Supplementary Fig. 31 Ultraviolet-visible diffuse reflectance spectroscopy (UV–vis DRS) spectra of various TiO_2 samples.

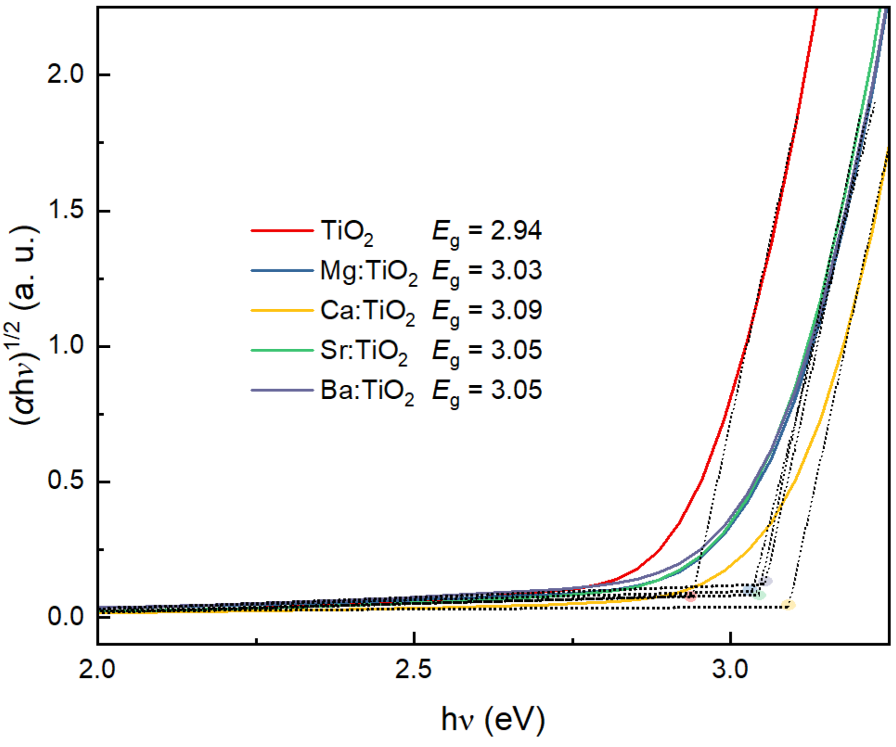

Supplementary Fig. 32 Tauc plots of various TiO_2 samples.

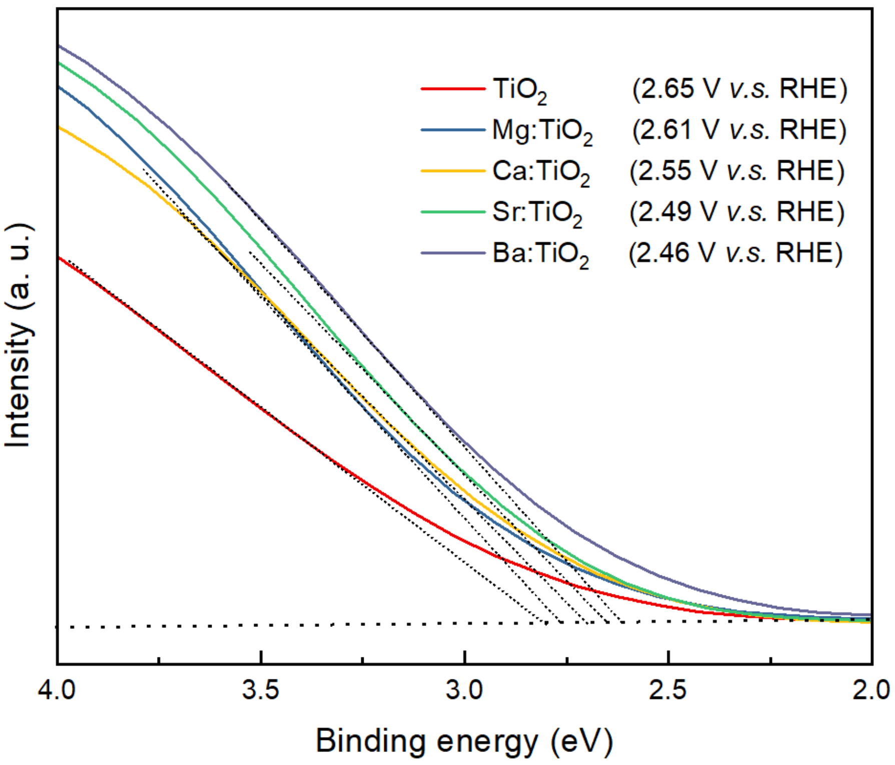

Supplementary Fig. 33 Valence band-XPS (VB-XPS) spectra of various TiO_2 samples.

NOTES: The measurement error was eliminated by the following formula with the work function (Φ) of XPS analyzer and vacuum level to be 3.88 and 4.44 eV (vs. NHE), the VB of TiO_2 and M:TiO_2 were calculated to be 2.65 (TiO_2), 2.61 (Mg:TiO_2), 2.55 (Ca:TiO_2), 2.49 (Sr:TiO_2), and 2.46 V vs. RHE (Ba:TiO_2) [2].

$$VB\ (vs.\ RHE) = \Phi + VB\text{-}XPS - 4.44 + 0.059\ pH \quad (1)$$

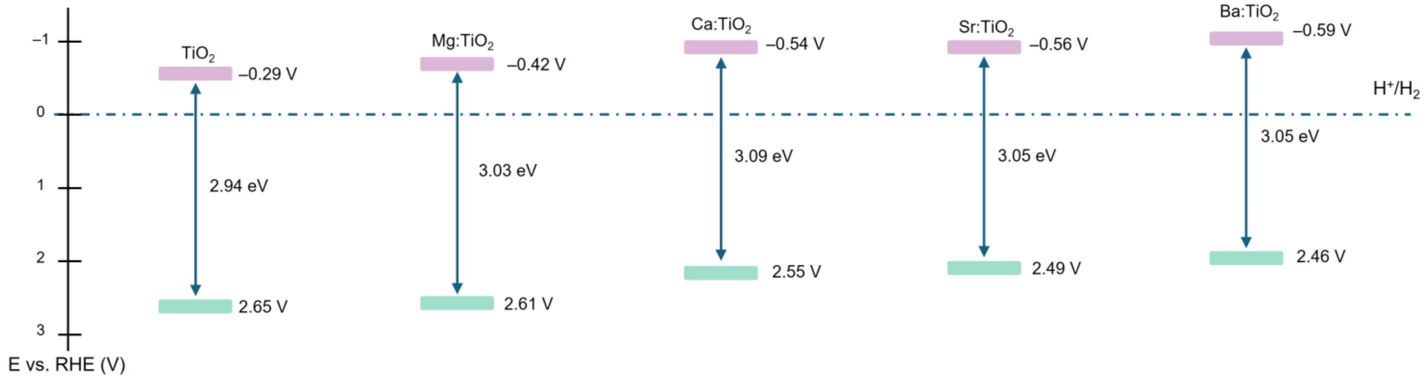

Supplementary Fig. 34 Band structure of TiO_2 and M:TiO_2 calculated by valence band-XPS and band gap, which potential (E) vs. reversible hydrogen electrode (RHE).

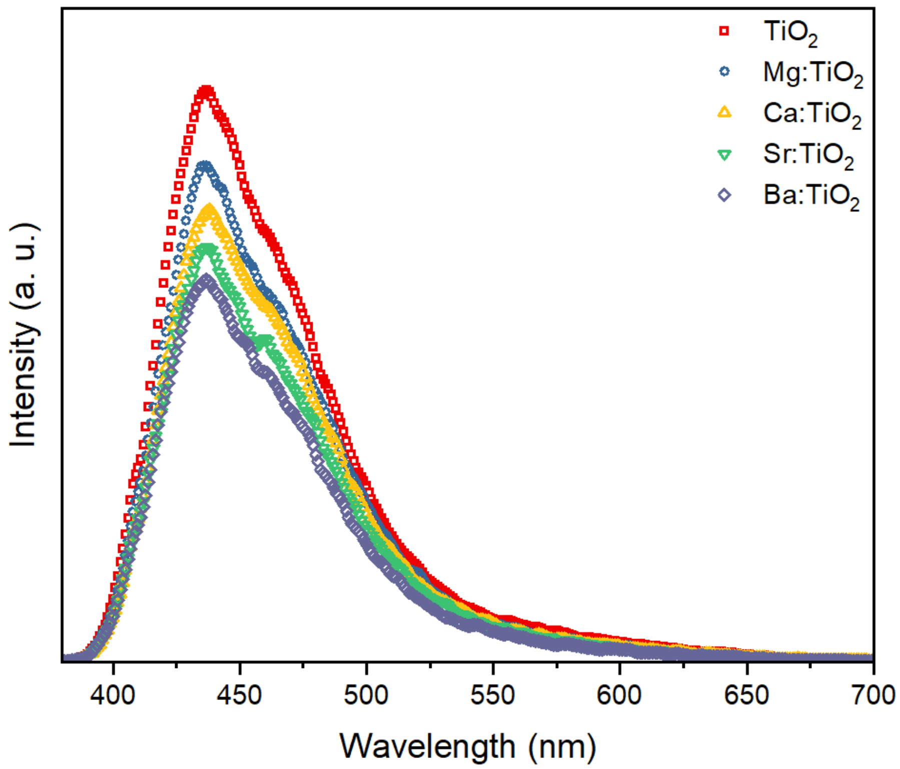

Supplementary Fig. 35 Steady state photoluminescence (PL) spectra of TiO_2 and M:TiO_2 samples (λ_{ex}: 350 nm).

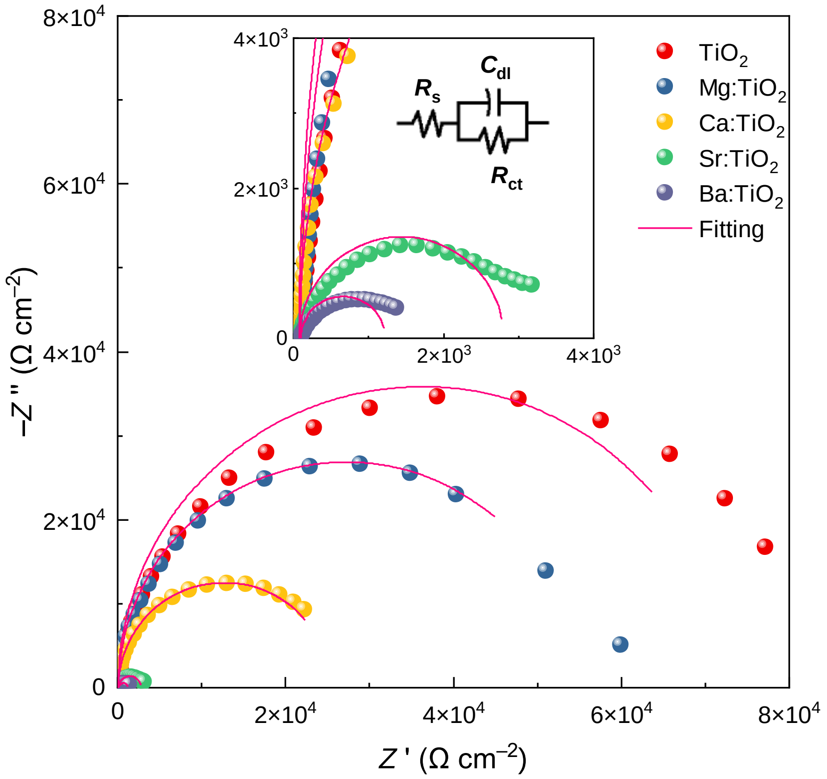

Supplementary Fig. 36 Electrochemical impedance spectroscopy (EIS) plots of TiO_2 samples in Nyquist formats with corresponding fitting data collected over a period of 30 min (after 10 min open-circuit potential stabilization) over a range of frequency from 100 kHz to 10 mHz. Inset: enlarged view of Nyquist plots with Z' range of 0–4 × 10^3 Ω cm^{-2}, and equivalent circuit diagram for fitting Nyquist plots (R_s: the solution resistance, C_{dl}: double-layer capacitance; R_{ct}: and charge transfer resistance).

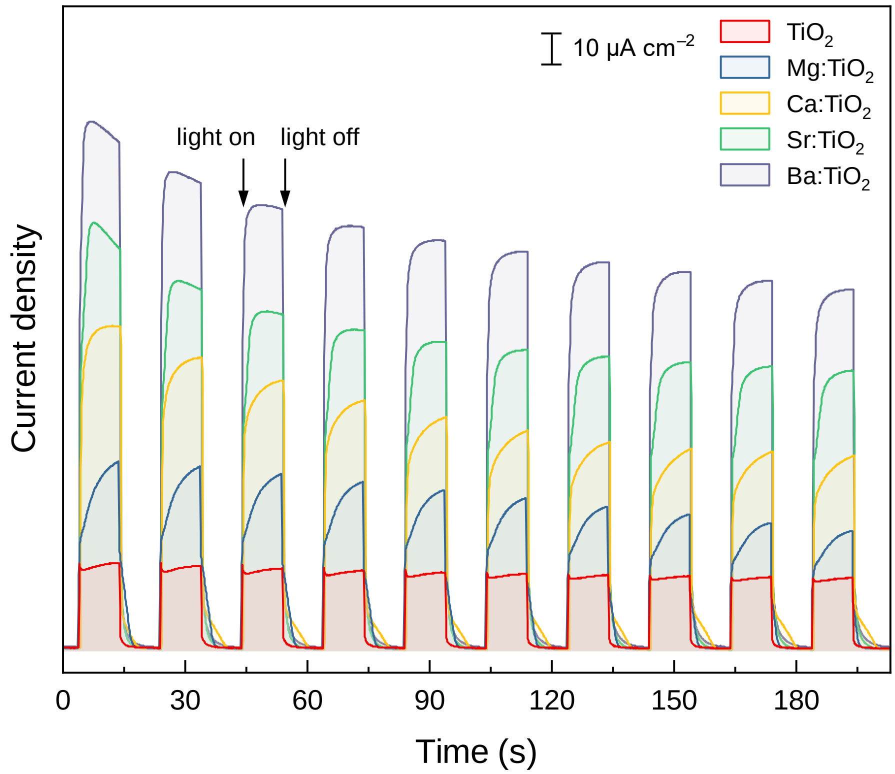

Supplementary Fig. 37 Transient photocurrent density-time profiles of TiO_2 samples at 1 V (vs. RHE) in 0.2 M Na_2SO_2 under 100 W LED lamp illumination (365 nm), the measurement without iR-correction.

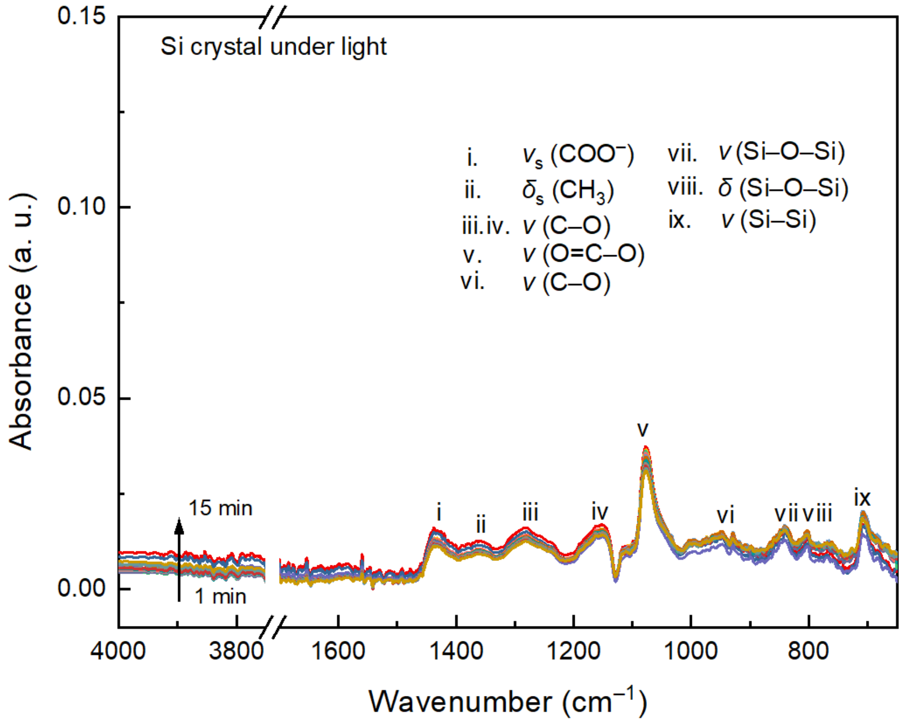

Supplementary Fig. 38 In-situ attenuated total reflection Fourier transform infrared spectroscopy (ATR-FTIR) spectra over neat ATR crystal, reaction conditions: 5 mg of photocatalysts, 50 vol% MEOH, molar ratio of MeOH to EG is 5:1, concentration of KOH is 4.0 M, T = 25 °C, 1 bar Ar, and Xe lamp irradiation.

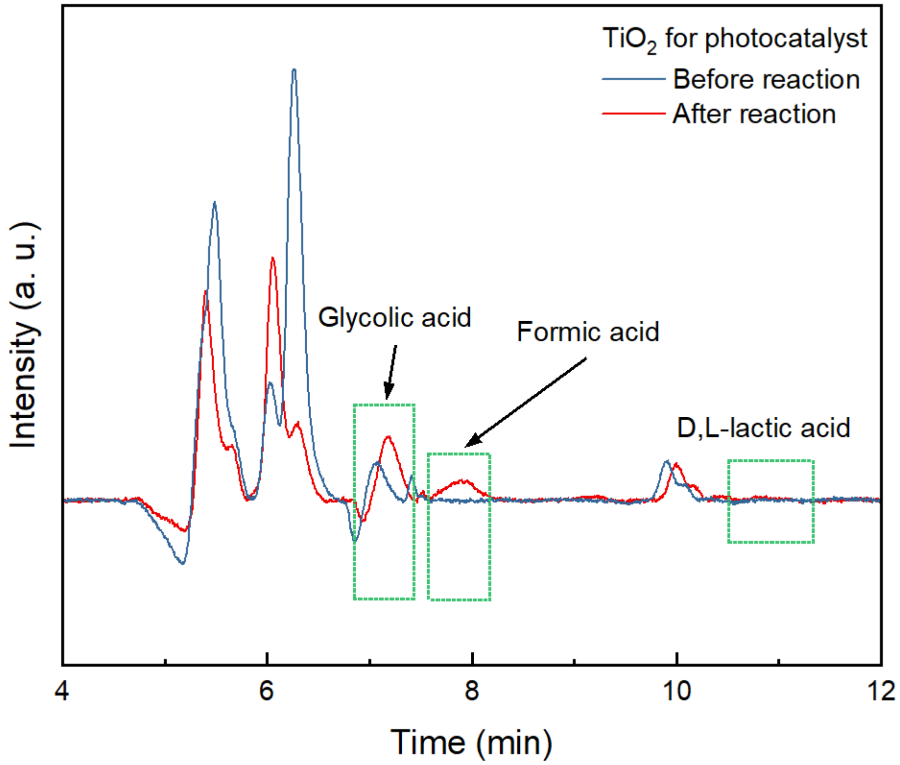

Supplementary Fig. 39 High performance liquid chromatography (HPLC) results of the photocatalytic C–C cross-coupling to produce LA system using TiO_2 as a catalyst after a 2-h reaction.

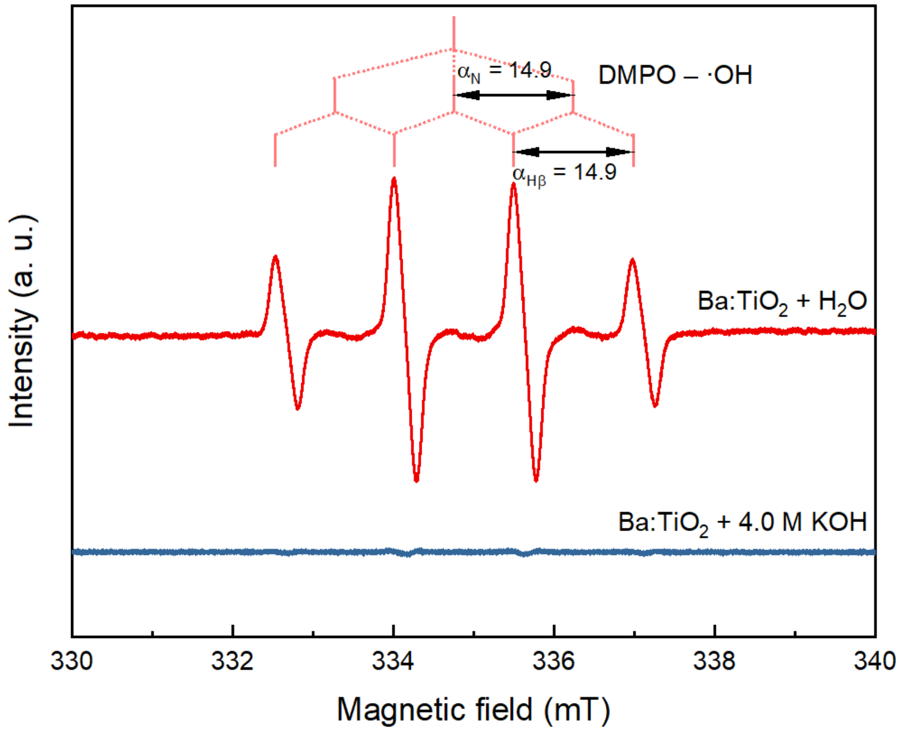

Supplementary Fig. 40 5,5 dimethyl-1-pyrroline N-oxide (DMPO)-trapping in-situ electron spin resonance (ESR) experiments.

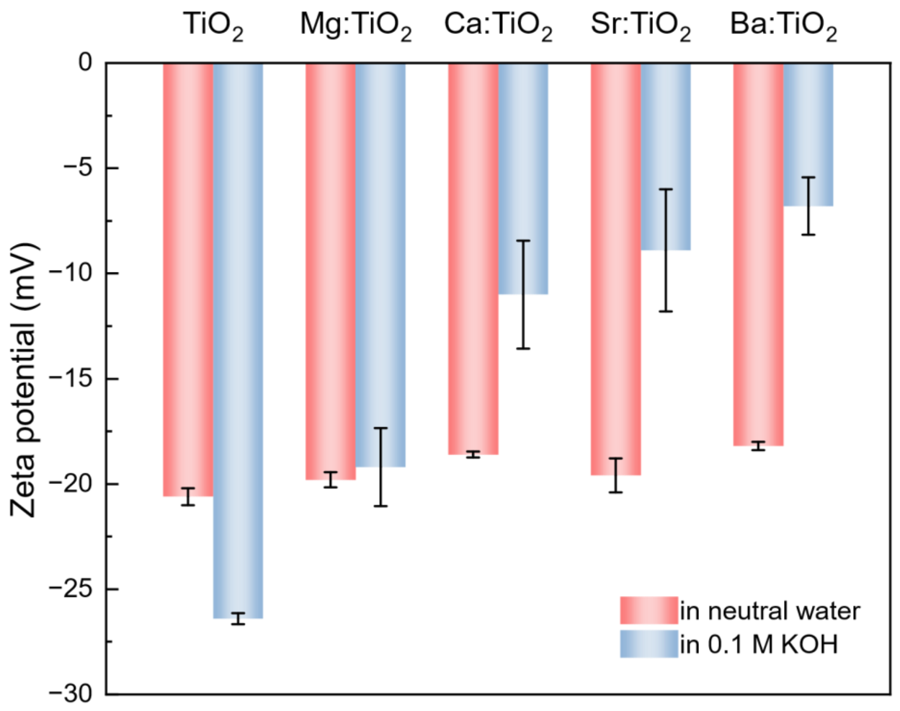

Supplementary Fig. 41 Zeta potential of pristine TiO_2 and M:TiO_2 in neutral water (pH 6.8 ± 0.08) and 0.1 M KOH, ($n \geq 3$).

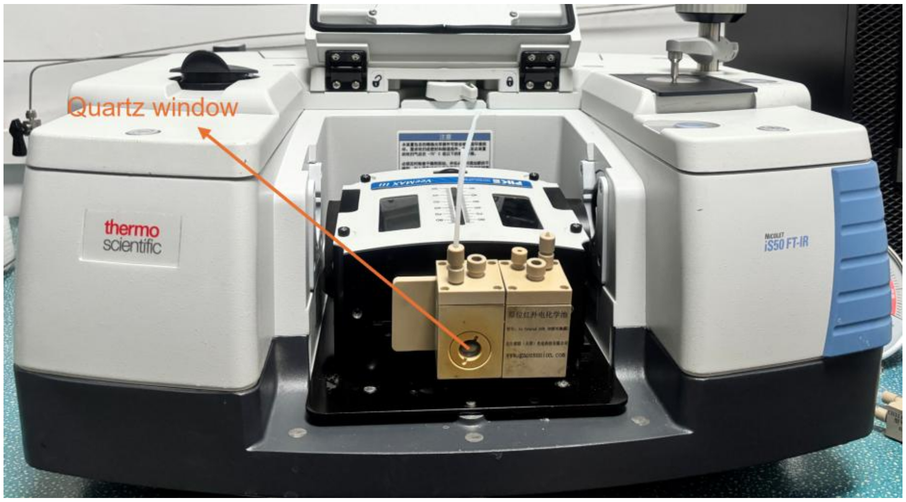

Supplementary Fig. 42 Image of custom-built photocatalytic reactor for in situ attenuated total reflection Fourier transform infrared spectroscopy studies.

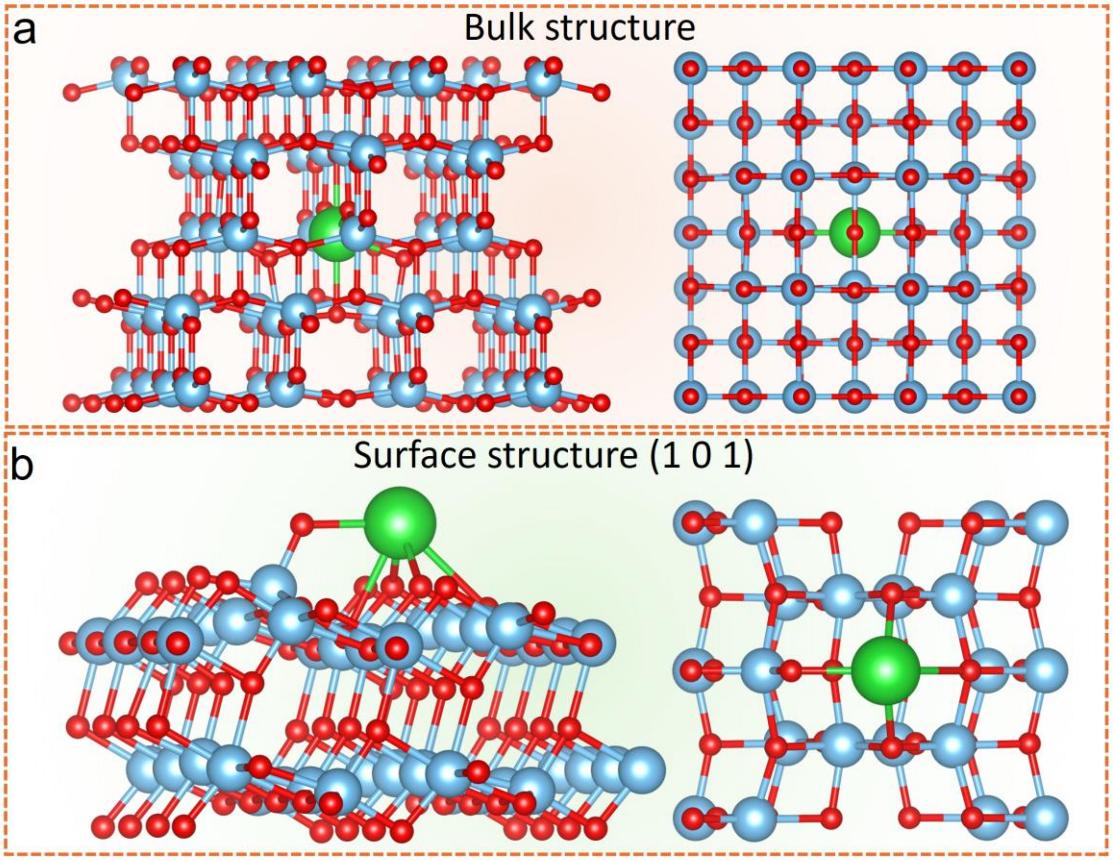

Supplementary Fig. 43 Optimized structures of (a) bulk and (b) surface incorporation of Ba upon TiO_2. The red balls, light blue balls and green balls represent O, Ti, and Ba atom, respectively. The images generated by VESTA visualization software [1].

Supplementary Table 1. Load amount of alkaline earth metals elements in TiO_2

	**$Mg_1:TiO_2$**	**$Ca_1:TiO_2$**	**$Sr_1:TiO_2$**	**$Ba_{0.5}:TiO_2$**	**$Ba_1:TiO_2$**	**$Ba_2:TiO_2$**	**$Ba_5:TiO_2$**
wt. %	1.04 ± 0.01	0.98 ± 0.01	0.77 ± 0.01	0.36 ± 0.02	0.92 ± 0.01	1.83 ± 0.02	4.66 ± 0.06

Supplementary Table 2. Production rates of TiO_2 and $M:TiO_2$. Reaction conditions: 5 mg of catalysts, 20 mL of reaction mixture (ethylene glycol with molar ratio to CH_3OH 1:5, and KOH with concentration is 4.0 M), T = 25 °C, 1 bar Ar, *t* = 2 h. A minimum of three replicate measurements was performed for each material group to ensure reproducibility.

	**TiO_2**	**$Mg:TiO_2$**	**$Ca:TiO_2$**	**$Sr:TiO_2$**	**$Ba:TiO_2$**
D,L-lactic acid ($mmol\ g^{-1}\ h^{-1}$)	N/A	1.16 ± 0.10	1.35 ± 0.06	1.56 ± 0.10	2.04 ± 0.08
Formic acid ($mmol\ g^{-1}\ h^{-1}$)	0.8 ± 0.07	0.25 ± 0.02	0.29 ± 0.01	0.27 ± 0.02	0.28 ± 0.02
Glycolic acid ($mmol\ g^{-1}\ h^{-1}$)	N/A	0.73 ± 0.01	0.78 ± 0.01	0.89 ± 0.01	0.54 ± 0.01

Supplementary Table 3. Transient state photoluminescence fitting results of various samples

	**τ_1 (ns)**	**B_1 (Rel.%)**	**τ_2 (ns)**	**B_2 (Rel.%)**	**$\bar{\tau}$ (ns)**
TiO_2	1.17	42.66	9.29	57.34	5.83
Mg:TiO_2	0.94	43.57	13.00	56.43	7.75
Ca:TiO_2	0.87	35.21	10.48	64.79	7.10
Sr:TiO_2	1.26	43.62	13.18	56.38	7.80
Ba:TiO_2	1.59	37.97	21.02	62.03	13.64

Supplementary Table 4. Electrochemical impedance spectroscopy (EIS) fitting results of various samples

	**TiO_2**	**Mg:TiO_2**	**Ca:TiO_2**	**Sr:TiO_2**	**Ba:TiO_2**
R_s (Ω cm^{-2})	72.39	90.19	84.63	80.73	98.03
C_{dl} (F cm^{-2})	1.73 x 10^{-7}	1.62 x 10^{-3}	1.89 x 10^{-5}	1.77 x 10^{-5}	2.05 x 10^{-5}
R_{ct} (Ω cm^{-2})	6.99 x 10^{4}	5.41 x 10^{4}	2.52 x 10^{4}	2719	1123
χ-squared	1.36 x 10^{-2}	1.25 x 10^{-2}	2.75 x 10^{-3}	2.25 x 10^{-2}	3.01 x 10^{-2}

Reference

1 Momma, K. & Izumi, F. VESTA 3 for three-dimensional visualization of crystal, volumetric and morphology data. *J. Appl. Crystallogr.* **44**, 1272–1276, (2011).

2 Feng, C. *et al.* Synthesis of leaf-vein-like *g*-C_3N_4 with tunable band structures and charge transfer properties for selective photocatalytic H_2O_2 evolution. *Adv. Funct. Mater.* **30**, 2001922, (2020).
