# Supplementary material for: Lactic acid photosynthesis via C–C cross-coupling over atomically dispersed Ba
Source: Nat Commun. 2026 May 29;17:6984. doi: 10.1038/s41467-026-73727-4 (PMC13392379; doi:10.1038/s41467-026-73727-4)
Supplement: Supplementary file 2 — Description of Additional Supplementary Files [file 41467_2026_73727_MOESM2_ESM.pdf]

Description of Additional Supplementary Files

Supplementary data 1

Optimized computational models for calculating projected density of states upon anatase (1 0 1).

Supplementary data 2

Optimized computational models for calculating projected density of states upon Mg:TiO_2 (1 0 1).

Supplementary data 3

Optimized computational models for calculating projected density of states upon Ca:TiO_2 (1 0 1).

Supplementary data 4

Optimized computational models for calculating projected density of states upon Sr:TiO_2 (1 0 1).

Supplementary data 5

Optimized computational models for calculating projected density of states upon Ba:TiO_2 (1 0 1).

Supplementary data 6

Optimized computational models for calculating projected density of states calculation of C and O 2*p* states of *$CHOHCH_2OH$ intermediates upon anatase (1 0 1).

Supplementary data 7

Optimized computational models for calculating projected density of states calculation of C and O 2*p* states of *$CHOHCH_2OH$ intermediates upon Ba:TiO_2 (1 0 1).
