# Supplementary material for: Lactic acid photosynthesis via C–C cross-coupling over atomically dispersed Ba
Source: Nat Commun. 2026 May 29;17:6984. doi: 10.1038/s41467-026-73727-4 (PMC13392379; doi:10.1038/s41467-026-73727-4)
Supplement: Supplementary file 4 — Transparent Peer Review File [file 41467_2026_73727_MOESM4_ESM.pdf]

nature portfolio

Peer Review File

Lactic acid photosynthesis via C–C cross-coupling over atomically dispersed Ba

Corresponding Author: Professor Peng Zhou

This file contains all reviewer reports in order by version, followed by all author rebuttals in order by version.

Version 0:

Reviewer comments:

Reviewer #1

(Remarks to the Author)
In this work, the authors report a TiO_2 photocatalyst derived from a metal-organic framework (MOF) featuring atomically dispersed barium (Ba) species for the cross-coupling of ethylene glycol and methanol to produce lactic acid. It is particularly noteworthy that the authors utilized terephthalic acid (a degradation product of PET) to construct the MOF, while subjecting another PET-derived molecule, ethylene glycol, to a carbon-enrichment reaction yielding valuable C3 compounds. This strategy elegantly demonstrates a novel and scalable pathway for the upcycling of waste plastics. For the catalytic reaction, the authors skillfully leveraged that high atom dispersibility of MOF to fabricate TiO_2 materials with atomically dispersed Ba species, thereby enhancing the inherent Lewis acid-base properties of the TiO_2 surface. This modification promotes the formation of critical transient and steady-state intermediates, achieving an optimal balance between reaction activity and selectivity. As a result, the target product was obtained with high selectivity and activity without the use of any precious metals. Moreover, the anaerobic reaction environment enables the co-production of valuable H_2, rendering the overall process highly atom-economical. Overall, this study makes a significant contribution to the fields of plastic upcycling and photocatalytic C–C coupling, demonstrating rigorous experimental design and compelling results. I commend the authors for their excellent work and support its publication in Nature Communications pending appropriate revisions.

1. The concept of balancing “transient” and “stable” intermediates is the core and innovative aspect of the author’s mechanism description. The author should expand the discussion to explicitly show how this balance suppresses undesired side reactions compared to conventional “radical–radical” or “adsorbed–adsorbed” coupling routes.

2. The manuscript mentions “no other reduction products”, but quantitative data on liquid-phase byproducts are lacking and should be provided.

3. Photoluminescence quenching and prolonged carrier lifetimes in Ba:TiO_2 convincingly indicate improved charge separation. It would further strengthen the mechanistic understanding if the authors could elaborate on how “gap states near the Fermi level” act as “relay stations” or “electron traps” that facilitate this effect.

4. Some sections contain grammatical or structural inconsistencies (e.g., the first sentence of the Introduction is incomplete, and parts of the Discussion are repetitive). There are also minor typographical errors (“electivity” should be “selectivity”). A careful language review by a native English editor is recommended.

5. Essential parameters such as excitation and detection wavelengths in PL spectroscopy and light intensity in photocatalytic tests should be clearly specified to enhance reproducibility.

6. A systematic comparison with existing photocatalytic/electrocatalytic C–C coupling systems (activity, selectivity, stability, and cost, etc.) is needed to better contextualize the advantages and innovation of this work. Furthermore, authors should provide standardized descriptors such as AQY or solar-to-chemical efficiency for peer comparison.

Reviewer #2

(Remarks to the Author)

Zhou et al. and co-authors report photocatalytic C-C coupling to form C3+ products from plastic-derived ethylene glycol (EG) using atomically dispersed Ba species on TiO2. The topic for selective upcycling of plastics to higher-value chemicals is timely. The chemistry is compelling and the dataset is substantial, but in its present form the manuscript is not suitable for Nature Communications. The writing lacks clarity and logical flow, with obvious typographical errors (e.g., line 39 in the abstract; line 67 in the introduction), and the introduction does not articulate a clear rationale or hypothesis for using Ba/TiO2 to achieve unique C–C coupling from EG. Also, critical controls are missing: MOF-derived TiO2 from MIL-125(Ti) calcined in O2 at 500°C for 3 h can retain residual carbon/carbonates that may bias product distributions. Please provide relative data to exclude this confound, and establish product provenance with a carbon balance and 13C-EG labelling. Evidence for atomically dispersed Ba needs strengthening, and the role of alkaline-earth Ba should be discussed beyond HER thermodynamics, specifically why it does not narrow the TiO2 band gap and how it modifies vacancies, Fermi level, and Lewis basicity to enable C–C coupling. Performance reporting should include replicates mass balances, AQY, and Ba-leaching tests, and clear selectivity definitions. With substantial revision addressing these points, the study could be reconsidered.

Reviewer #3

(Remarks to the Author)
In their manuscript, Wang et al. report a TiO2-based photocatalysts for the joint production of lactic acid and hydrogen. While the activity of their most active Ba-TiO2 material is very encouraging, some of the more in-depth mechanistic analysis requires substantial clarification. The following comments should be addressed before the manuscript can be published. In no particular order:

- several SI figures are of poor image quality and barely readable (e.g. S6, S20). Please check all figures for readability and use higher resolution as needed.
- how exactly does Ba insert (interstitial, …)?
- the PL data (Fig S19) has some datapoints indicated, but then more datapoints in between as suggested by the noise. Please clarify the true wavelength resolution
- "the negative signal in Ba:TiO2 undergoes a red shift over time, and its excited-state lifetime is significantly shorter than that of Ba:TiO2" does not seem to make sense. Please clarify.
- The TAS signals in Fig 4 are very small. What is the resolution limit of the used setup? How do the authors mitigate the risk of overinterpretation of transient features that are barely above the resolution limit?
- Also on TAS: what is the feature seen around 490 nm? If excitation was done at 350 nm, it cannot be excitation scatter.
- The statement "In general, species A represents the initial excited state or thermal carriers, while species B corresponds to the charge-separated state after relaxation" seems hard to justify, given that the component spectra (Fig 4F) are very different between TiO2 and Ba-TiO2. As such, it seems unlikely that both components report on the same species in both materials.
- How did the authors determine that 2 components should be used for the TA global analysis? Does the fit quality improvement over a 1-parameter-fit justify the addition of an additional parameter, especially given the noise level of the data? Fit criteria such as AICc (Corrected Akaike Information Criterion) or BIC (Bayesian Information Criterion) would be useful to support this choice.
- incomplete sentence: "Structurally, the conversion of methanol and ethylene glycol to lactic acid inherently involves the."
- what are the apparent quantum yields (AQYs) for the LA and H2 production reactions? Please report for at least one wavelength.
- Did the authors consider non-surface-Ba acting as hole trap, given that Ba is distributed throughout the entire material? The effects of bulk Ba on the optical measurements conducted should be mentioned.
- the claimed single atom nature of the Ba sites is not demonstrated convincingly - the relatively blurry images in Fig. 2 and insufficient to fully support this claim. I recommend the authors conduct experiments that could demonstrate the single atom nature (e.g. EXAFS/XANES) or leave open the possibility that the Ba sites may be small Ba clusters rather than single atoms.
- The PL spectra presented in Fig. 4 in the main text are much redshifted compared to those in Fig. S19. Why?
- the PL spectra shown in Fig. S4 lie relatively far from the bandgap energy. The authors state that they investigate the exciton binding energy via the temperature dependence, however, this is only the case if emission occurs from the band state. In this case, the PL occurs relatively far from the bandgap energy, and is thus more likely investigating PL related to trap states; as such, it is unlikely that the Arrhenius activation energies are reflective of the excition binding energy. This suggests that the statement "indicate that the alkaline earth metal introduction can minimize the Eb" is unsubstantiated.
- "Through in-situ XPS spectra analysis of Ba:TiO2, it was determined that under illumination, the photo-generated electrons migrate toward Ti and Ba metal sites while holes migrate toward lattice oxygen sites": it seems the main differences are between TiO2 and Ba-TiO2, not Ba-TiO2 in the dark vs under illumination. Therefore, it is unclear where this conclusion comes from. Please clarify.
- the authors address the "depletion of C–OH bond" in Fig 5. What is the signature of this depletion? It seems there are only signals that increase over time, not decrease - at most a constant amplitude around 1110 cm-1.
- while the poisoning experiments with pyrrole and pyridine imply cooperative acid–base involvement, an FLP requires acid and base sites to be adjacent in order to operate as a paired site under reaction conditions. So far, the authors do not demonstrate this spatial proximity - please clarify.

Version 1:

Reviewer comments:

Reviewer #1

(Remarks to the Author)
The comments have been addressed. I think it can be accepted for publication.

Reviewer #2

(Remarks to the Author)
The authors have corrected the work to publication level, so I recommended this to be published on Nature Communication.

Reviewer #4

(Remarks to the Author)
The manuscript reports a series of alkaline earth metal doped TiO2 (M-TiO2) photocatalysts for photocatalytic upcycling of ethylene glycol to C3 compounds. The manuscript presents a combination of experimental and computational studies to rationalize the claim that the implantation of alkaline earth metal cations promotes both selectivity and activity. However, the current results are superficial. Especially, some key results are self-contradicted (see my comments below). Therefore, I cannot recommend the publication of this work in Nature Communications.
1. Major issues in catalytic performance
I) As reported by the authors, Ba-TiO2 shows a production rate of LA of 2.8 mmol g–1 h–1 (corresponding to 14 mol h–1, 5 mg catalysts used in the reaction) under a 360-mW cm–2 light irradiation, and meanwhile, demonstrates a AQY of 3.75% at 350 nm (light intensity of 22.9 mW cm–2). When taking the AQY value into the Eq. S1, the production rate of LA under 350-nm light irradiation can be calculated to be 22 mol h–1, even much higher than that obtained under normal conditions. This is unreasonable. Furthermore, even at 420 nm where Ba-TiO2 basically has no light absorption, the AQY still shows to be around 0.5%. The AQY values seem to be heavily overestimated in the study.
II) The authors plotted the standard curves of different products at concentrations ranging from 10 mM to 100 mM. However, according to the activity data listed in Table S2, the actual concentration of the products in the reaction solution was in the range of 0 - 2.1 mM, which deviated significantly from the concentration range of the standard curves. This situation would lead to significant error in quantitative analysis.
III) The HPLC spectra for all M-TiO2 are lacking, which leads to the difficulty for reviewers to access the reliability of activity data.
IV) In Figure S32, the authors provide the HPLC spectra of TiO2 recorded before and after reaction. A significant quantity of glycolic acid was detected before the reaction. Why? The signals of lactic acid and formic acid are too weak to be analyzed quantically. From what sources do the signals at 5.5, 6, 6.5, 9.8, and 10.2 min originate? Moreover, why did these signals change after the reaction?
V) The authors subjectively attribute the activity loss in cycling measurements to catalyst loss. Therefore, to minimize catalyst loss, they developed a photocatalytic system composed of a continuous-flow reactor. However, they conducted only a single-time activity test using this system, and the stability of this system was not investigated. Moreover, the original HPLC spectrum data is not given.
2. Major issues in theoretical calculations
I) The study performed the theoretical calculations on the free energy change in the formation pathway of lactic acid over TiO2 and M-TiO2. The results seem to explain the higher activity of M-TiO2 than TiO2. However, the calculations on the pathways of other products are lacking. For example, CH3OH to HCHO, CH3OH to HCOOH, CH3OH to ethylene glycol and ethylene glycol to glycolic acid. These reactions compete with the formation of lactic acid. comparing these pathways, the conclusion of this section, which the authors claimed as "the activity and selectivity for LA production on M - TiO2 are greatly enhanced, particularly with the introduction of Ba", cannot be drawn.
II) According to the DOS calculations in Figure 1d, it can be seen that barium implantation create interstitial states near the valence band of TiO2. This finding raises two questions. 1) Why does Ba-TiO2 show an increased bandgap? 2) The interstitial states from Ba prefer to act as the hole accepter rather than electron accepter because they are closer to the valence band of TiO2. However, the experimental results give an oppositive conclusion.
III) Actually, the position of Fermi level in DOS is set in calculations but not the actual position. This conceptual and fundamental error in this study is unacceptable and misleading ("the barium species create interstitial states near the Fermi level" in the abstract).
3. Other issues
I) In the activity measurements, a strong alkaline medium (4.0 M) was used. Previous studies demonstrate that the alkaline conditions could lead to the oxide surface more negatively charged and promote the formation of hydroxyl radical (J. Am. Chem. Soc. 2012, 134, 1974. Nat. Mater. 2014, 13, 1013.). However, this study gives the totally oppositive conclusion (Figures S33 and S34).
II) The classic study by Koper et al demonstrates that the first deprotonation of alcohols in alkaline solution is base catalyzed (J. Am. Chem. Soc. 2011, 133, 6914.). The authors ignore this situation in all discussion.
III) In Figure 5c, the authors attribute the ESR signal to hydroxymethyne radical (αN = 15.1, and αH = 21.4) from the EG transformation. However, ·CH2OH radical (αN = 15.6, and αH = 21.5) show the similar signal at the very close position. Therefore, the current assignment is doubtable.
IV) When carefully comparing the in-situ FTIR spectra of Ba-TiO2, TiO2 and the neat ATR crystal, it would be found that all labeled absorption peaks for Ba-TiO2 can be also found in the spectra of TiO2 and neat ATR crystal, while they present the different assignments. This is self-contradicted. Moreover, why do the spectra of the neat ATR crystal also show the signals from COO, CO, CH3?
V) In the scheme, the coupling between two *CH3 to form CH3CH3 seems more energetically favorable than the coupling

between *CH3 and hydroxymethyne radical, considering the steric effect.
VI) The signal intensity of TA is too weak. High quality data should be provided.
VII) In the in-situ XPS spectra, the shift of Ba3d3/2 is not consistent with that of Ba3d5/2; the shift of Ti2p3/2 is not consistent with that of Ti2p1/2. Theoretically, the shift should be same.
VIII) The implantation of Ba leads to the lattice expansion, meaning that length of the Ti-O bond is increased. Thus, the strength of the Ti–O bond should be weakened. However, the Raman spectra confirm the enhanced Ti–O bond. An explanation should be provided.
IX) The Raman signal above 1000 cm-1 should be presented to confirm the presence of residual carbon in the catalysts or not.
X) Captions of Figure 1b and 1c are muddled up.
XI) A careful language review is recommended. For example, "owing to the introduction of alkaline earth metals, the strength of Ti–O bonds is enhanced, leading to the absorption edges of M:TiO2 (ca. 430 nm), which results in a slight blue shift compared with TiO2 (ca. 450 nm)."

Reviewer #5

(Remarks to the Author)
I have accessed the revised manuscript and can conclude that a lot of work has been put in addressing the reviewers' comments. Specifically, to the comments of Referee 3, most of the technical comments have been clarified (data quality, additional measurements, QY calculations, clarification of the PL datasets and optimisation of the statements made in the main manuscript). Nevertheless, several comments have been addressed less satisfactorily. As such:

1) Ref 3, Q12: I would still agree with the referee's critisims: experimental data presented in the MS (also in its revised version) confirm Ba incorporation by detecting Ba-O bonds, but it is not an indicator for Ba single atoms. Ba-based oxo-clusters on TiO2 would show a similar bonding situation and thus cannot be excluded. The authors conduct EXAFS and suggest "Especially, the fitting results of EXAFS further confirms the atomically dispersed nature of Ba coordinated with six O atoms, consistent with the above theoretical model", however it is unclear which model is meant. The one shown in Figure 1a features only surface Ba atoms which are clearly not 6-fold coordinated.

2) Ref 3, Q11: on a similar note, not enough information is given in the DFT section on the actual atomistic models used in the calculations: what are the explored positions of Ba, how have its optimized locations been determined? DFT models presented at the beginning of the MS seem to consider surface Ba (surface decoration and its effect on catalysis), but experiments shown later also reveal that Ba incorporates inside the material as a dopant. This seems not to be considered in the DFT models. This creates dissonance: when modelling surface reactions, Ba inside the lattice is ignored; when modelling the structure, Ba on the surface is ignored. This requires more clarification and the reveal of the details of the DFT models and their progressive optimisation.

Besides this, a few other comments from my side are

3) Scheme 1: right bottom: „sites", „cleaving"
4) Another comment on the choice of the DFT model: how strongly does the chosen anatase surface correspond to or represent the actual MIL-125-derived TiO2 NPs? The surface shown in the simulation is flat. The derived NPs (Figure 2e) are not faceted. Ba location is thus not justified.
5) What are "dual" Ba-O bonds? Double?
6) If the authors suggest that Ba replaces Ti in the lattice, this needs to be stated clearly throughout the MS text (refer to Ref 3, Q2).
7) I don't see the indicated absorption edges from Figure S22. For example, TiO2 does not absorb "from 450 nm". This would also go completely against the known anatase band gap values reported in the literature.

Version 2:

Reviewer comments:

Reviewer #4

(Remarks to the Author)
The reviewer appreciates the authors' response to the comments. They did some supplementary experiments to address my concerns, but some issues still remain.
1. The authors attributed the inconsistence in activity data between normal conditions and AQY test conditions to the employment of different light source. To support their claim, they should provide the light intensity profiles of the broadband light source and the monochromatic light source.
2. The authors attributed the non-zero AQY observed at 420 nm to the excitation of the residual high-energy light. However, the similar situation also happened for the AQY tests at other wavelengths. This means all reported AQY values are overestimated.
3. The standard curves of different products were updated, but the activity data were not corrected.
4. From the standard HPLC spectra of different products shown in Fig. S17, the peak positions for formic acid and glycolic acid are very close, which would interfere their quantitative analysis.

5. When checking the relative peak intensities for lactic acid, formic acid, and glycolic acid in the HPLC spectra of Ba:TiO2 in Fig. S25, the selectivity of lactic acid seem to be not as high as the reported.
6. In the DOS plots of Ba:TiO2, the interstitial states from Ba are close to the top of valence band, it means they prefer to accept holes from the valence band or the occurrence of the excitation from the interstitial states to the conduction band. This is against to the authors' core claim.
7. The claim that the barium species create interstitial states near the Fermi level is not correct, because the Fermi level is set to energy zero. The relative positions of CB, VB and interstitial states to the Fermi level are invalid in the present DFT calculations. For example, the current calculations show the Fermi level is close to VB for all materials, but the actual situation is that the Fermi level is close to CB due to their n-type feature.
8. The authors attributed the discrepancy between XRD and Raman to the fact that XRD and Raman spectroscopy probe different crystallographic directions and vibrational modes of the Ti–O framework. However, in fact, all XRD peaks of Ba:TiO2 are shifted to larger angles relative the corresponding peaks of TiO2.

Reviewer #5

(Remarks to the Author)
Both my comments 1 and 2 (based on the original Ref 3 comments) have not been adequately addressed. The presence of Ba-oxo clusters cannot be ruled out. The details on the fundamental structural models have not been provided.

In response to my comment 4, the authors accept my criticism that the model of Ba surface atoms on Ti(001) is not consistent with the realistic structure of TiO2 obtained after MIL-125 calcination. This fact calls into question all mechanistic results from DFT.

Version 3:

Reviewer comments:

Reviewer #4

(Remarks to the Author)
My concerns have been addressed.

Reviewer #5

(Remarks to the Author)
To R1(a): the authors show no evidence of the absence of Ba-Ba distances in the second coordination shell - neither in their response, nor in their revised MS or ESI. The sentence "However, such a feature is absent in the experimental EXAFS spectra" is thus not supported by the data or the presented analyses.

Figure R7: Given the high atomic number of Ba vs Ti, I would expect the single atoms of Ba to be clearly visible in the TEM image. However, even if the authors highlight the "single atoms" with circles, the contrast differences are marginal, rendering these images unreliable as proof.

The remaining comments or issues have been satisfactorily addressed.

Version 4:

Reviewer comments:

Reviewer #5

(Remarks to the Author)
My concerns have been addressed. My congratulations to the authors on this outstanding work.

Open Access This Peer Review File is licensed under a Creative Commons Attribution 4.0 International License, which permits use, sharing, adaptation, distribution and reproduction in any medium or format, as long as you give appropriate credit to the original author(s) and the source, provide a link to the Creative Commons license, and indicate if changes were made.
In cases where reviewers are anonymous, credit should be given to 'Anonymous Referee' and the source.
The images or other third party material in this Peer Review File are included in the article's Creative Commons license, unless indicated otherwise in a credit line to the material. If material is not included in the article's Creative Commons license and your intended use is not permitted by statutory regulation or exceeds the permitted use, you will need to obtain permission directly from the copyright holder.
To view a copy of this license, visit https://creativecommons.org/licenses/by/4.0/

Response to the Comments of Reviewers

Nature Communications

Ms. Ref. No.: NCOMMS-25-80104

Title: *Atomically dispersed barium species enable photocatalytic upcycling of plastic-derived glycol to C_3 compounds*

Author(s): *Wei Wang, Yonghua Tang, ZhuiZhui Su, Huanmin Liu, You-Nian Liu, Dingguo Tang, and Peng Zhou**

Reviewer #1

In this work, the authors report a TiO_2 photocatalyst derived from a metal-organic framework (MOF) featuring atomically dispersed barium (Ba) species for the cross-coupling of ethylene glycol and methanol to produce lactic acid. Overall, this study makes a significant contribution to the fields of plastic upcycling and photocatalytic C–C coupling, demonstrating rigorous experimental design and compelling results. I commend the authors for their excellent work and support its publication in Nature Communications pending appropriate revisions.

Q1: *The concept of balancing "transient" and "stable" intermediates is the core and innovative aspect of the author's mechanism description. The author should expand the discussion to explicitly show how this balance suppresses undesired side reactions compared to conventional "radical–radical" or "adsorbed–adsorbed" coupling routes.*

R1: We thank the reviewer for this constructive comment. In our system, the transient intermediates (alkyl radicals, •R) provide the high reactivity required for C–C coupling, whereas the stable intermediates (adsorbed alkyl species, *R) guide the reaction pathway by stabilizing only the productive transformation steps. This complementary interplay enables the reaction to avoid the two conventional pathways that are prone to side reactions. Such as, radical–radical coupling requires two freely diffusing •R to appear simultaneously, which often leads to over-coupling and the formation of oligomers or combustion-type byproducts. Conversely, when two *R species are fully adsorbed on adjacent sites, their strong binding can slow turnover and increase the probability of unwanted C–C recombination or deep oxidation. Our mechanism circumvents this issue by keeping only one reactant stably adsorbed, while the other remains transient. This arrangement ensures directional coupling and minimizes competitive adsorption or non-selective surface reaction. Maintaining this dynamic balance allows the reaction to proceed through a controlled single-site pathway, enhancing selectivity and suppressing the formation of undesired products.

Following the reviewer's suggestion, we have revised the *Introduction* (**Lines 46–58**) to provide a clearer and more concise explanation of how the transient and stable intermediates cooperatively regulate reaction kinetics and limit side reactions. All revised text is highlighted in yellow in the updated manuscript.

*Recent efforts have attempted to convert ethylene glycol and methanol into valorized C_3 chemicals, such as lactic acid. However, electrochemical routes, suffer from an intrinsic trade-off, i.e., low applied biases fail to activate inert C–H and C–O bonds, whereas high biases drive excessive oxidation and lead to considerable losses in selectivity. Heterogeneous photocatalysis offers a promising, milder alternative that enables bond activation and molecular coupling under ambient conditions. Nevertheless, the success of such photocatalytic C–C coupling reactions strongly depends on the selective generation and regulation of key intermediates. Productive C–C coupling typically proceeds through either adsorbed alkyl species (*R) or transient radical species (•R). Coupling between two surface-bound intermediates is kinetically slow due to their limited reactivity (Scheme 1a), while coupling between two radicals is difficult to control because of their short lifetimes and the tendency for uncontrolled radical-radical recombination (Scheme 1b). These competing pathways often result in low coupling efficiency and poor product selectivity.*

Therefore, the central scientific challenge lies in establishing a catalytic system that can simultaneously generate (i) a table surface-anchored intermediate with moderated reactivity and (ii) a transient radical species with sufficient activation energy, while ensuring their rapid and selective cross-coupling (Scheme 1c).

Q2: *The manuscript mentions "no other reduction products", but quantitative data on liquid-phase byproducts are lacking and should be provided.*

R2: Thank you for your professional comments. In the liquid-phase products of the reaction, glycolic acid and formic acid, which are oxidation products, were observed (Fig. S32), while no other oxidation was found. Instead, only H_2 was detected as the sole reduction product in the gas-phase products. Following the reviewer's suggestion, we have provided a table in *Supporting Information* to present the necessary quantitative results for the products (Table S2).

Table S2. Load amounts of alkaline earth metals in TiO_2

	TiO_2	$Mg{:}TiO_2$	$Ca{:}TiO_2$	$Sr{:}TiO_2$	$Ba{:}TiO_2$
D,L-lactic acid (mmol g^{-1} h^{-1})	*n.a.*	*1.5*	*1.8*	*2.1*	*2.8*
Formic acid (mmol g^{-1} h^{-1})	*n.a.*	*0.3*	*0.3*	*0.4*	*0.4*
Glycolic acid (mmol g^{-1} h^{-1})	*n.a.*	*0.1*	*0.1*	*0.1*	*0.2*
Oxalic acid (mmol g^{-1} h^{-1})	*n.a.*	*n.a.*	*n.a.*	*n.a.*	*n.a.*

Q3: *Photoluminescence quenching and prolonged carrier lifetimes in Ba:TiO_2 convincingly indicate improved charge separation. It would further strengthen the mechanistic understanding if the authors could elaborate on how "gap states near the Fermi level" act as "relay stations" or "electron traps" that facilitate this effect.*

R3: We thank the reviewer for this valuable suggestion. The Ba-induced gap states located near the Fermi level act as shallow electron-trapping sites (Fig. 1d). These states can temporarily capture photoexcited electrons on a sub-nanosecond timescale, which lowers the probability of their immediate radiative recombination with holes. At the same time, these gap states function as reversible traps, where the captured electrons can be thermally released or transferred through Ti–Ti hopping. This rapid trapping–detrapping cycle increases the spatial separation between electrons and holes, thereby suppressing radiative recombination and prolonging carrier lifetimes. Following the reviewer's suggestion, we have added this clarification to the revised manuscript (**Lines 117–120**), and all updated text is highlighted in yellow.

A band structure analysis showed that only the Ba species can generate strong interstitial states slightly above valence band (Fig. 1d). Therefore, Ba doping produces little effect on the band gap of TiO_2. Additionally, Fermi level is directly localized in the region of Ba 6p states, implying that Ba site can effectively capture electrons from valence band for promoting charge separation.

Q4: *Some sections contain grammatical or structural inconsistencies (e.g., the first sentence of the Introduction is incomplete, and parts of the Discussion are repetitive). There are also minor typographical errors ("electivity" should be "selectivity"). A careful language review by a native English editor is recommended.*

R4: We thank the reviewer for highlighting these issues. We have thoroughly revised the manuscript to address the grammatical inconsistencies, structural problems, and typographical errors noted, including the correction of "electivity" to "selectivity". The text has also been polished by a professional English editor to ensure clarity and accuracy throughout.

Q5: *Essential parameters such as excitation and detection wavelengths in PL spectroscopy and light intensity in photocatalytic tests should be clearly specified to enhance reproducibility.*

R5: We thank the reviewer for this important comment. All essential experimental parameters have now been clearly specified to improve reproducibility. In particular, the excitation and detection wavelengths for PL measurements, as well as the illumination conditions for the photocatalytic test, have been added to the revised manuscript.

Line 302: ***Fig 4. Separation and transfer behaviors of photo-generated carriers. (a)*** *Transient PL spectra of TiO_2 and M:TiO_2 samples (λ_{ex}: 350 nm, $\lambda_{monitoring}$: 450 nm). Temperature-dependent PL spectra for* ***(b)*** *TiO_2 and* ***(c)*** *Ba:TiO_2, λ_{ex}: 350 nm. TAS spectra of* ***(d)*** *TiO_2 and* ***(e)*** *Ba:TiO_2 under 350-nm excitation, and* ***(f)*** *corresponding SAS spectra.* ***(g)*** *In-situ XPS spectra of Ba:TiO_2 under illumination for 15 min.*

Line 440: *Then, the reactor was placed in a constant temperature water bath at 25 °C and irradiated under an Xe lamp (360 mW cm^{-2}) for 3 hours.*

C6: ***(a)*** *A systematic comparison with existing photocatalytic/electrocatalytic C–C coupling systems (activity, selectivity, stability, and cost, etc.) is needed to better contextualize the advantages and innovation of this work.* ***(b)*** *Furthermore, authors should provide standardized descriptors such as AQY or solar-to-chemical efficiency for peer comparison.*

R6: We thank the reviewer for this suggestion. **(a)** We note that, to the best of our knowledge, there is currently no reported photocatalytic system that achieves direct C–C cross coupling of methanol and ethylene glycol. For comparison, a recent study reports the **electrocatalytic cross-coupling** of ethylene glycol with primary C_1–C_4 alcohols to produce α-hydroxycarboxylic acids over a **gold catalyst**. In that work, stabilization of aldehyde intermediates enabled subsequent aldol condensation, leading to lactic acid with a reported productivity of 268.1 μmol cm^{-2} h^{-1} at 100 mA cm^{-2}. While notable, **such electrocatalytic systems require noble-metal catalysts and complex electrochemical setups**. In contrast, our photocatalytic system directly couples alcohols under light-driven conditions, achieving high reaction rates using earth-abundant catalysts and a simple experimental setup. This demonstrates a complementary, sustainable, and practical approach to carbon-chain elongation that is distinct from existing electrocatalytic strategies. We have included a concise discussion of this comparison with existing catalytic systems in the revised manuscript to clearly highlight the advantages of our system (**Lines 202–202**).

(b) As requested, descriptions of the apparent quantum yield (*AQY*) have been added to the revised manuscript (**Lines 198–200**), and the corresponding data are provided in the *Supplementary Information* (Fig. S15) to facilitate peer comparison.

Lines 200–202: *Meanwhile, compared to previous electrocatalytic systems for C–C cross-coupling, which rely on stabilized aldehyde intermediates under applied current, our photocatalytic approach achieves direct C–C coupling under light-driven conditions, representing a complementary and sustainable strategy.*

Lines 195–197: *The photocatalytic coupling performance of Ba:TiO_2 was further tested by the action spectra, namely the apparent quantum yield (AQY). As shown in Fig. S15, the AQY (for the synthesis of LA) of Ba:TiO_2 exhibits a positive correlation with its UV–vis DRS spectrum, demonstrating an AQY of 3.75 ± 0.05% at 350 nm (5 mg of photocatalysts).*

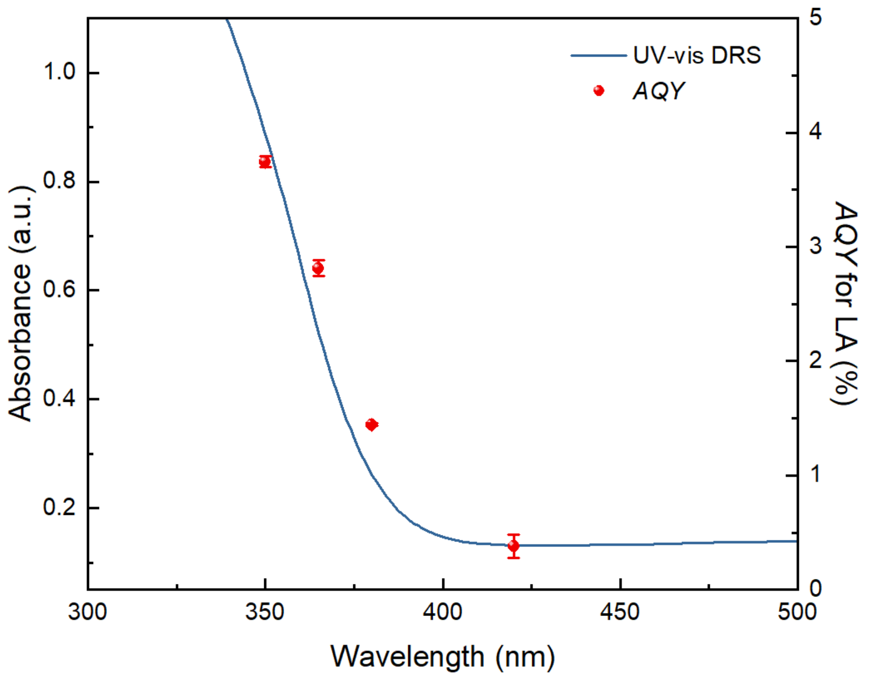

Fig. S15 *The apparent quantum yield of the Ba:TiO*$_2$*. Catalyst mass: 5 mg. Light intensity: 22.9 mW cm*$^{-2}$ *(350 nm); 26.5 mW cm*$^{-2}$ *(365 nm); 28.5 mW cm*$^{-2}$ *(380 nm); 29.6 mW cm*$^{-2}$ *(420 nm). Irradiated area: 20 cm*^{2}*. A minimum of three replicate measurements was performed for each material group to ensure reproducibility (*$n \geq 3$*).*

Reviewer #2

Zhou et al. and co-authors report photocatalytic C–C coupling to form C_{3+} products from plastic-derived ethylene glycol (EG) using atomically dispersed Ba species on TiO_2. The topic for selective upcycling of plastics to higher-value chemicals is timely. The chemistry is compelling and the dataset is substantial, but in its present form the manuscript is not suitable for ***Nature Communications***. With substantial revision addressing these points, the study could be reconsidered.

Q1: *The writing lacks clarity and logical flow, with obvious typographical errors (e.g., line 39 in the abstract; line 67 in the introduction), and the introduction does not articulate a clear rationale or hypothesis for using Ba/TiO_2 to achieve unique C–C coupling from EG.*

R1: We thank the reviewer for pointing this out. The typographical error "electivity" in **Line 35** has been corrected to "carbon selectivity". The incomplete sentence in **Line 58** has also been revised with "*From a structural standpoint, the conversion of methanol and ethylene glycol inherently requires the cleavage of both C–H and C–O bonds*". In addition, the entire manuscript has been carefully revised to eliminate similar grammatical and typesetting issues.

Following the reviewer's suggestion, we have substantially improved the clarity and logical framing of the *Introduction*. Specifically, we have added a concise rationale for employing Ba/TiO_2 to enable direct C–C coupling from ethylene glycol and clarified the underlying mechanistic hypothesis regarding Ba-induced modulation of charge separation and intermediate binding. These additions are now incorporated in the revised manuscript.

Lines 46–66: *Recent efforts have attempted to convert ethylene glycol and methanol into valorized C_3 chemicals such as lactic acid. Electrochemical routes, however, suffer from an intrinsic trade-off, namely, low applied biases fail to activate inert C–H and C–O bonds, whereas high biases drive excessive oxidation and lead to considerable losses in selectivity. Heterogeneous photocatalysis offers a promising, milder alternative that enables bond activation and molecular coupling under ambient conditions. Nevertheless, the success of such photocatalytic C–C coupling reactions strongly depends on the selective generation and regulation of key intermediates. Productive C–C coupling typically proceeds through either adsorbed alkyl species (*R) or transient radical species (•R). Coupling between two surface-bound intermediates is kinetically slow due to their limited reactivity (Scheme 1a), while coupling between two radicals is difficult to control because of their short lifetimes and the tendency for uncontrolled radical-radical recombination (Scheme 1b). These competing pathways often result in low coupling efficiency and poor product selectivity.*

Therefore, the central scientific challenge lies in establishing a catalytic system that can simultaneously generate (i) a table surface-anchored intermediate with moderated reactivity and (ii) a transient radical

species with sufficient activation energy, while ensuring their rapid and selective cross-coupling (Scheme 1c). From a structural standpoint, the conversion of methanol and ethylene glycol inherently requires the cleavage of both C–H and C–O bonds. A catalyst featuring two distinct and cooperatively functioning active sites would, in principle, enable differentiated activation of these bonds. Frustrated Lewis pairs (FLPs), consisting of spatially separated Lewis acidic and basic sites, provide an enticing conceptual framework for enabling such dual-site chemistry. However, achieving the concurrent activation of C–H bonds in ethylene glycol and selective C–O bond cleavage in methanol via FLP-like motifs within an oxide photocatalyst remains a major unmet goal. Previous studies suggest that alkaline-earth metals can enrich the Lewis basicity of metal-oxide surfaces, stabilize photo-generated charge carriers, and enhance alkyl radical generation, indicating their potential to deliver the desired dual-site environment.

Q2: *Also, critical controls are missing: MOF-derived TiO_2 from MIL-125(Ti) calcined in O_2 at 500°C for 3 h can retain residual carbon/carbonates that may bias product distributions. Please provide relative data to exclude this confound, and establish product provenance with a carbon balance and ^{13}C-EG labelling.*

R2: We thank the reviewer for this constructive comment. The possibility that residual carbon or carbonate species from the MIL-125(Ti) precursor might bias the product distribution has been carefully evaluated. First, the precursor was calcined in flowing O_2 at 500 °C (in *Methods*), a standard condition widely demonstrated to fully remove organic ligands in MIL-125(Ti) and yield phase-pure TiO_2 (***Sci. Adv.***, 2025, 11, eadw2028; ***Nat. Mater.***, 2023, 22, 619–626). Thermogravimetric analysis confirms complete decomposition of organic components by 500 °C (Fig. S5). PXRD and Raman spectra show only signal of anatase TiO_2, without detectable carbonate bands at ~600 or ~800 cm^{-1} (Figs. 2b and S12; ***J. Phys. Chem. C***, 2024, 128, 6138–6148). Control experiments further rule out product contributions from residual carbon. Firstly, dark reactions generate only trace LA (Fig. 3b), confirming that LA arises from light-driven catalysis. The NMR signature of LA remains unchanged after 7-day storage (Fig. 3d), excluding slow desorption of pre-existing carbonaceous species. Moreover, under reaction conditions without the addition of reactants, virtually no target product (D,L-lactic acid) was detected, further confirming that the target product originated solely from the photocatalytic cross-coupling of methanol and ethylene glycol (Fig. S20). We have already emphasized this point in the revised manuscript.

Lines 204–205: *It should be further clarified that, in the dark or in the absence of reactants, only trace amounts of the corresponding oxidation (LA) and reduction (H_2) products can be detected in the Ba:TiO_2 catalyzed system (Figs. 3b and S20).*

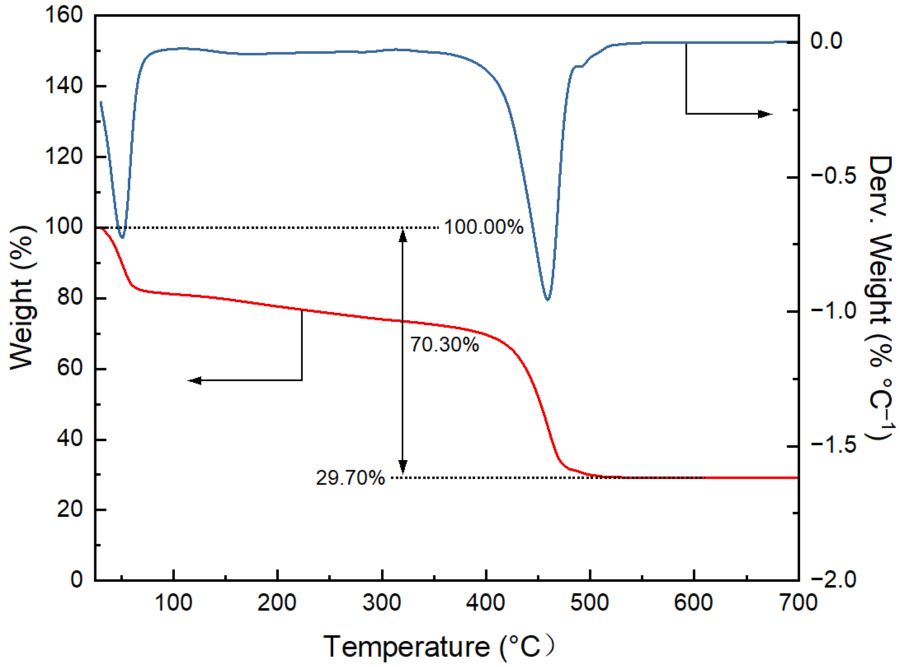

***Fig. S5** Thermogravimetric analysis result of Ba^{2+}-MIL-125(Ti) in oxygen atmosphere.*

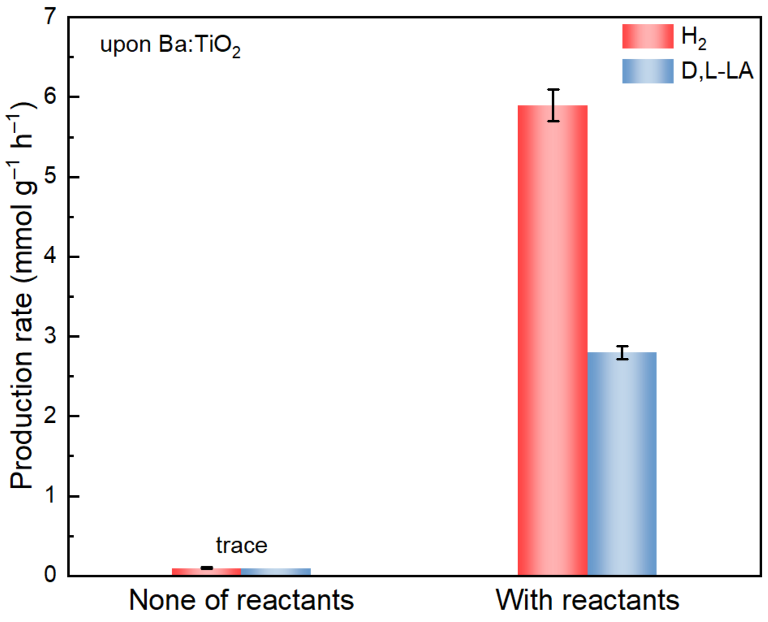

***Fig. S20** LA and H_2 production rate on Ba:TiO_2 without or with ethylene glycol and methanol.*

Q3: ***(a)** Evidence for atomically dispersed Ba needs strengthening, **(b)** and the role of alkaline-earth Ba should be discussed beyond HER thermodynamics, specifically why it does not narrow the TiO_2 band gap and how it modifies vacancies, Fermi level, and Lewis basicity to enable C–C coupling.*

R3: We appreciate the reviewer's insightful comments. In the revised manuscript, we have substantially strengthened both the experimental evidence for atomically dispersed Ba (XANES and EXAFS measurements) and the mechanistic discussion of how Ba incorporation modifies the electronic structure and surface chemistry of TiO_2 to promote C–C coupling.

(a) The atomically dispersed nature of Ba is now firmly supported by HADDF-STEM imaging, which reveals isolated bright contrast without any observable Ba clusters, together with depth-profiling XPS and FIB-SEM elemental mapping showing homogeneous low-loading Ba distribution throughout the particles. XANES and EXAFS further show that Ba:TiO_2 only has Ba–O coordination without observable detectable Ba–Ba scattering (Fig. 2h). Especially, the fitting result of EXAFS confirms the atomically dispersed nature of Ba coordinated with six O atoms, consistent with the theoretical model.

(b) In response to the reviewer's request for deeper mechanistic analysis, we now clarify that Ba incorporation modifies TiO_2 primarily through shallow electronic perturbation rather than bandgap narrowing. Firstly, DOS and UV–vis absorbance results show that Fermi level and most Ba 6*p* states are distributed slightly above the valence-band top (Fig. 1d), which produce little effect on the band-gap value of TiO_2 (Figs. S22–S25). Especially, Fermi level is directly localized in the region of Ba 6*p* states, implying that Ba site can effectively capture electrons from valence band for promoting charge separation. Moreover, both ESR and XPS confirm the absence of oxygen-vacancy-related signals (Figs. S6 and S13). Because MIL-125(Ti) is calcined in flowing O_2 at 500 °C, which is a condition known to heal rather than generate oxygen vacancies (***J. Am. Chem. Soc.***, 2025, 147, 34169–34180). Furthermore, surface Ba incorporation introduces strong surface acid-base polarization, which is critical for C–C coupling. Ba^{2+} functions as a robust Lewis acid, whereas adjacent O^{2-} species exhibit strengthened Lewis basicity, as supported by CO_2-TPD (Fig. 5e), *zeta* potential (Fig. S34), and pyridine-IR measurements (Fig. 5f). This cooperative acid-base environment resembles a surface frustrated Lewis pair and enables simultaneous activation of C–H and C–OH groups, thereby stabilizing reactive intermediates and steering their selective coupling. Consistently, selective poisoning of either acidic or basic sites completely suppresses catalytic activity, underscoring their cooperative role in enabling the unique C–C bond formation pathway.

These expanded clarifications have been added to the revised manuscript and highlighted accordingly.

Lines 154–160: *The aberration-corrected scanning transmission electron microscopy (AC-STEM) reveals that disc-like Ba:*TiO_2 *is nanoporous, and bright dots on the* TiO_2 *matrix are assigned to Ba single atoms (Fig. 2f). These nanopores with around 1–3 nm originated from the non-collapsed pore structure of MIL-125(Ti) (Fig. S9). The coordination environment of Ba species in Ba:*TiO_2 *were further investigated by Ba K-edge XANES and EXAFS analysis (Fig. 2g). The* k^3*-weighted Fourier-transformed EXAFS spectra show that Ba:*TiO_2 *only has a dominant peak at ~ 2.2 Å, assigned to Ba–O bond (Fig. S10 and S11). Especially, the fitting result of EXAFS further confirms the atomically dispersed nature of Ba coordinated with six O atoms, consistent with the above theoretical model.*

Lines 140–141: *Moreover, because it was annealed at high temperature in an oxygen atmosphere (Fig. S5), the M:*TiO_2 *photocatalyst exhibited no significant oxygen vacancies (Fig. S6).*

Lines 117–120: *A band structure analysis showed that only the Ba species can generate strong interstitial states slightly above valence band (Fig. 1d). Therefore, Ba doping produces little effect on the band gap of* TiO_2*. Additionally, Fermi level is directly localized in the region of Ba 6p states, implying that Ba site can effectively capture electrons from valence band for promoting charge separation.*

Lines 56–66: *Therefore, the central scientific challenge lies in establishing a catalytic system that can simultaneously generate (i) a table surface-anchored intermediate with moderated reactivity and (ii) a transient radical species with sufficient activation energy, while ensuring their rapid and selective cross-coupling (Scheme 1c). From a structural standpoint, the conversion of methanol and ethylene glycol inherently requires the cleavage of both C–H and C–O bonds. A catalyst featuring two distinct and cooperatively functioning active sites would, in principle, enable differentiated activation of these bonds. Frustrated Lewis pairs (FLPs), consisting of spatially separated Lewis acidic and basic sites, provide an enticing conceptual framework for enabling such dual-site chemistry. However, achieving the concurrent activation of C–H bonds in ethylene glycol and selective C–O bond cleavage in methanol via FLP-like motifs within an oxide photocatalyst remains a major unmet goal. Previous studies suggest that alkaline-earth metals can enrich the Lewis basicity of metal-oxide surfaces, stabilize photo-generated charge carriers, and enhance alkyl radical generation, indicating their potential to deliver the desired dual-site environment.*

Lines 376–381: *As shown in Fig. 5g, the addition of either pyrrole or pyridine completely quenched the photocatalytic activity. Given the atomically dispersed nature of Ba (Figs. 2e–2h), it can be inferred that each Ba Lewis acid site is coordinatively surrounded by multiple lattice oxygen Lewis base sites (analogous to the host* TiO_2 *crystal structure). This intrinsic spatial proximity, enforced by the host lattice, allows the Ba–O pairs with length of 2.42 Å to function cooperatively in the simultaneous activation of distinct bonds in the reactants, as evidenced by our DFT calculations (Figs. 1a and 1b).*

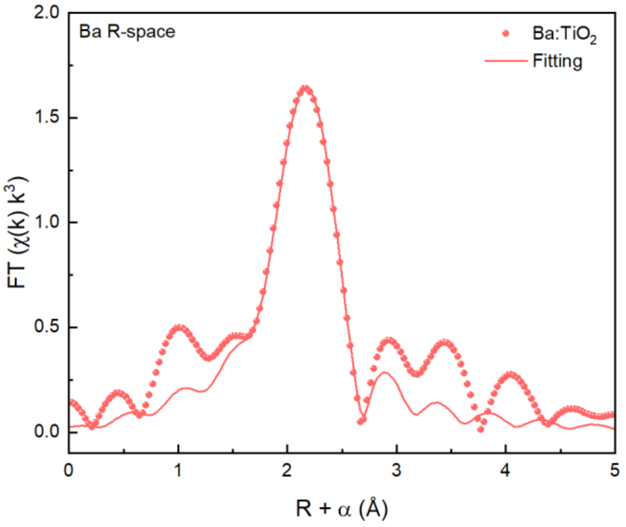

Fig. 2h *Fourier transform of* κ^3*-weighted EXAFS spectra at Ba K-edge of* $Ba:TiO_2$*.*

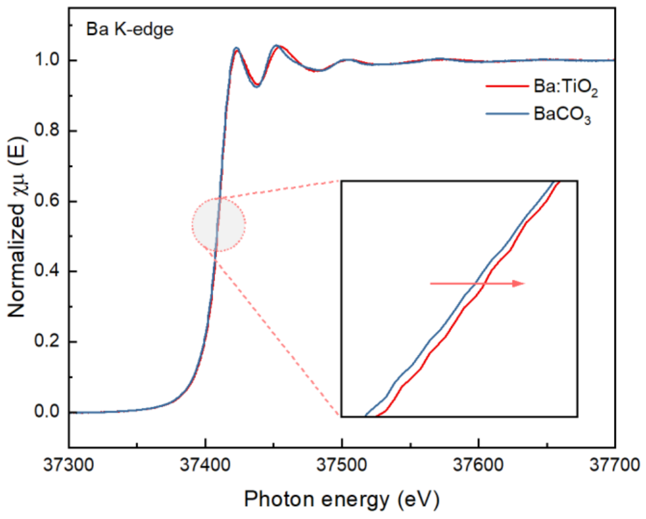

Fig. S9 *XANES spectra of Ba K-edge for Ba:*TiO_2 *and* $BaCO_3$*.*

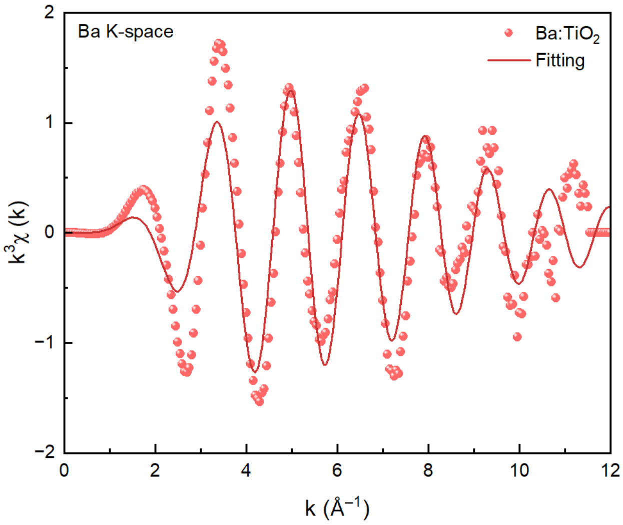

Fig. S10 *Ba K-edge EXAFS (points) and fitting curve (line) for Ba:*TiO_2*, shown in* κ^3*-weighted K-space.*

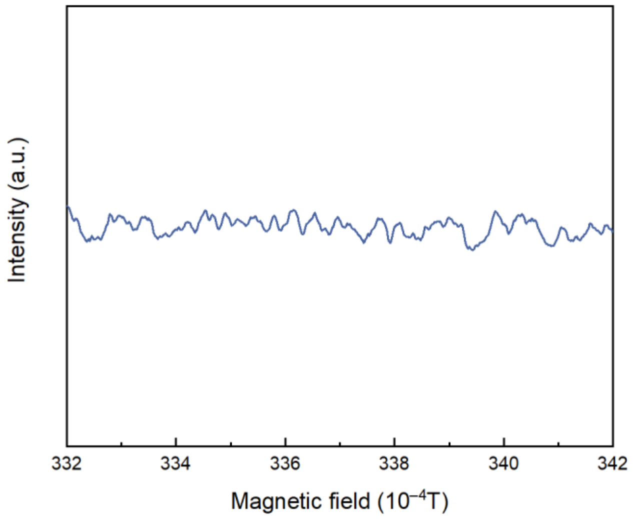

Fig. S6 *ESR spectrum of Ba:*TiO_2*.*

Q4: *Performance reporting should include* ***(a)*** *replicates mass balances,* ***(b)*** *AQY, and* ***(c)*** *Ba-leaching tests, and* ***(d)*** *clear selectivity definitions.*

R4: We thank the reviewer for these valuable suggestions regarding performance evaluation and reporting.

(a) Regarding the request for replicated mass balances, we would like to clarify that the conversion efficiency of methanol and ethylene glycol with concentration of 50.0 and 13.7 vol%, respectively, in the present system is lower than 1%. Therefore, the amount of consumed reactant (methanol and EG) cannot be accurately quantified, leading to failure in the calculation of mass balances between reactants and products. Instead, we evaluated the selectivity by calculating the ratio of carbon in all products (LA, formate and glycolic acid). The detailed equation is shown as follows:

$$C\ Sel.(\%) = \frac{3 \times N_{(\text{D,L-LA})}}{3 \times N_{(\text{D,L-LA})} + N_{(\text{formate})} + 2 \times N_{(\text{glycolic acid})}} \times 100\%$$

(1)

where $N_{(\text{D,L-LA})}$, $N_{(\text{formate})}$, and $N_{(\text{glycolic acid})}$ is the moles of produced D,L-LA, formate, and glycolic acid in the solution after the reaction.

In the original manuscript, we evaluated selectivity by calculating the ratio of photogenerated holes for producing LA and all photogenerated holes, which is also a common method to calculate the selectivity of photocatalytic reaction (***Angew. Chem. Int. Ed.***, 2025, 64, e202500747). To avoid misunderstanding, the carbon selectivity is used in the whole revised manuscript instead of hole selectivity. Theoretically, for the same product, the carbon selectivity is similar to the hole selectivity. Additionally, we provided the specific experimental conditions for the number of replicate experiments conducted for each of data measurements.

(b) We have added the apparent quantum yield (*AQY*) as a standardized and broadly comparable performance metric.

(c) For Ba-leaching tests, we compared the Ba content of Ba:TiO_2 before and after a 12-hour reaction. The initial Ba loading was 0.92 ± 0.01 wt%. After a 12-hour reaction, it remained essentially unchanged at 0.91 ± 0.01 wt%, indicating that negligible Ba^{2+} dissolution occurred during the photocatalytic process.

(d) According to above **(a)**, we used the carbon selectivity in the revised manuscript according to Equation 1.

Lines 214–215: *Moreover, the Ba content in Ba:TiO_2 remained at 0.91 wt% after a 12-hour reaction, showing minimal deviation from the initial values of 0.92 wt%, which indicates excellent catalyst stability.*

Lines 195–197: *The photocatalytic coupling performance of Ba:TiO_2 was further tested by the action spectra, namely the apparent quantum yield (AQY). As shown in Fig. S15, the AQY (for the synthesis of LA) of Ba:TiO_2 exhibits a positive correlation with its UV–vis DRS spectrum, demonstrating an AQY of 3.75 ± 0.05% at 350 nm (5 mg of photocatalysts).*

Lines 443 – 445: *Notably, each reaction was repeated three times and averaged to ensure repeatability of data. The data in the graphs with error bars were expressed as the mean ± standard deviation of the sample population.*

Lines 446–452: *Since the conversion efficiency of reactant (MeOH and EG) in the system is lower than 1%, conducting a comprehensive quantitative analysis of consumed reactant and subsequently calculating the selectivity for D,L-LA is challenging. Instead, we evaluated the selectivity by calculating the ration of carbon in all products (D,L-LA, formate and glycolic acid). The carbon selectivity (C Sel.) of D,L-LA was calculated through the following equation:*

$$C\ Sel.(\%) = \frac{3 \times N_{(D,L\text{-}LA)}}{3 \times N_{(D,L\text{-}LA)} + N_{(formate)} + 2 \times N_{(glycolic\ acid)}} \times 100\%$$

(1)

where $N_{(D,L\text{-}LA)}$, $N_{(formate)}$, and $N_{(glycolic\ acid)}$ is the moles of produced D,L-LA, formate, and glycolic acid in the solution after the reaction.

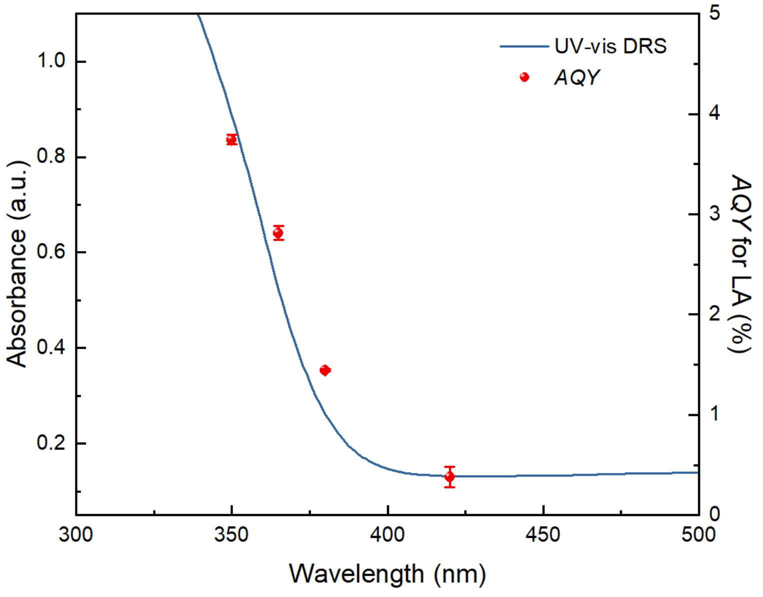

Fig. S15 *The apparent quantum yield (AQY) of Ba:TiO_2 sample. Catalysts mass: 5 mg. Light intensity: 22.9 mW cm^{-2} (350 nm); 26.5 mW cm^{-2} (365 nm); 28.5 mW cm^{-2} (380 nm); 29.6 mW cm^{-2} (420 nm). Irradiated area: 9.6 cm^2. A minimum of three replicate measurements was performed for each material group to ensure reproducibility ($n \geq 3$).*

Reviewer #3

In their manuscript, Wang et al. report a TiO_2-based photocatalysts for the joint production of lactic acid and hydrogen. While the activity of their most active Ba-TiO_2 material is very encouraging, some of the more in-depth mechanistic analysis requires substantial clarification.

Q1: *Several SI figures are of poor image quality and barely readable (e.g. S6, S20). Please check all figures for readability and use higher resolution as needed.*

R1: We thank the reviewer for pointing out the issue with figure readability. Following this suggestion, we have replaced all low-resolution images in the *Supplementary Information*, including Figs. S6 and S20, with high-quality versions. All *SI* figures have now been checked to ensure clear visibility of labels, axes, and spectral features, enabling readers of ***Nature Communications*** to access the full technical information without difficulty.

Q2: *How exactly does Ba insert (interstitial, ...)?*

R2: We thank the reviewer for raising this important question regarding the incorporation mode of Ba. Our combined structural and spectroscopic data show that Ba exists in TiO_2 in two well-defined forms: (i) atomically dispersed Ba substituting Ti^{4+} sites in the bulk lattice, and (ii) a minor fraction of Ba present as surface-bound species. First, the systematic shift of the (101) diffraction peak toward lower angles and the enlarged lattice fringes observed by HRTEM (Figs. 2b–2d) both reflect lattice expansion. This behavior aligns with expectations when a larger Ba ion with a radius of 1.35 Å replaces a smaller Ti ion with a radius of 0.61 Å. The EXAFS spectra (Fig. 2h) further confirms this interpretation. Only one dominant Ba–O coordination is detected, without Ba–Ba scattering. Especially, the average coordination number of Ba–O bond is 5.88, approximate to the theoretical value (6) in the model with Ba substituting bulk Ti in a TiO_6 structure framework. It should be noted that the average coordination number (5.88) of Ba–O bond obtained EXAFS fitting is slightly lower than the theoretical value (6) in the model, attributed to the existence of partial Ba species in the form of surface Ba with a coordination number (5). Furthermore, the uniform Ba distribution across the particles, revealed by XPS depth profiling and HAADF-TEM/EDS mapping (Figs. 2e, S7, and S8), further supports the uniform distribution of Ba species in the whole TiO_2.

Second, surface acidity/basicity measurements (CO_2-TPD, NH_3-TPD, and Py-IR; Figs. 5e and 5f) show that Ba introduction strengthens both Lewis acid and base sites. This behavior is consistent with surface frustrated Lewis pairs formed by electron-deficient Ba sites adjacent to lattice oxygen. In summary, substitutional Ba in the TiO_2 lattice improves charge separation, while the surface Ba-related species provide the complementary acid-base environment required for selective activation of the alcohol substrates and subsequent C–C coupling.

This dual contribution explains the enhanced photocatalytic performance observed for Ba-modified TiO_2.

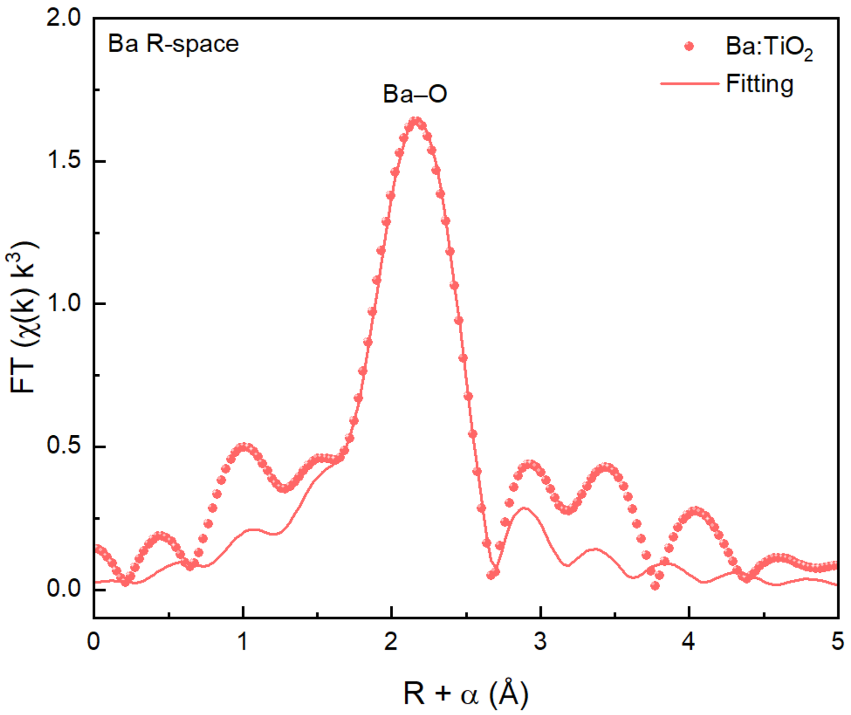

***Fig. 2h** Fourier transform of k^3-weighted EXAFS spectra at Ba K-edge of Ba:TiO_2.*

Q3: *The PL data (Fig. S19) has some datapoints indicated, but then more datapoints in between as suggested by the noise. Please clarify the true wavelength resolution.*

R3: We appreciate the reviewer's attention to the PL data. The wavelength resolution of PL is 1.0 nm. The original figure adopts the symbol-line format, leading to the missing of some data points. In the revised figure, all data points are shown in Fig. S26 (Fig. S19 in previous version).

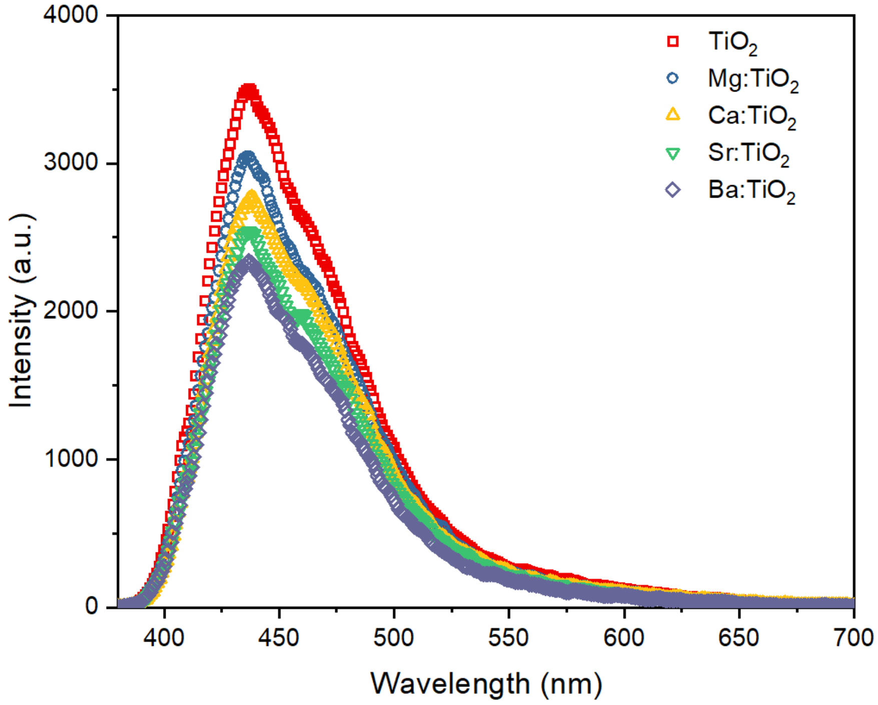

***Fig. S26** Steady state photoluminescence (PL) spectra of TiO_2 and M:TiO_2 samples (λ_{ex}: 350 nm).*

Q4: "the negative signal in Ba:TiO_2 undergoes a red shift over time, and its excited-state lifetime is significantly shorter than that of Ba:TiO_2" does not seem to make sense. Please clarify.

R4: We thank the reviewer for pointing out this inconsistency. The sentence in the original manuscript contained an obvious wording error. It has now been corrected to "*In contrast, the negative signal in Ba:TiO_2 undergoes a red shift over time, and its excited-state lifetime is significantly shorter than that of unmodified TiO_2*" (**Line 275**).

Q5: *The TAS signals in Fig. 4 are very small. What is the resolution limit of the used setup? How do the authors mitigate the risk of overinterpretation of transient features that are barely above the resolution limit?*

R5: We thank the reviewer for raising this point regarding the TAS signals. Although the signals in Fig. 4 appear small, they are well above the detection limit of the setup. The transient absorption spectrometer used in this study has a temporal resolution of approximately 800 fs and a noise floor of 2×10^{-4} ΔA under the applied conditions. The features in Fig. 4 have amplitudes ranging from 1.2×10^{-3} to 3.8×10^{-3} ΔA, corresponding to signal-to-noise ratios of 5–15, which are clearly above the instrumental threshold. Besides, several precautions were taken to avoid overinterpretation of weak features in this work. All TAS data were averaged over 5–10 individual scans. Besides, the lifetimes were obtained through multi-wavelength global fitting rather than relying on single-wavelength traces. Moreover, the TAS observations are also consistent with PL quenching, TRPL lifetime extension, and DOS analysis, providing mechanistic agreement across multiple techniques. These measures ensure that the transient features reported in Fig. 4 are robust, well above the resolution limit.

Q6: *Also on TAS: what is the feature seen around 490 nm? If excitation was done at 350 nm, it cannot be excitation scatter.*

R6: We thank the reviewer for pointing this out. The narrow feature around 490 nm does not originate from sample-derived transient species, but rather from a known artifact associated with the broadband probe continuum. The HELIOS pump-probe system operates with a broadband probe spanning 350–950 nm, as stated in the manufacturer's documentation. Such a wide spectral window is typically produced by white-light supercontinuum generation, which is known to introduce wavelength-dependent intensity modulations, residual chirp, and local spectral structure in the blue-green region. Such artifacts are commonly observed in HELIOS-type probe continua, appearing as wavelength-localized, time-independent features (***Appl. Phys.***, 2024, 130, 146). In the present work, the 490 nm feature exhibits negligible evolution with pump-probe delay. Besides, a similar feature appears in probe-only (unpumped) baseline scans, which is not included in the global

kinetic fitting. This has been mentioned in the revised manuscript.

Lines 276–277: *Notably, the sharp negative feature around 490–500 nm is attributed to a known artifact of the supercontinuum probe light source, rather than a sample-derived scattering signal.*

Q7: The statement "In general, species A represents the initial excited state or thermal carriers, while species B corresponds to the charge-separated state after relaxation" seems hard to justify, given that the component spectra (Fig. 4F) are very different between TiO_2 and Ba-TiO_2. As such, it seems unlikely that both components report on the same species in both materials.

R7: We thank the reviewer for this insightful comment. To avoid this misinterpretation, the revised manuscript clarifies that the components obtained from the global analysis in Fig. 4f correspond to kinetic components, meaning distinct dynamical processes rather than identical electronic states across different materials. In pump-probe spectroscopy of metal oxides, it is well established that the global fitting components often correspond to classes of carrier dynamic, such as ultrafast thermalized carriers, and longer-live trapped or charge-separated carriers, while the exact spectral profile of each component may vary from one material to another due to differences in trap-state distribution, local potential, band-edge structure, and recombination channels (***Nat. Catal.***, 2023, 6, 574–584; ***Nat. Chem.***, 2025, 17, 1348–1355). Despite these spectral differences, the roles of the two components within the relaxation process remain comparable between the two samples. The fast component (component A) appears immediately after excitation and decays on the sub-picosecond to few-picosecond scale, consistent with hot-carrier cooling and ultrafast trapping. The slower component (component B) reflects longer-lived carriers stabilized by trap states or partial charge separation. In Ba:TiO_2, its spectral characteristics and lifetime are altered by Ba-induced interstitial states, resulting in a distinct component B compared to pristine TiO_2. Therefore, the global fitting in the present work describes the carrier-dynamic pathways rather than identical electronic species.

To address the reviewer's concern and avoid any ambiguity, the term "species A/B" is replaced with "kinetic component A/B" in the revised manuscript. Besides, we explicitly explain that these components represent relaxation pathways instead of chemically identical excited states in different materials.

Lines 278–289: *To analyze the data further, a sequential model was used to perform a global fitting on the samples, obtaining the species-associated spectra (SAS) shown in Fig. 4f. The kinetic data for the selected wavelengths at 450 nm and 550 nm are displayed in Fig. S27, the fitted curves closely match the raw data, indicating the high-quality global fitting. The SAS spectra reveal that both TiO_2 and Ba:TiO_2 exhibit two kinetic components within the measurement time windows. After photo-excitation, the kinetic component A of TiO_2 forms instantaneously, manifesting as a broad negative signal between 400–700 nm. Kinetic component A transitions to kinetic component B on a slower timescale (166.5 ps), accompanied by a synchronous*

decrease in spectral intensity without any peak position shifts (Fig. S28a). A new kinetic component signal emerges at 400–480 nm, indicating the generation of a new excited state. Subsequently, kinetic component B undergoes complete relaxation at 1.8 ns. In contrast, after excitation in Ba:TiO_2, kinetic component A forms instantaneously, exhibiting two kinetic component signals at 400–450 and 600–700 nm, along with a negative signal at 450–600 nm. It then transitions to kinetic component B at a faster time scale (2.2 ps), where the negative signal intensifies and undergoes a red shift. Subsequently, kinetic component B fully decays at 984.1 ps (Fig. S28b).

Q8: ***(a)*** *How did the authors determine that 2 components should be used for the TA global analysis?* ***(b)*** *Does the fit quality improvement over a 1-parameter-fit justify the addition of an additional parameter, especially given the noise level of the data? Fit criteria such as AICc (Corrected Akaike Information Criterion) or BIC (Bayesian Information Criterion) would be useful to support this choice.*

R8: We appreciate the reviewer's insightful question regarding model selection in the global analysis.

(a) The decision to use two kinetic components was guided by both the physical behavior of the transient spectrum and a quantitative comparison of model quality. Inspection of the raw TAS data shows that the spectral evolution cannot be captured by a single decay component. In TiO_2, the broad early-time bleaching signal progressively narrows, which is accompanied by the rise of a new excited-state absorption band in the 400–480 nm region. This behavior reflects a transition from an ultrafast, thermalized carrier population to a more stabilized relaxation process. In Ba:TiO_2, the changes are even more pronounced, with a clear red-shift and strengthening of the negative signal that occurs on a distinct timescale. These features require at least two dynamical processes to describe the temporal and spectral evolution. In contrast, Imposing a single component would compress these changes into one exponential term, which fails to represent the underlying photo-physics.

(b) In accordance with the reviewer's suggestion, we conducted a quantitative evaluation to determine whether the introduction of a second component is statistically justifiable. Corrected Akaike Information Criterion (AICc) and Bayesian Information Criterion (BIC) values were calculated for both the one-component and two-component sequential models. The two-component model yields substantially lower AICs and BIC values, with ΔAICc values of –52.89 for TiO_2 and –56.91 for Ba:TiO_2. These differences far exceed the threshold, typically regarded as decisive evidence for preferring a more complex model. Both the clear temporal-spectral evolution in the experimental data and the statistically significant improvement in model fit strongly support the use of a two-component global analysis. The two-parameter fitting provides a balanced and conservative interpretation of the TAS dynamic without overfitting.

Lines 487–488: *The number of components in the sequential model was justified by a significant*

improvement in the fit quality, as quantitatively assessed by the Corrected Akaike Information Criterion.

Table R1. Comparison of one- and two-component kinetic models for global analysis of transient absorption data

	model	AICc	BIC	ΔAICc*	RSS (x 10^{-7})
TiO_2	one-component	–1468.84	–1461.31	n.a.	1.488
TiO_2	two-component	–1521.73	–1509.17	–52.89	0.743
Ba:TiO_2	one-component	–1435.95	–1428.42	n.a.	2.655
Ba:TiO_2	two-component	–1492.86	–1480.30	–56.91	1.228

* ΔAICc relative to one-component model

Q9: *Incomplete sentence: "Structurally, the conversion of methanol and ethylene glycol to lactic acid inherently involves the."*

R9: We appreciate the reviewer for pointing out the incomplete sentence. The text has now been corrected to ensure grammatical completeness and clearer scientific meaning. In the revised manuscript, the sentence has been updated to: "*From a structural standpoint, the conversion of methanol and ethylene glycol inherently requires the cleavage of both C–H and C–O bonds*" (**Line 58**).

Q10: *What are the apparent quantum yields (AQYs) for the LA and H_2 production reactions? Please report for at least one wavelength.*

R10: We thank the reviewer for this valuable suggestion. The authors have now carried out the *AQY* measurements as requested. The corresponding experimental details and the *AQY* values at a representative excitation wavelength have been incorporated into the revised manuscript.

Lines 195–197: *The photocatalytic coupling performance of Ba:TiO_2 was further tested by the action spectra, namely the apparent quantum yield (AQY). As shown in Fig. S15, the AQY (for the synthesis of LA) of Ba:TiO_2 exhibits a positive correlation with its UV–vis DRS spectrum, demonstrating an AQY of 3.75 ± 0.05% at 350 nm.*

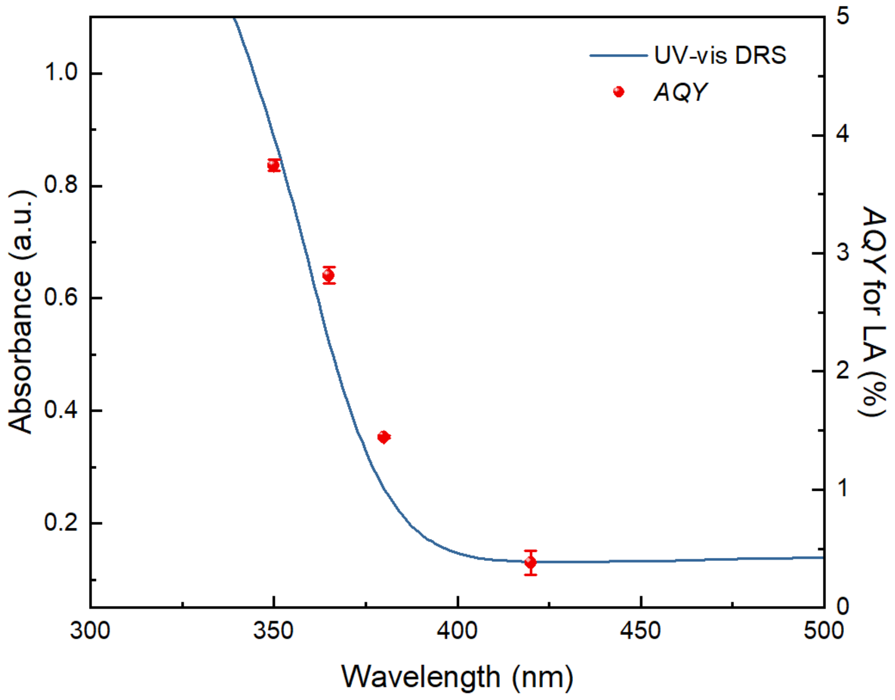

***Fig. S15** The apparent quantum yield (AQY) of Ba:TiO_2 sample, catalysts: 5 mg. The apparent quantum yield (AQY) of Ba:TiO_2 sample. Catalysts mass: 5 mg. Light intensity: 22.9 mW cm^{-2} (350 nm); 26.5 mW cm^{-2} (365 nm); 28.5 mW cm^{-2} (380 nm); 29.6 mW cm^{-2} (420 nm). Irradiated area: 9.6 cm^2. A minimum of three replicate measurements was performed for each material group to ensure reproducibility (n ⩾ 3).*

Q11: *Did the authors consider non-surface-Ba acting as hole trap, given that Ba is distributed throughout the entire material? The effects of bulk Ba on the optical measurements conducted should be mentioned.*

R11: Thank you for your comments. DOS results show that most Ba 6*p* states in TiO_2 bulk are distributed slightly above the valence-band top (Fig. 1d), which produce little effect on the band-gap value of TiO_2 (Figs. S22–S25). However, Fermi level is directly localized in the region of Ba 6*p* states, implying that Ba site can directly capture electrons from valence band for promoting photoexcitation and charge separation. This result is consistent with the in-situ XPS measurements in which Ba species, including bulk Ba species, accumulate photogenerated electrons under illumination (Fig. 4g).

In response to the reviewer's suggestion, we have additionally strengthened the discussion on the influence of bulk Ba on the optical properties in the revised manuscript, as detailed below:

(Lines 117-120) *A band structure analysis showed that only the Ba species can generate strong interstitial states slightly above valence band (Fig. 1d). Therefore, Ba doping produces little effect on the band gap of TiO2. Additionally, Fermi level is directly localized in the region of Ba 6p states, implying that Ba site can effectively capture electrons from valence band for promoting charge separation.*

Q12: *The claimed single atom nature of the Ba sites is not demonstrated convincingly - the relatively blurry*

images in Fig. 2 and insufficient to fully support this claim. I recommend the authors conduct experiments that could demonstrate the single atom nature (e.g. EXAFS/XANES) or leave open the possibility that the Ba sites may be small Ba clusters rather than single atoms.

R12: Following the reviewer's thoughtful suggestions, the author has strengthened the evidence for the atomic distribution of Ba species in TiO_2 by EXAFS/XANES. All revisions are highlighted in yellow.

Lines 154–160: *The aberration-corrected scanning transmission electron microscopy (AC-STEM) reveals that disc-like Ba:TiO_2 is nanoporous, and bright dots on the TiO_2 matrix are assigned to Ba single atoms (Fig. 2f). These nanopores with around 1–3 nm originated from the non-collapsed pore structure of MIL-125(Ti) (Fig. S9). The coordination environment of Ba species in Ba:TiO_2 were further investigated by Ba K-edge XANES and EXAFS analysis (Figs. S10 and S11). The k^3-weighted Fourier-transformed EXAFS spectra show that Ba:TiO_2 only has a dominant peak at ~ 2.2 Å, assigned to Ba–O bond (Fig. 2h). Especially, the fitting results of EXAFS further confirms the atomically dispersed nature of Ba coordinated with six O atoms, consistent with the above theoretical model.*

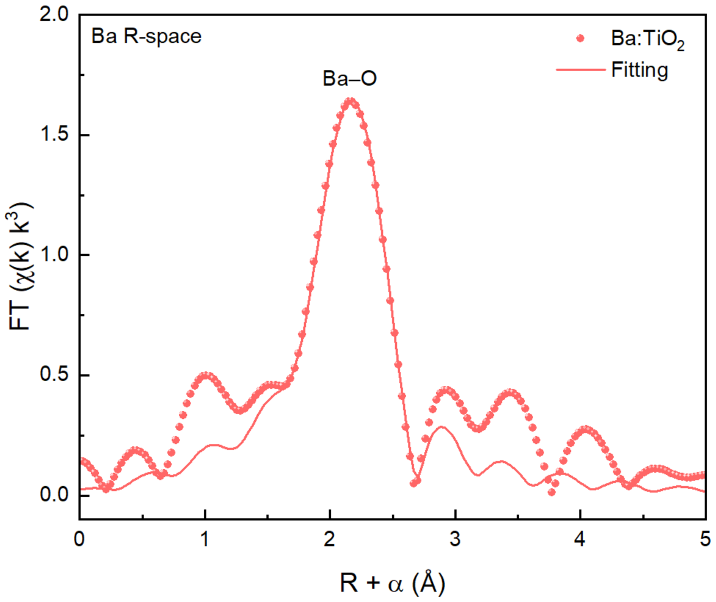

***Fig. 2h** Fourier transform of κ^3-weighted EXAFS spectra at Ba K-edge of Ba:TiO_2.*

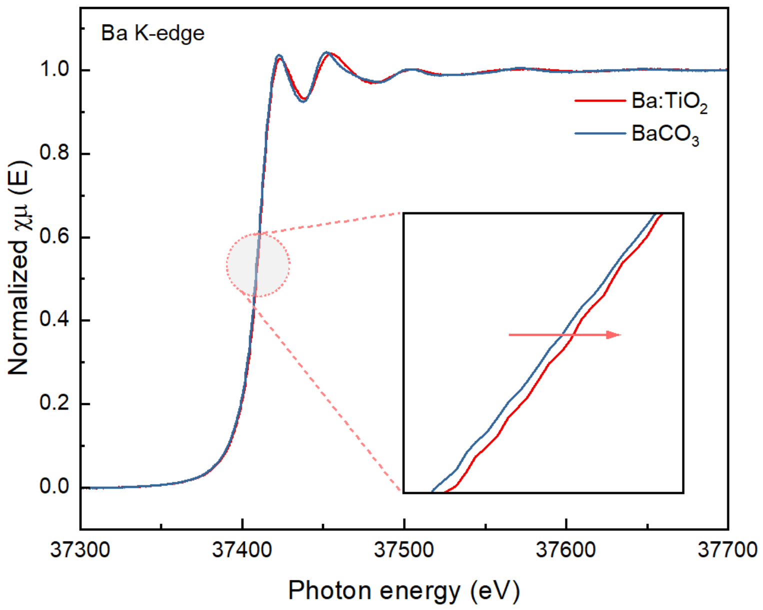

Fig. S9 XANES spectra of Ba K-edge for Ba:TiO₂ and BaCO₃.

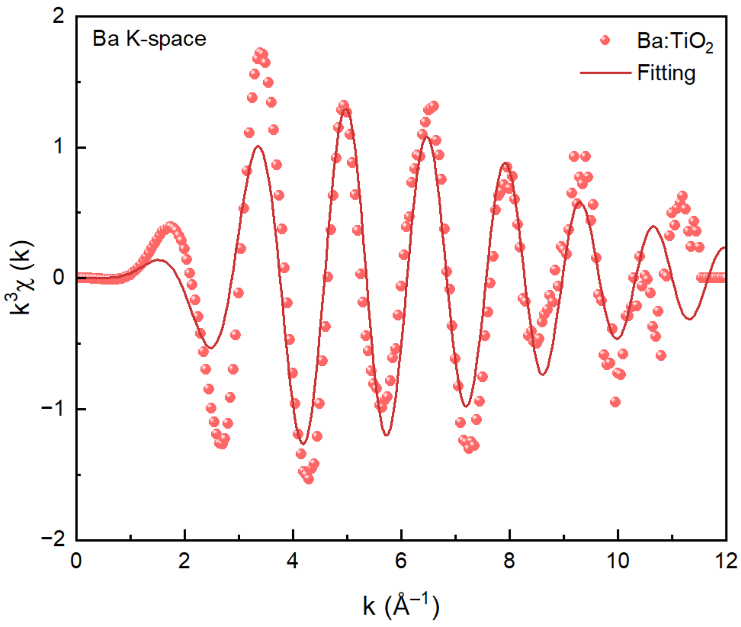

Fig. S10 *Ba K-edge EXAFS (points) and fitting curve (line) for Ba:TiO₂, shown in κ^3-weighted K-space.*

Q13: *The PL spectra presented in Fig. 4 in the main text are much redshifted compared to those in Fig. S19. Why?*

R13: We thank the reviewer for raising this point. The perceived redshift does not reflect any intrinsic spectral change of the materials. Instead, it originates solely from the use of different measurement modes and instruments for the two PL datasets. The temperature-dependent PL spectra shown in Fig. 4b and Fig. 4c were acquired using a cryostat-coupled PL setup, which employs the distinct excitation geometry, grating configuration, detector response profile, and optical filtering. In contrast, the spectra in Fig. S26 (revised from Fig. S19) were collected using a conventional steady-state PL spectrometer. These instrumental differences

lead to systematic shifts in the detected emission profiles.

Q14: *The PL spectra shown in Fig. S4 lie relatively far from the bandgap energy. The authors state that they investigate the exciton binding energy via the temperature dependence, however, this is only the case if emission occurs from the band state. In this case, the PL occurs relatively far from the bandgap energy, and is thus more likely investigating PL related to trap states; as such, it is unlikely that the Arrhenius activation energies are reflective of the excitation binding energy. This suggests that the statement "indicate that the alkaline earth metal introduction can minimize the* E_b*" is unsubstantiated.*

R14: We thank the reviewer for this insightful comment. We agree that the temperature-dependent PL emission shown in Figs. 4b and 4c lie well below the bandgap energy of TiO_2, indicating that the dominant radiative channel arises from sub-bandgap trap states (including Ba 6*p*) rather than band-edge excitonic recombination. As the reviewer correctly notes, Arrhenius' analysis of such trap-related PL cannot be used to determine the intrinsic exciton binding energy. The manuscript has been revised to remove any reference on exciton binding energy from these measurements.

Lines 260–269: *Besides, as shown in Figs. 4b and 4c, the thermal activation energy for charge carrier release was investigated via the temperature-dependent PL spectra. As the temperature increased from 77 to 350 K, the integrated PL intensity of* TiO_2 *and Ba:*TiO_2 *decreased monotonically, which can be mainly attributed to the thermally activated non-radiative recombination process. Given that the PL emission occurs at energies significantly below the bandgap, the signal likely originates from trap states rather than direct band-edge recombination. By fitting the experimental data with the Arrhenius equation, the thermal activation energies of* TiO_2 *and Ba:*TiO_2 *were determined to be 47.82 and 35.24 meV, respectively. This lower activation energy in Ba:*TiO_2 *indicates a reduced barrier for charge carrier release from trap states and subsequent migration, contributing to less energy loss in the charge migration process. These results demonstrate that the alkaline earth metal introduction can reduce the effective thermal activation barrier, which facilitates the photogenerated carrier generation and transport in* TiO_2*.*

Q15: *"Through in-situ XPS spectra analysis of Ba:*TiO_2*, it was determined that under illumination, the photo-generated electrons migrate toward Ti and Ba metal sites while holes migrate toward lattice oxygen sites": it seems the main differences are between* TiO_2 *and Ba-*TiO_2*, not Ba-*TiO_2 *in the dark vs under illumination. Therefore, it is unclear where this conclusion comes from. Please clarify.*

R15: The authors thank the reviewer for this thoughtful comment. The conclusion that photogenerated electrons migrate toward Ti or Ba sites and holes migrate toward lattice oxygen is drawn from the comparison between Ba-TiO_2 in the dark and under illumination (Fig. 4g). The comparison of electronic properties of

unmodified TiO_2 and Ba:TiO_2 is performed under dark conditions (Fig. S13). To avoid misunderstanding, the XPS of unmodified TiO_2 under dark conditions is moved to supporting information.

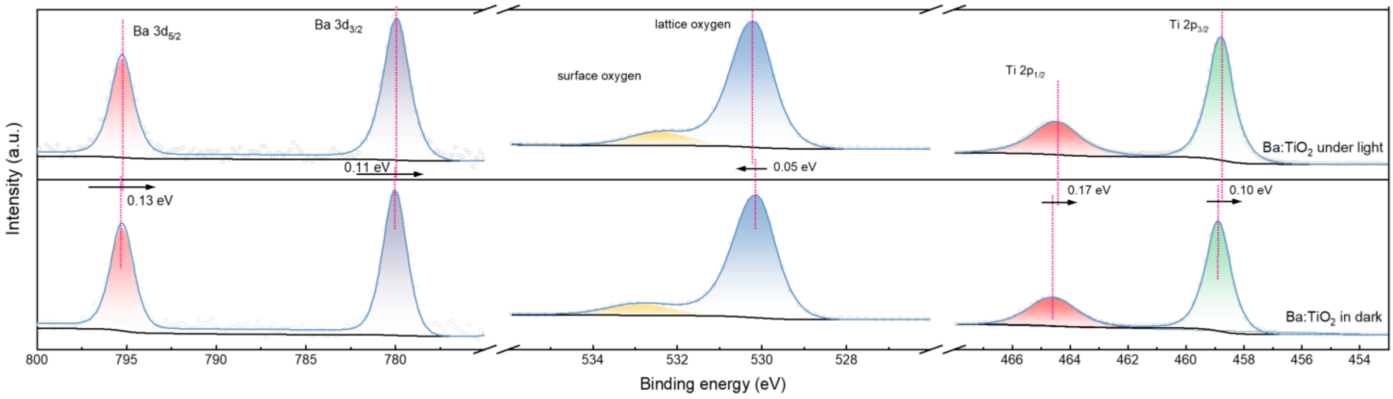

Fig. 4g *In-situ XPS spectra of Ba:TiO_2 under illumination for 15 min.*

Q16: *The authors address the "depletion of C–OH bond" in Fig. 5. What is the signature of this depletion? It seems there are only signals that increase over time, not decrease - at most a constant amplitude around 1110 cm^{-1}.*

R16: The authors thank the reviewer for this helpful comment. The authors agree that, in the originally presented plotting style, the evolution of the band near 1100 cm^{-1} may not have appeared fully intuitive. To examine this point more clearly, the authors re-plotted the *in-situ* ATR data, as shown in Fig. R1. Upon close inspection of the spectral region around 1100 cm^{-1}, a distinct decrease in the intensity of the negative-going signal is evident as the reaction proceeds from 1 to 15 min. This negative-going feature is attributed to the consumption of the C–OH group in ethylene glycol, and its diminishing intensity constitutes the direct spectroscopic signature of C–OH bond depletion.

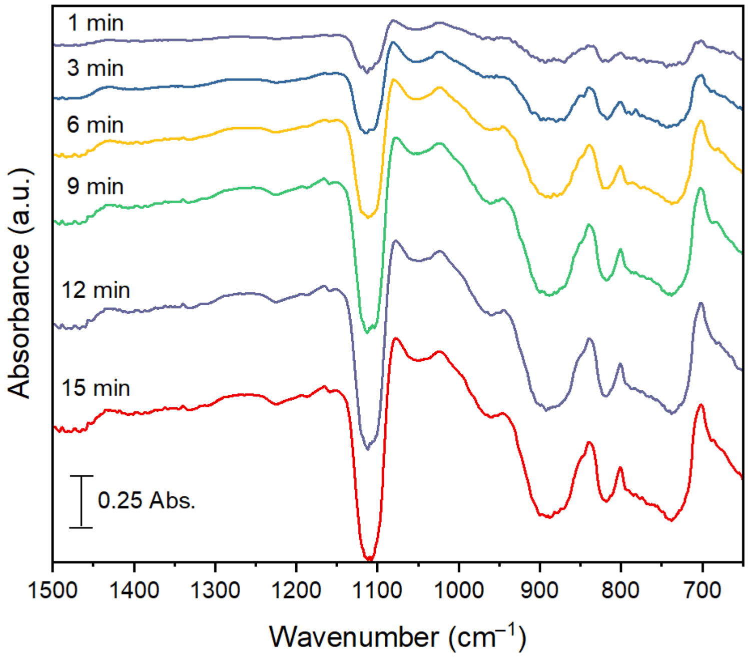

Fig. R1 *Time-dependent IR signals for the pristine TiO_2 under our reaction conditions.*

Q17: *While the poisoning experiments with pyrrole and pyridine imply cooperative acid–base involvement, an FLP requires acid and base sites to be adjacent in order to operate as a paired site under reaction conditions. So far, the authors do not demonstrate this spatial proximity - please clarify.*

R17: We thank the reviewer for raising this important point. The authors agree that spatial proximity between the Lewis acid and Lewis base sites is essential for an FLP to operate. Firstly, the spatial proximity of FLP in Ba:TiO_2 is inherently promoted by the MOF-derived synthesis route. In the precursor MIL-125(Ti), Ba cations are incorporated into the periodic pores and confined at the molecular scale. During calcination, the MOF framework converts into nanoporous anatase TiO_2 while the pre-confined Ba species are fixed in place, preventing aggregation and maintaining atomically dispersed Ba within the TiO_2 matrix. This transformation ensures that each Ba site is formed adjacent to lattice oxygen atoms, creating naturally paired Lewis acid (Ba) and Lewis base (O^{2-}) sites. Secondly, microscopy and spectroscopy provide strong evidence for this spatial arrangement. HAADF-TEM and EDS mapping show a uniform nanoscale distribution of Ba, Ti, and O without any Ba-rich domains. EXAFS analysis further confirms that Ba exists exclusively as isolated atoms without Ba–Ba coordination paths, indicating that each Ba atom is embedded within the TiO_2 lattice and surrounded by oxygen atoms. Furthermore, DFT simulation reveals that the distance between paired Lewis acid (Ba) and Lewis base (O^{2-}) sites is 2.42 Å, which can enable the Ba and O sites simultaneously interacting with reactants. Such synergistic activation would not occur if the two sites were separated. Additionally, poisoning experiments also reinforce this interpretation. The complete loss of catalytic activity when either Ba sites (pyridine) or lattice oxygen sites (pyrrole) are blocked suggests that both sites must work together. Therefore, these combined synthetic, structural, computational, and functional results support the conclusion that the adjacent Lewis acid–base pairs on Ba:TiO_2 are capable of FLP-like cooperative activation ability under reaction conditions. We have revised the discussion in for strengthening the logical connection:

Lines 376–381: *As shown in Fig. 5g, the addition of either pyrrole or pyridine completely quenched the photocatalytic activity. Given the atomically dispersed nature of Ba (Figs. 2e–2h), it can be inferred that each Ba Lewis acid site is coordinatively surrounded by multiple lattice oxygen Lewis base sites (analogous to the host TiO_2 crystal structure). This intrinsic spatial proximity, enforced by the host lattice, allows the Ba–O pairs with a length of 2.42 Å to function cooperatively in the simultaneous activation of distinct bonds in the reactants, as evidenced by our DFT calculations (Figs. 1a and 1b).*

Reviewer #4 (Remarks to the Author):

The manuscript reports a series of alkaline earth metal doped TiO_2 (M-TiO_2) photocatalysts for photocatalytic upcycling of ethylene glycol to C_3 compounds. The manuscript presents a combination of experimental and computational studies to rationalize the claim that the implantation of alkaline earth metal cations promotes both selectivity and activity. However, the current results are superficial. Especially, some key results are self-contradicted (see my comments below). Therefore, I cannot recommend the publication of this work in Nature Communications.

1. Major issues in catalytic performance

Q1: **(a)** *As reported by the authors, Ba-TiO_2 shows a production rate of LA of 2.8 mmol g^{-1} h^{-1} (corresponding to 14 μmol h^{-1}, 5 mg catalysts used in the reaction) under a 360-mW cm^{-2} light irradiation, and meanwhile, demonstr*ates a AQY of 3.*75% at 350 nm (light intensity of 22.9 mW cm^{-2}). When taking the AQY value into the Eq. S1, the production rate of LA under 350-nm light irradiation can be calculated to be 22 μmol h^{-1}, even much higher than that obtained under normal conditions. This is unreasonable.* **(b)** *Furthermore, even at 420 nm where Ba-TiO_2 basically has no light absorption, the AQY still shows to be around 0.5%. The AQY values seem to be heavily overestimated in the study.*

R1: We appreciate the questions raised by the reviewers.

(a) We would like to clarify that the photocatalytic activity tests and the *AQY* measurements were performed under **two different Xe lamps**. The activity tests were conducted under broadband light irradiation (**360 mW cm^{-2}**), whereas the *AQY* measurements were carried out using monochromatic light obtained through **a single-pass band-pass filter** under another broadband light (**470 mW cm^{-2}**). As a result, **the effective photon flux (UV region), irradiation area, and spectral distribution** are not directly comparable between **two different Xe lamps**. Therefore, the *AQY* values are not intended to be used to quantitatively back-calculate the overall reaction rate under broadband illumination.

(b) Regarding the non-zero *AQY* observed at 420 nm (*ca.* 0.15%), we note that the band-pass filter used in the *AQY* measurements exhibits a finite spectral bandwidth, allowing a small fraction of photons in the 380–400 nm range to pass through (**Fig. R1**). This is s common phenomenon in *AQY* measurement (*Nat. Commun.*, 2025, 16, 6014; *Nat. Catal.*, 2025, 8, 728–739). This residual high-energy light can weakly excite Ba:TiO_2, leading to a small apparent *AQY* signal. We have removed the *AQY* at 420 nm from the revised manuscript to avoid potential misinterpretation (**Fig. S18**).

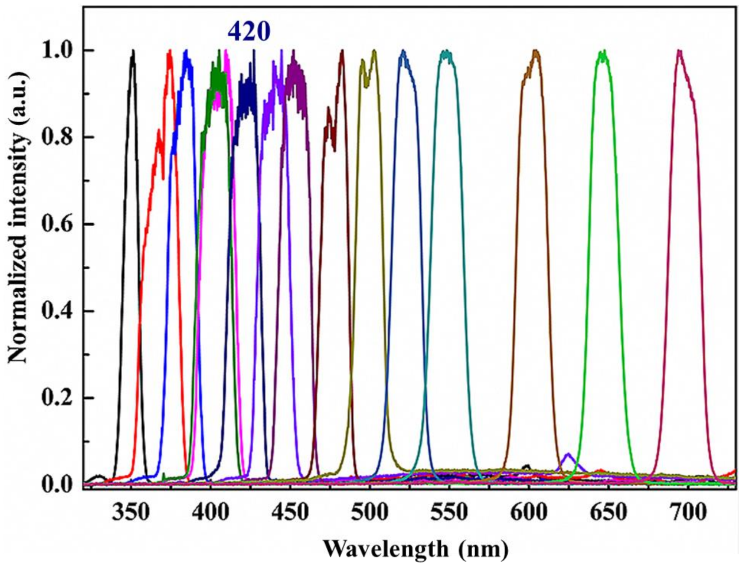

Fig. R1 UV－vis spectra of bandpass filters at different wavelengths.

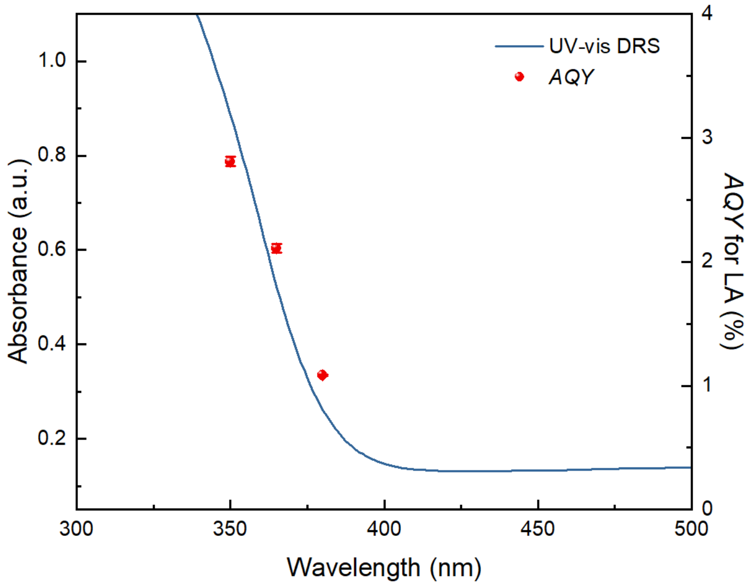

Fig. S18 *The apparent quantum yield (AQY) of Ba:TiO_2 sample. Catalysts mass: 5 mg. Light intensity: 22.9 mW cm^{-2} (350 nm); 26.5 mW cm^{-2} (365 nm); 28.5 mW cm^{-2} (380 nm). Irradiated area: 9.6 cm^2. A minimum of three replicate measurements was performed for each material group to ensure reproducibility ($n \geq 3$).*

Q2: *The authors plotted the standard curves of different products at concentrations ranging from 10 mM to 100 mM. However, according to the activity data listed in Table S2, the actual concentration of the products in the reaction solution was in the range of 0–2.1 mM, which deviated significantly from the concentration range of the standard curves. This situation would lead to significant error in quantitative analysis.*

R2: Thank you for your valuable comments. We have redrawn the series of standard curves with appropriate range based on the reviewer's suggestions and replaced them in the revised *Supporting Information*. Meanwhile, we recalibrated all performance test values in the revised manuscript based on the new standard curves.

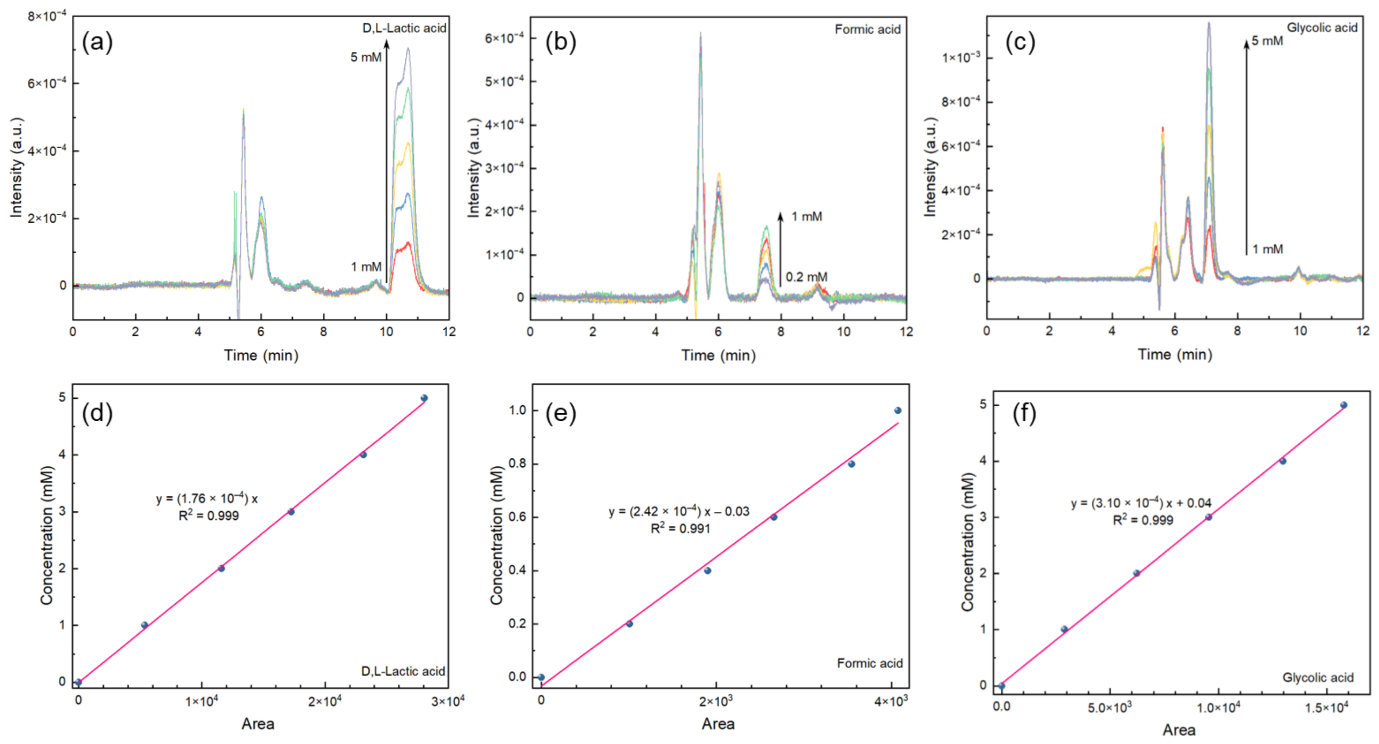

***Fig. S17** High-performance liquid chromatography (HPLC) of D,L-lactic, formic acid, and acid glycolic acid standard chemicals, with corresponding work curves.*

Q3: The HPLC spectra for all M-TiO_2 are lacking, which leads to the difficulty for reviewers to access the reliability of activity data.

R3: Thanks to the reviewer's comment. We have provided the HPLC spectra for M:TiO_2 in the revised *Supporting Information*. (Please see **Figs. S23–S25**)

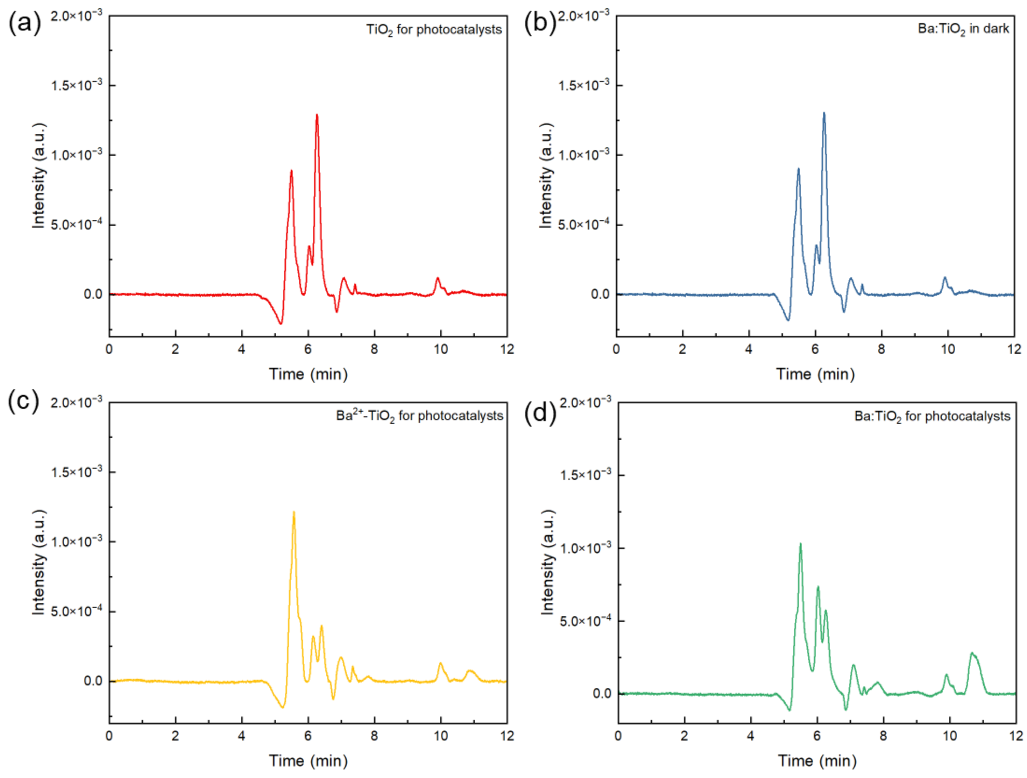

***Fig. S23** HPLC spectra of (a) TiO_2, (b) Ba:TiO_2 in dark, (c) Ba^{2+}-TiO_2, and (d) Ba:TiO_2, corresponding to*

Fig. 1b. *Minor invariant peaks observed at specific retention times are attributed to system-related background signals or trance impurities are not associated with photocatalytic reaction products.*

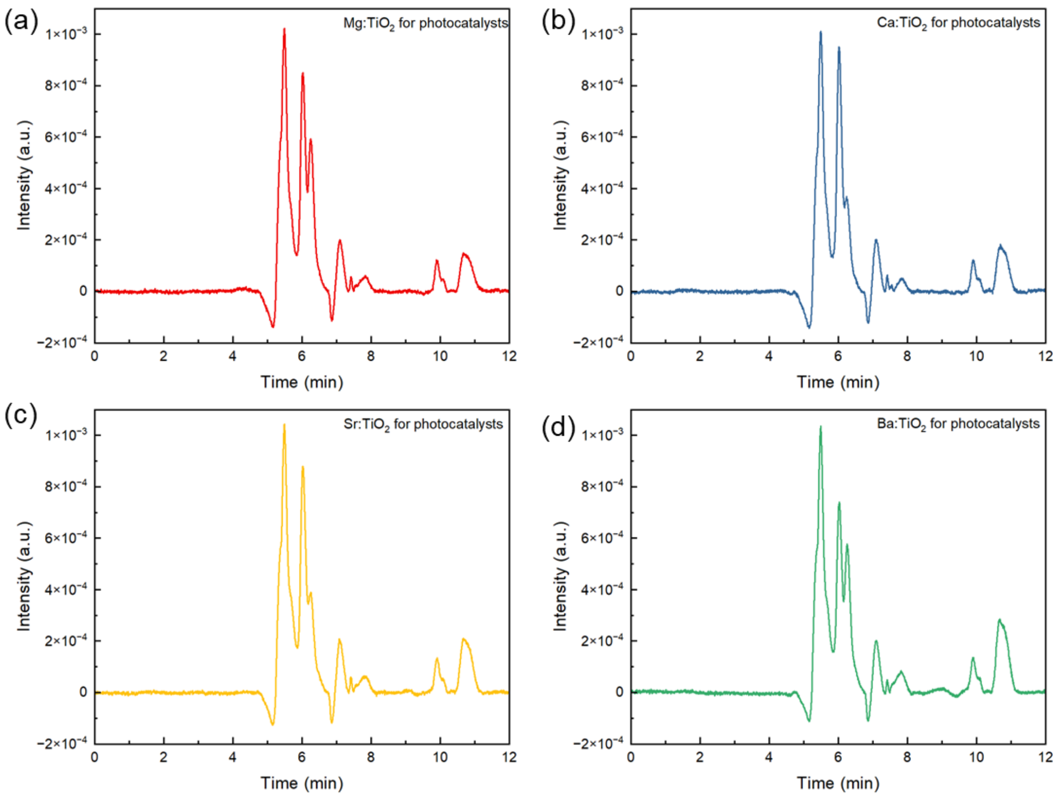

Fig. S24 *HPLC spectra of (a) Mg:TiO2, (b) Ca:TiO2, (c) Sr:TiO2, and (d) Ba:TiO2, corresponding to* ***Fig. 1c***.

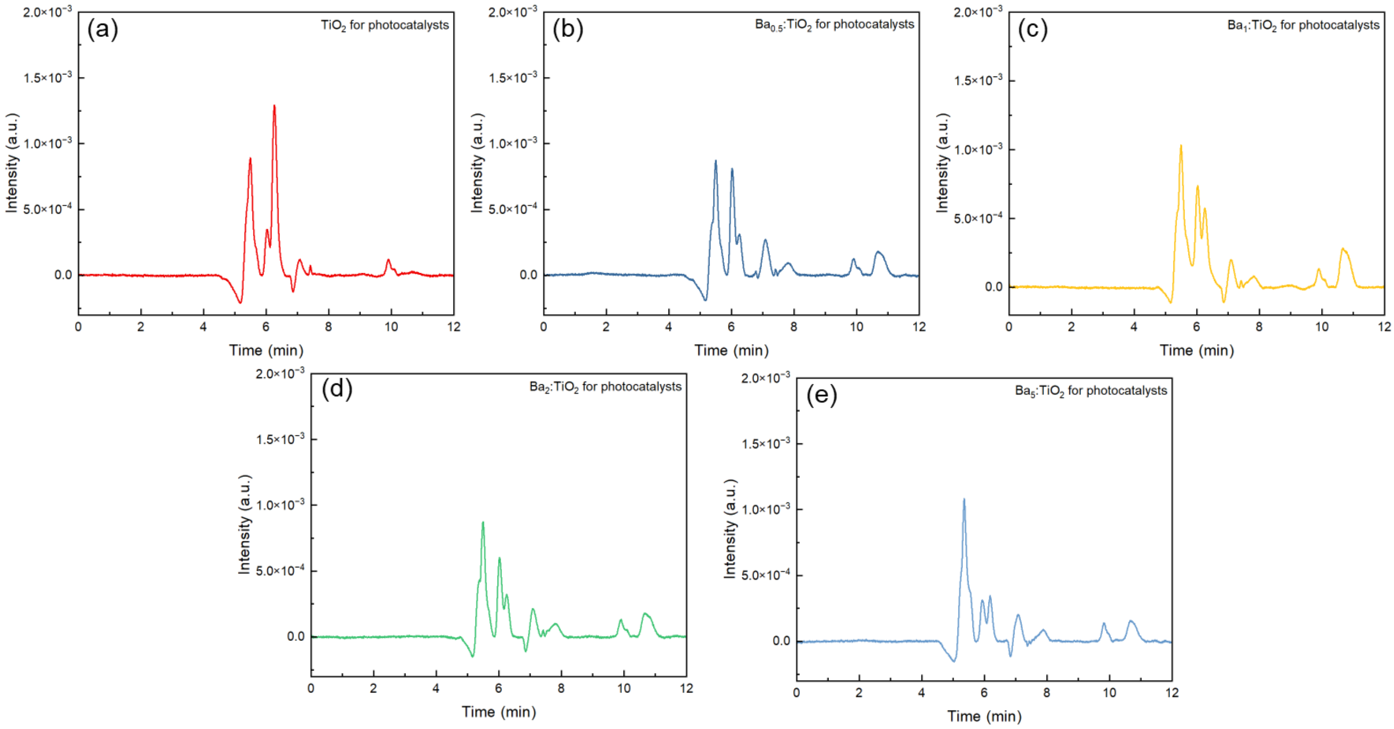

Fig. S25 *HPLC spectra of (a) TiO2, (b)Ba0.5:TiO2, (c) Ba1:TiO2, (d) Ba2:TiO2, and (d) Ba5:TiO2, corresponding to* ***Fig. S22***.

Q4: **(a)** *In Figure S32, the authors provide the HPLC spectra of* TiO_2 *recorded before and after reaction. A significant quantity of glycolic acid was detected before the reaction. Why?* **(b)** *The signals of lactic acid and formic acid are too weak to be analyzed quantically.* **(c)** *From what sources do the signals at 5.5, 6, 6.5, 9.8, and 10.2 min originate?* **(d)** *Moreover, why did these signals change after the reaction?*

R4: We thank the reviewer for carefully examining the HPLC data and for raising these important questions.

(a) The glycolic acid signal observed prior to the photocatalytic reaction originates from the weak background oxidation of ethylene glycol under strongly alkaline conditions. Such spontaneous and weak oxidation processes have been widely reported for polyol substrates in alkaline environments (*Chem Catal.*, 2021, 1, 941–955; *ChemElectroChem*, 2020, 7, 4326–4335). Additionally, as shown in **Fig. S23b**, the signal from glycolic acid remains detectable under dark conditions. Therefore, the presence of glycolic acid before reaction reflects a background chemical process rather than photocatalytic activity.

(b) Regarding lactic acid and formic acid, their signals are indeed weak in **Fig. S37**. However, the quantitative integration can be achieved after appropriate baseline correction, and the corresponding values are reported based on repeated measurements. Additionally, the low signals of lactic acid and formic acid cannot produce observable influence on the calculation of activity and selectivity.

(c) To clarify the origin of the additional peaks at 5.5, 6.0 and 6.5 min, we performed blank experiments under identical alkaline reaction conditions followed by the same neutralization and HPLC analysis procedure. These peaks are consistently observed in the blank samples, which can be attributed to system-related background signals arising from inorganic salts during pH adjustment, *e.g.*, sulfate species, rather than from organic reaction intermediates or products. The signals at 9.8 and 10.2 min are present with nearly identical retention times and integral areas in all samples, including blanks and post-reaction solution. Especially, their invariance before and after reaction indicates that they are unrelated to the photocatalytic process. We therefore attribute these peaks to system-level background signals associated with the HPLC setup, which can be excluded from the product analysis.

(d) After the reaction, slight shifts in retention time were observed for several signals. However, no new peaks emerged and the overall chromatographic profile remained consistent with that of the initial system. The minor retention time variations are likely associated with subtle changes in solution composition (*e.g.,* ionic strength or pH) rather than the formation of new chemical species.

We have clarified these points in the revised *Supporting Information* to avoid potential confusion regarding the origin and significance of these signals (**Fig. S23**).

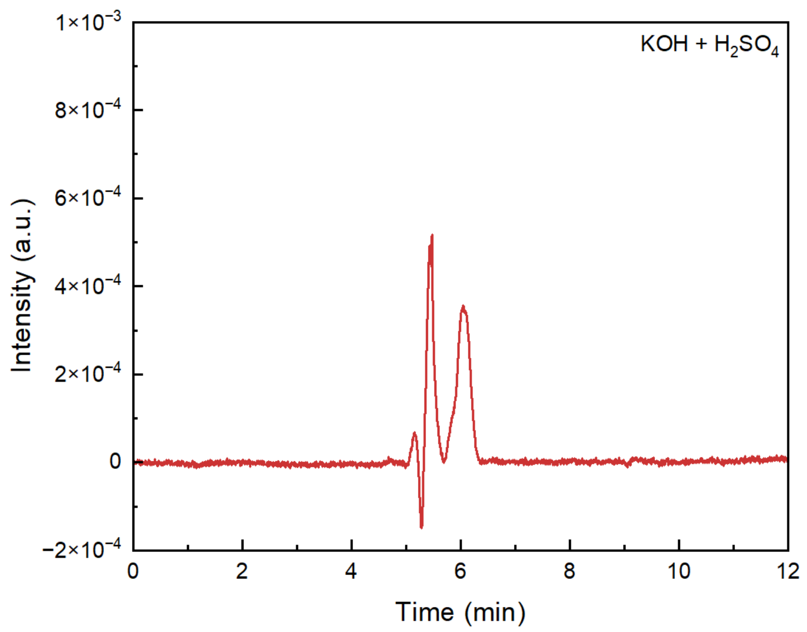

***Fig. R2** HPLC spectra of blank experiments under identical alkaline reaction conditions followed by the same neutralization.*

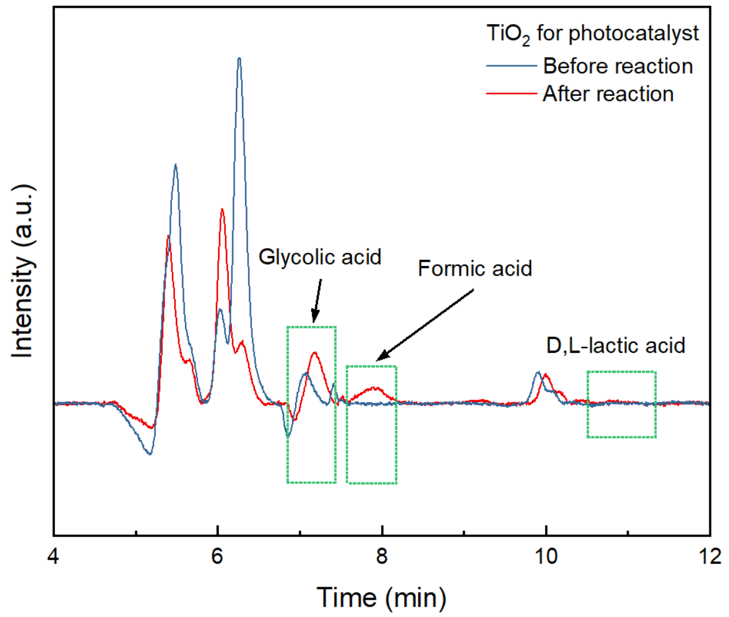

***Fig. S37** HPLC results of the photocatalytic C–C cross-coupling to produce LA system using TiO_2 as a catalyst after a 2-h reaction.*

Q5: *The authors subjectively attribute the activity loss in cycling measurements to catalyst loss. Therefore, to minimize catalyst loss, they developed a photocatalytic system composed of a continuous-flow reactor. However, they conducted only a single-time activity test using this system, and the stability of this system was not investigated. Moreover, the original HPLC spectrum data is not given.*

R5: We thank the reviewer for this constructive comment. We agree that attributing the activity loss during cyclic testing solely to catalyst mass loss was not sufficiently supported. In the revised manuscript, we have

moderated this statement and clarified that multiple factors, including catalyst deactivation (Figure 3e), may contribute to be observed decline in activity. It is not strange that any catalyst faces the deactivation issue in the long-term test. The continuous-flow photocatalytic system was mainly designed as a proof-of-concept to demonstrate the feasibility of catalyst integration into a device configuration using natural sunlight, rather than evaluating long-term stability. We have revised the manuscript to explicitly clarify this point and avoid any implication of stability assessment. In addition, the original HPLC spectra obtained from the outdoor testing of the continuous-flow system have now been provided in the *Supporting Information* to ensure data transparency.

Lines 249–258: *The reducibility and stability are critical parameter for practical applications. Inductively coupled plasma analysis confirms that the Ba content in Ba:TiO_2 remains essentially unchanged after a 12-hour reaction (0.91 wt%) compared to the initial value (0.92 wt%), indicating negligible Ba leaching and compositional variation during operation. In cycling measurement, Ba:TiO_2 maintains steady product selectivity over 10 consecutive runs (Fig. 3e), although a gradual decrease in activity is observed. The activity loss may partially originate from catalyst mass loss during centrifugal recovery, while surface deactivation of Ba:TiO_2 cannot be excluded. To conduct a proof-of-concept demonstration of photocatalytic device integration, we developed a tandem photocatalytic system composed of a continuous-flow reactor using natural sunlight. The Ba:TiO_2 powder was immobilized in the continuous-flow reactor for producing LA (Fig. 3f), which avoids the separation of catalyst and reaction solution.*

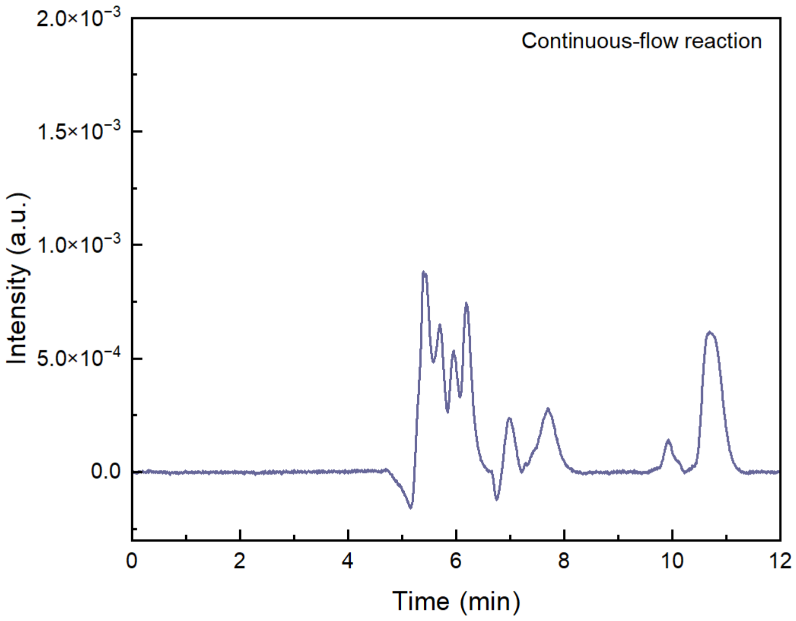

Fig. S28 *Continuous-flow reaction under a full day of outdoor sunlight exposure (from 9:00 to 17:00), collected at 22°35'49" N, 113°58'22" E, and on Aug 10, 2025.*

2. Major issues in theoretical calculations

Q6: *The study performed the theoretical calculations on the free energy change in the formation pathway of lactic acid over TiO_2 and M-TiO_2. The results seem to explain the higher activity of M-TiO_2 than TiO_2. However, the calculations on the pathways of other products are lacking. For example, CH_3OH to HCHO, CH_3OH to HCOOH, CH_3OH to ethylene glycol and ethylene glycol to glycolic acid. These reactions compete with the formation of lactic acid. comparing these pathways, the conclusion of this section, which the authors claimed as "the activity and selectivity for LA production on M-TiO_2 are greatly enhanced, particularly with the introduction of Ba", cannot be drawn.*

R6: Thank you for your valuable comments. Considering LA as the main product, the calculation in the original manuscript was primarily used to compare the LA-production rates of TiO_2 and Ba:TiO_2. It should be noted that the higher selectivity of LA on Ba:TiO_2 is attributed to the higher LA-production rate of Ba:TiO_2 instead of the reduced byproducts-production rate since the production rate of byproducts (HCOOH and glycolic acid) are approximate on TiO_2 and Ba:TiO_2. Additionally, we calculated the free energy profiles of HCOOH and glycolic acid formation on Ba:TiO_2 in the revised manuscript. The results obtained show that the generation process of HCOOH and glycolic acid need to overcome two high-barrier reaction steps with reaction energy of higher than 1 eV. However, apart from the rate-determining step, other reaction steps in the generation process of LA over Ba:TiO_2 exhibit reaction energies lower than 0.3 eV. (Note: if two reaction pathways have a similar energy barrier, such as 1.36 eV in the formation of LA and 1.39 eV in the formation of HCOOH, we need to compare the secondary energy barriers, such as 0.31 eV in the formation of LA and 1.05 eV in the formation of HCOOH). The above results indicate that Ba:TiO_2 reduces the energy barrier for the formation of LA. Meanwhile, it does not lead to a lower formation energy barrier for HCOOH and glycolic acid, thereby leading to the excellent activity and selectivity of Ba:TiO_2. We have added above discussions into the revised manuscript.

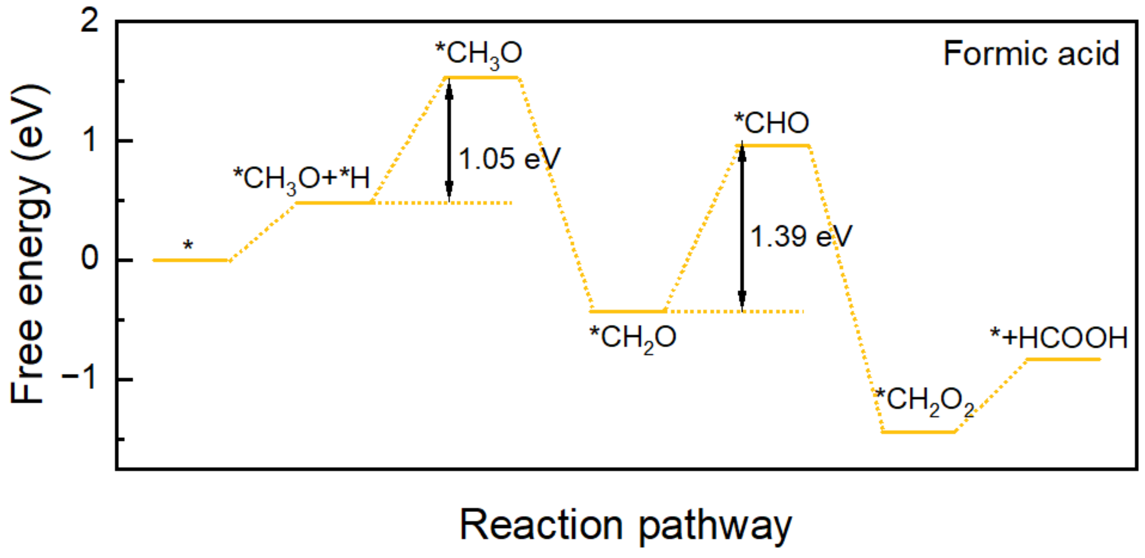

Fig. S4 *Free energy profile of formic acid production on Ba:TiO_2 at pH = 7 and U = 0 V vs. SHE.*

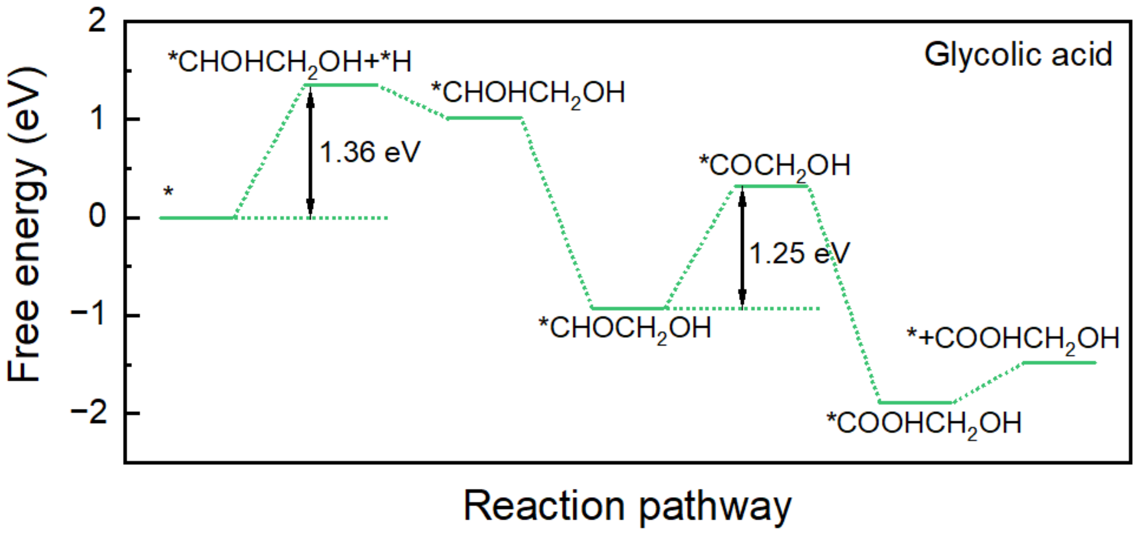

Fig. S5 *Free energy profile of glycolic acid production on Ba:TiO2 at pH = 7 and U = 0 V vs. SHE.*

Lines 118–126: *Additionally, to evaluate the competing production of some byproducts from non-C–C coupling reaction, the free energy profiles of generating formic acid from methanol and glycolic acid from ethylene glycol were also calculated on Ba:TiO2 (Figs. S4 and S5). The results obtained show that the generation processes of formic acid and glycolic acid have to overcome two high-barrier reaction steps with reaction energy of higher than 1 eV. However, apart from the rate-determining step, other reaction steps in the generation process of LA over Ba:TiO2 exhibit reaction energies lower than 0.3 eV, suggesting that the generation of LA is prior to those of formic acid and glycolic acid over Ba:TiO2. Therefore, the addition of Ba cannot only theoretically improve the generation rate of LA but also endows TiO2 with a high selectivity on the targeted LA rather than formic acid or glycolic acid.*

Q7: *According to the DOS calculations in Figure 1d, it can be seen that barium implantation create interstitial states near the valence band of TiO2. This finding raises two questions.* **(a)** *Why does Ba-TiO2 show an increased bandgap?* **(b)** *The interstitial states from Ba prefer to act as the hole accepter rather than electron accepter because they are closer to the valence band of TiO2. However, the experimental results give an oppositive conclusion.*

R7: Thank you for your valuable comments.

(a) In general, the band gap will be reduced by *interstitial states* when the relative edge positions of valence and conduction bands are not changed after doping. However, the band gap can be improved when the relative edge positions of valence and conduction bands are extended after doping. In particular, the interstitial states are very close to the edge of the valence or conduction band in some doped photocatalysts, such as the present Ba:TiO2. These interstitial states have little effect on reducing the band gap but extend the relative edge

positions of the valence and conduction bands.

(b) It is important to note that the interstitial states composed of Ba 6*s* are predominantly unfilled because most interstitial states are above Fermi level. During photoexcitation, electrons in the filled valence band can readily transfer to neighboring interstitial states though partial electrons can be further excited to conduction band. Therefore, the interstitial states can be negatively charged under continuous light irradiation. Thus, it is not strange that a negative shift was observed in the in-situ XPS spectrum of Ba in Ba:TiO_2.

Q8: *Actually, the position of Fermi level in DOS is set in calculations but not the actual position. This conceptual and fundamental error in this study is unacceptable and misleading ("the barium species create interstitial states near the Fermi level" in the abstract).*

R8: We sincerely appreciate the reviewer's critical and constructive comment on the Fermi level alignment in DOS analysis. We fully clarify this critical point as follows: in our DFT calculations, the energy zero in the DOS plot was indeed set to the calculated Fermi level for better visualization, which is a standard and widely accepted practice in VASP and most first-principles calculations. This alignment does not change the relative position or the physical nature of the electronic states; it only shifts the entire energy axis rigidly for clear presentation. For example, in the DOS plots of Ba:TiO_2, the absolute value of Fermi level is determined to be 4.48 eV. To better visualize the charge distribution, the entire energy axis of DOS plots is negatively shifted by 4.48 eV. As a result, the relative energy value of Fermi level is converted into energy zero.

3. Other issues

Q9: *In the activity measurements, a strong alkaline medium (4.0 M) was used. Previous studies demonstrate that the alkaline conditions could lead to the oxide surface more negatively charged and promote the formation of hydroxyl radical (J. Am. Chem. Soc., 2012, 134, 1974; Nat. Mater., 2014, 13, 1013.). However, this study gives the totally oppositive conclusion (Figures S33 and S34).*

R9: Thank you for your valuable comments. Previous studies indeed demonstrate that strongly alkaline environments can induce more negatively charged oxide surfaces due to enhanced deprotonation of surface hydroxyl groups. As shown in **Fig. S39**, pristine TiO_2 does exhibit a negative shift in *zeta* potential when immersed in alkaline solutions. Meanwhile, our results also reveal a systematic positive shift in *zeta* potential uniquely induced by alkaline earth metal doping, while the sign of the *zeta* potential remains negative. This phenomenon becomes more pronounced with increasing ionic radius of the dopant, demonstrating that it is not a general alkaline effect but a dopant-specific surface modification. O 1*s* of XPS analysis provides direct evidence that alkaline earth metal incorporation significantly increases the density of surface hydroxyl groups (Sci. Bull., 2025, 70, 1452–1461). These additional hydroxyl species act as effective adsorption sites for alkali

cations in alkaline electrolyte (*Nat. Catal.*, 2025, 8, 843–852). Under 0.1 M KOH conditions, the hydroxyl-rich surface formed after alkaline earth metal incorporation can specifically adsorb a larger amount of K^+ ions at the solid-liquid interface. Such enhanced cation adsorption increases the positive charge density in the Stern layer and at the slipping plane, thereby leading to a less negative, *i.e.*, positively shifted *zeta* potential, even though the surface framework remains overall negatively charged (*Angew. Chem. Int. Ed.*, 2024, 63, e202402625). The monotonic dependence of the *zeta* potential shift on the alkaline earth metal ionic radius further supports this interpretation, as larger cation promotes stronger surface hydroxylation and, consequently, more pronounced K^+ adsorption. In short, the observed *zeta* potential evolution originates from a hydroxyl-enriched surface induced by alkaline earth metal doping, which facilitates specific alkali cations adsorption under alkaline conditions, rather than from a reversal of surface charge or a deviation from established alkaline charging behavior.

Q10: *The classic study by Koper et al demonstrates that the first deprotonation of alcohols in alkaline solution is base catalyzed (J. Am. Chem. Soc., 2011, 133, 6914). The authors ignore this situation in all discussion.*

R10: We thank the reviewer for highlighting the seminal work by Koper *et al.* on base-catalyzed alcohol deprotonation. We fully agree that, under alkaline conditions, the hydroxy group of alcohols is firstly deprotonated by base catalysis. When methanol and ethylene glycol are initially subjected to base catalysis, the intermediates formed can be further oxidized through photocatalysis to form byproducts, HCOOH and glycolic acid. However, in our work, the photocatalytic dehydrogenation oxidation of methylene groups in ethylene glycol competes with base catalysis of hydroxy group, resulting in the first formation of •$CHOHCH_2OH$ intermediate instead of •CH_2OCH_2OH. Besides, the photocatalytic dehydroxylation converts methanol into *CH_3 intermediate by cleaving C-O bond. Finally, the C–C coupling reaction between •$CHOHCH_2OH$ and *CH_3 intermediate produces the *$CH_3CHOHCH_2OH$ intermediate which can be successively base-catalyzed and photo-catalytically oxidized into LA. We have revised the manuscript to clarify this division of role, ensuring the mechanistic discussion if fully consistent with established base-catalyzed chemistry and avoids any over-attribution of these spontaneous transformations to photocatalytic effects.

Lines 108–112: *Thus, a *$CH_3CHOHCH_2OH$ intermediate is formed. Under strongly alkaline conditions, the formed *$CH_3CHOHCH_2OH$ intermediate can be successively base-catalyzed and photo-catalytically oxidized into LA. It should be noted that methanol and ethylene glycol would be directly converted into formic acid and glycolic acid when the base catalysis is dominant in the first deprotonation of hydroxy group in alcohols.*

Lines 418–420: *After that, the resulting coupled intermediates are readily transformed into lactic acid*

under alkaline conditions, following successively base-catalyzed and photo-catalytically oxidized chemistry, and vacating the reaction sites.

Q11: *In Figure 5c, the authors attribute the ESR signal to hydroxymethyne radical (α_N = 15.1, and α_H = 21.4) from the EG transformation. However, ·CH_2OH radical (α_N = 15.6, and α_H = 21.5) show the similar signal at the very close position. Therefore, the current assignment is doubtable.*

R11: We thank the reviewer for pointing out the similarity in hyperfine splitting constants between the hydroxymethyne radical and the •CH_2OH radical. We agree that these species cannot be unambiguously distinguished based on ESR parameters alone. However, the radical assignment in our system is supported by reaction chemistry and product selectivity rather than by spectral position only. Under the alkaline photocatalytic conditions employed, methanol does not favor hydrogen abstraction at the *α*-C, making the formation of •CH_2OH radicals unlikely. In contrast, ethylene glycol preferentially undergoes *α*-C–H activation, as widely reported (*PNAS*, 2023, 120, e2312550120; *Angew. Chem. Int. Ed.*, 2024, e202407580). Importantly, reaction pathways involving C–C bond cleavage of ethylene glycol do not lead to lactic acid formation, whereas the observed exclusive formation of C_3 products is fully consistent with a coupling mechanism involving hydroxymethyne intermediates. Therefore, despite the proximity of the ESR signals, the combined evidence from substrate-specific reactivity and product distribution supports the assignment of the detected radical to a hydroxymethyne species derived from ethylene glycol.

Q12: **(a)** *When carefully comparing the in-situ FTIR spectra of Ba-TiO_2, TiO_2 and the neat ATR crystal, it would be found that all labeled absorption peaks for Ba-TiO_2 can be also found in the spectra of TiO_2 and neat ATR crystal, while they present the different assignments. This is self-contradicted.* **(b)** *Moreover, why do the spectra of the neat ATR crystal also show the signals from COO, CO, CH_3?*

R12: We thank the reviewer for this careful and important observation.

(a) We acknowledge that in the original manuscript, several IR bands were assigned in an overly simplified manner, which could indeed create ambiguity when comparing Ba:TiO_2, TiO_2, and the neat ATR crystal. To address this concern, all *in-situ* FTIR spectra have been reprocessed and re-evaluated using stricter assignment criteria. Moreover, **for Ba:TiO_2 and TiO_2**, we note that absorption features in the 1000–1600 cm^{-1} region are commonly associated with carboxylate- and alkoxy-related surface species (*e.g.*, O–C=O, and C–O vibrations) under alkaline alcohol conversion conditions. Therefore, the mere presence of band at similar wavenumbers does not by itself justify specific mechanistic assignments. The critical difference between TiO_2 and Ba:TiO_2 lies in their **spectral evolution and band characteristics** rather than absolute peak position. Specifically, TiO_2 exhibits a relatively sharp feature centered at ~1020 cm^{-1}, whereas Ba:TiO_2 displays a broader and less

intense band around 1010 cm^{-1}. This difference is reproducible across independent experiments and is accompanied by distinct temporal evolution behavior. Importantly, on Ba:TiO_2, a CH_3-related vibrational band progressively emerges and intensifies with reaction time, whereas such evolution is significantly less pronounced on TiO_2. The time-dependent growth of this CH_3 feature, together with the modified band profile in the 1000–1050 cm^{-1} region, is consistent with the formation of coupling-related surface intermediates, as reported in recent studies (*Small*, 2025, 2505626; *Nano Res.*, 2025, 18, 94907765; *J. Phys. Chem. A*, 2025, 129, 5557–5569). We therefore base our mechanistic interpretation primarily on comparative spectral evolution and reproducible differences between catalysts, rather than on isolated peak assignments.

(b) Regarding the signals observed on the neat ATR crystal, monocrystalline silicon was intentionally employed as an alkali-resistant infrared reflection platform to ensure compatibility with the reaction conditions. Under light irradiation, monocrystalline silicon, acting as semiconductor, can also exhibit weak photochemical activity toward alcohols, which explains the appearance of low-intensity COO, C–O, and CH_3 signals. However, the extremely slow evolution of these features confirms that their contribution is negligible compared with that of the catalyst samples, which does not affect the mechanistic interpretation over catalyst samples.

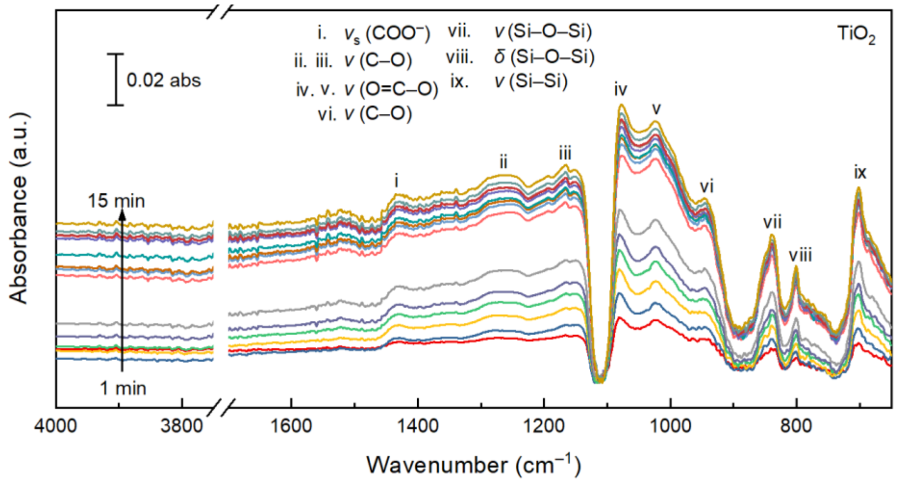

Fig. 5a Time-dependent IR signals for the pristine TiO_2 under our reaction conditions.

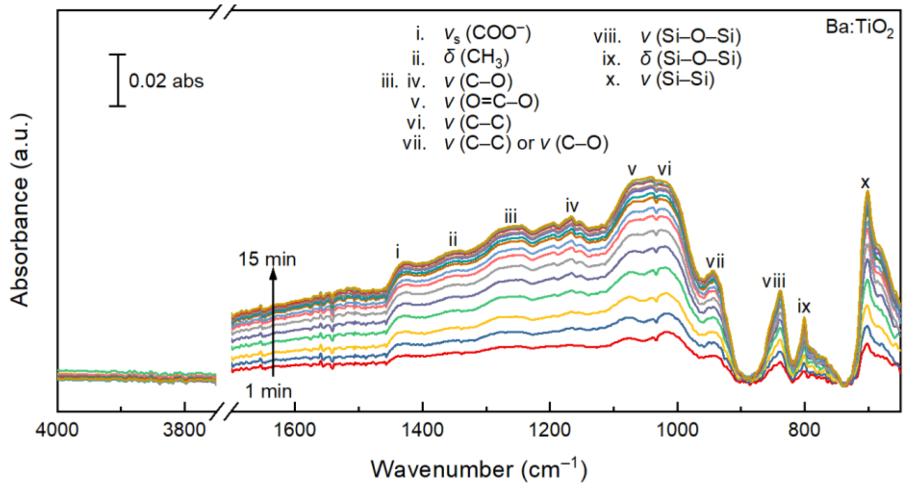

Fig. 5b Time-dependent IR signals for the pristine Ba:TiO_2 under our reaction conditions.

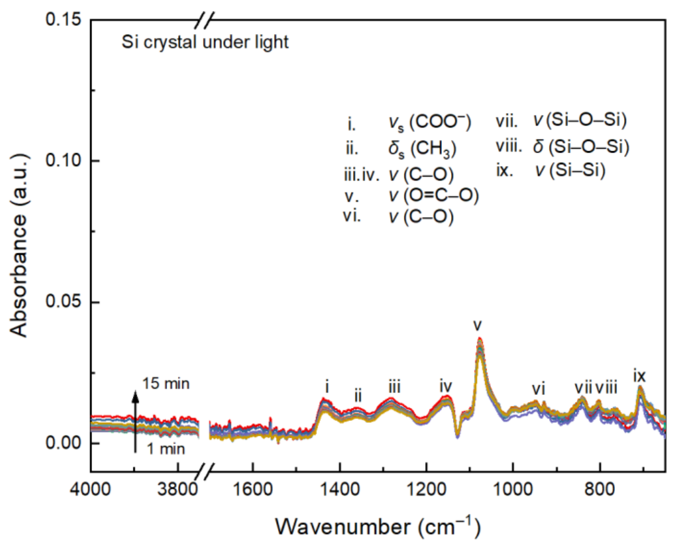

Fig. S37 In-situ attenuated total reflection Fourier transform infrared spectroscopy (ATR-FTIR) spectra over neat ATR crystal, reaction conditions: 5 mg of photocatalysts, 50 vol% MEOH, molar ratio of MeOH to EG is 5:1, concentration of KOH is 4.0 M, T = 25 °C, 1 bar Ar, and Xe lamp irradiation.

Q13: *In the scheme, the coupling between two *CH_3 to form CH_3CH_3 seems more energetically favorable than the coupling between *CH_3 and hydroxymethyne radical, considering the steric effect.*

R13: We thank the reviewer for this insightful mechanistic question. Experimentally, gas chromatography analysis confirms that no ethane is detected under the reaction conditions, excluding $H_3C–CH_3$ coupling as viable pathway. More importantly, our DFT experimental results indicate that *CH_3 species are preferentially stabilized on lattice oxygen sites rather than being freely mobile on the surface, which restricts the movement and coupling of *CH_3 species. Especially, the long-distance movement of *CH_3 species further inhibits the coupling of two *CH_3 species due to the steric effect. In contrast, hydroxymethyne radical generated nearby can readily react with the anchored *CH_3 species at the acid-base interface, enabling selective cross-coupling.

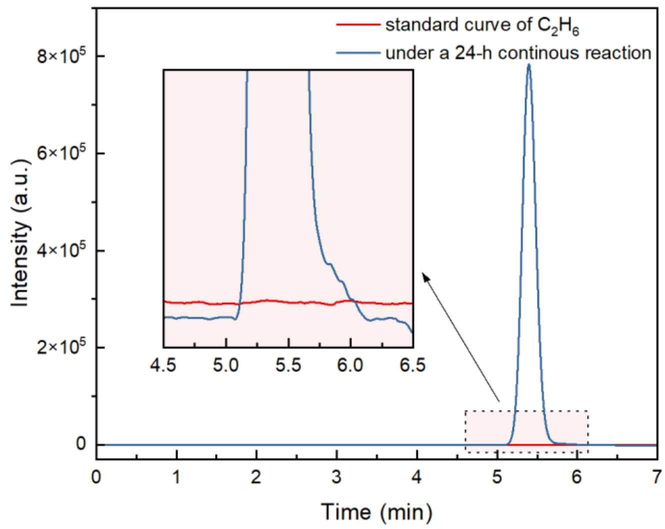

Fig. R3 Gas chromatography analysis results of gas-phase production after a 24-hour reaction using Ba:TiO_2 as photocatalyst.

Q14: *The signal intensity of TA is too weak. High quality data should be provided.*

R14: We thank the reviewer for raising this important point. In response, we repeated transient absorption measurements under optimized conditions to improve the signal quality. The newly acquired data indeed shows enhanced signal-to-noise ratio, which improves spectral definition. We note that the overall spectral evolution and kinetic trends remain consistent with the originally reported data, including the presence of ground-state bleaching features, red-shift behavior at longer delays times, and multi-component relaxation dynamics. Additionally, the absolute lifetime values obtained from global fitting show quantitative differences. We carefully evaluated these discrepancies. The variations in extracted lifetimes are attributed to improved signal quality and fitting robustness in the new dataset, which reduces uncertainty associated with low-intensity regions in the original measurements. Importantly, however, the relative kinetic hierarchy between TiO_2 and Ba:TiO_2 remains unchanged. Therefore, the mechanistic conclusions regarding charge carrier dynamics and Ba-induced modulation remain fully valid. To ensure transparency and provide the highest-quality data to readers, we have now replaced the original TA spectra and corresponding kinetic parameters with the newly acquired dataset in the revised manuscript. (**Figs. 4d–4f**)

Lines 305–319: *Furthermore, the dynamic information of the excited state charge in TiO_2 and M:TiO_2 (Ba:TiO_2) was studied through femtosecond transient absorption spectroscopy (TAS). The samples were photo-excited by 350-nm pump pulses, and the TAS was acquired with a probe pulse. Background subtraction was performed on the data, along with chirp correction. As shown in Figs. 4d and 4e, TiO_2 and Ba:TiO_2 exhibit instantaneous negative signals around the 500 nm upon photoexcitation, which can be primarily attributed to ground-state bleaching (GSB) with possible contributions from simulated emission (SE). For TiO_2, the negative feature gradually narrows and decreases in intensity over time. In contrast, Ba:TiO_2 displays a progressive red shift of the negative band during temporal evolution. To quantitatively analyze the carrier dynamics, global fitting was performed using a sequential kinetic model, yielding the species-associated spectra (SAS) shown in Fig. 4f. Both samples exhibit two dominant kinetic components within the measured time window. For TiO_2, the long-lived component decays within 1.1 ns, indicating relatively fast carrier recombination and limited excited-state persistence. In comparison, Ba:TiO_2 approximately 2.3-fold increase in lifetime (2.6 ns). This prolonged excited-state lifetime suggests that Ba incorporation effectively retards electron-hole recombination dynamics, thereby stabilizing photogenerated charge carriers over longer timescales. Such behavior is consistent with the enhanced photocatalytic performance observed for Ba:TiO_2.*

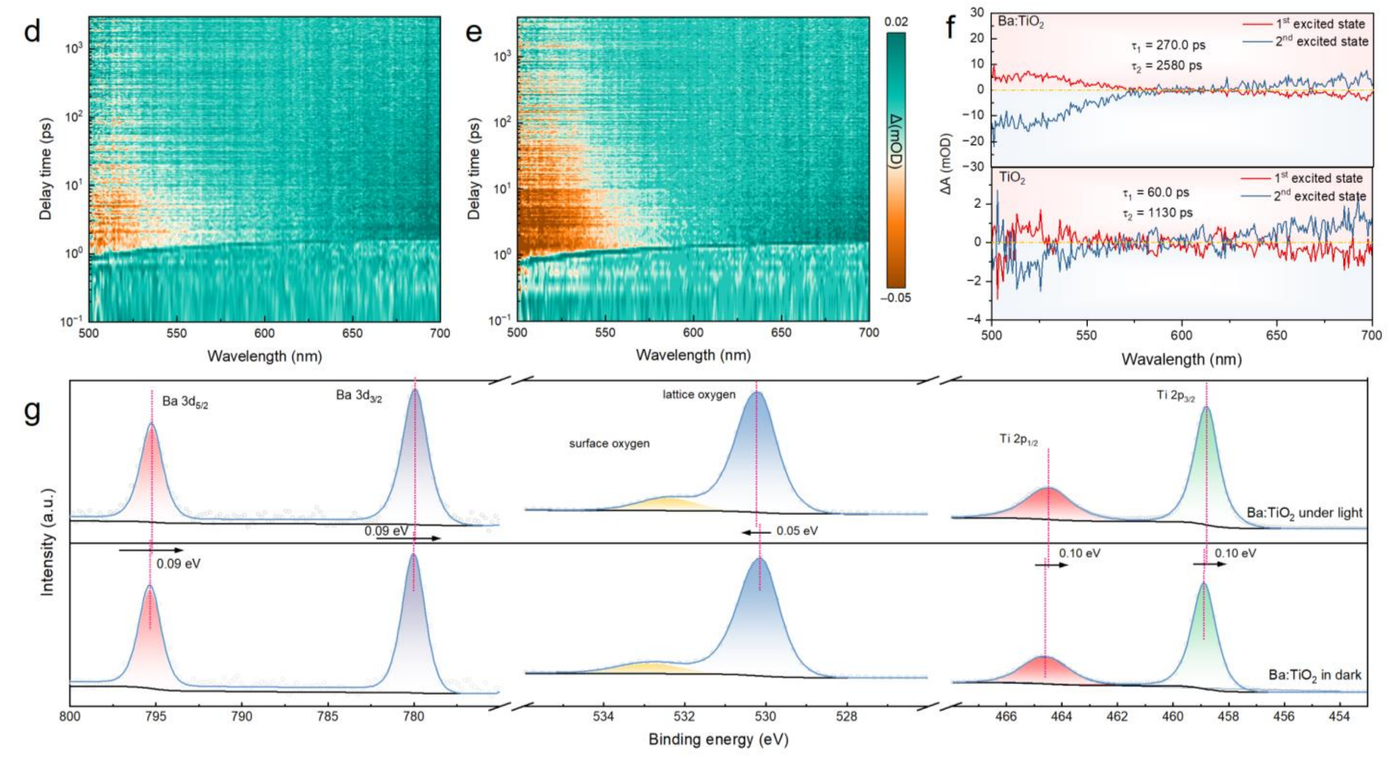

***Figs. 4d–g** TAS spectra of (d) TiO_2 and (e) Ba:TiO_2 under 350-nm excitation, and (f) corresponding SAS spectra. (g) In-situ XPS spectra of Ba:TiO_2 under illumination for 15 min.*

Q15: *In the in-situ XPS spectra, the shift of Ba $3d_{3/2}$ is not consistent with that of Ba $3d_{5/2}$; the shift of Ti $2p_{3/2}$ is not consistent with that of Ti $2p_{1/2}$. Theoretically, the shift should be same.*

R15: We thank the reviewer for this important and technically sound comment. We agree that, in principle, the binding energy shifts of spin-*orbit*-split components (Ba $3d_{5/2}$ and $3d_{3/2}$, Ti $2p_{3/2}$ and $2p_{1/2}$) should be identical within experimental uncertainty. In the revised manuscript, all *in-situ* XPS spectra have been carefully reprocessed. The binding energy scale was recalibrated, and the spectra were refitted using constrained spin-*orbit* splitting and fixed area ratios to ensure internal consistency. After this reanalysis, the Ba $3d_{5/2}$ and $3d_{3/2}$peaks exhibit identical shifts within experimental error, and the same consistency is observed for the Ti $2p$ doublets. The apparent discrepancy in the original figure therefore arose from fitting and calibration artifacts rather than from a physical inconsistency. The corrected spectra and fitting parameters are now provided in the revised figure (**Fig. 4g**).

Q16: *The implantation of Ba leads to the lattice expansion, meaning that length of the Ti–O bond is increased. Thus, the strength of the Ti–O bond should be weakened. However, the Raman spectra confirm the enhanced Ti–O bond. An explanation should be provided.*

R16: We thank the reviewer for this important comment. The apparent discrepancy arises from the fact that XRD and Raman spectroscopy probe different crystallographic directions and vibrational modes of the Ti–O

framework. The XRD peak shift corresponds to the (1 0 1) reflection, which reflects changes in the interplanar spacing along the specific crystallographic direction normal to the (1 0 1) plane. This shift therefore represents variation in the averaged lattice parameter along that direction. In contrast, the Raman band at ~144 cm^{-1} corresponds to the E_g mode of anatase TiO_2, which is associated with symmetric Ti–O stretching vibrations within the TiO_6 octahedra. The atomic displacement pattern of this E_g mode does not coincide with the lattice expansion direction associated with the (1 0 1) reflection. Therefore, the Raman blue shift reflects a modification of the vibrational force constant of specific Ti–O bonds involved in this mode, rather than a direct measurement of the interplanar spacing detected by XRD. In short, **lattice expansion along the (1 0 1) direction does not necessarily imply weakening of the Ti–O bonds participating in the E_g vibrational mode**. The two observations probe different structural components of the Ti–O network and are therefore not contradictory. This phenomenon has also been reported in previous literatures (*Angew. Chem. Int. Ed.*, 2025, 64, e202518232; *ACS Catal.*, 2025, 15, 19135–19147).

Q17: *The Raman signal above 1000 cm^{-1} should be presented to confirm the presence of residual carbon in the catalysts or not.*

R17: We thank the reviewer for this constructive suggestion. In response, we have examined the Raman spectra of Ba:TiO_2 in the region above 1000 cm^{-1} to assess the possible presence of residual carbon species. No characteristic D or G bands associated with carbonaceous residues are observed, confirming that the synthesized catalysts are free of residual carbon.

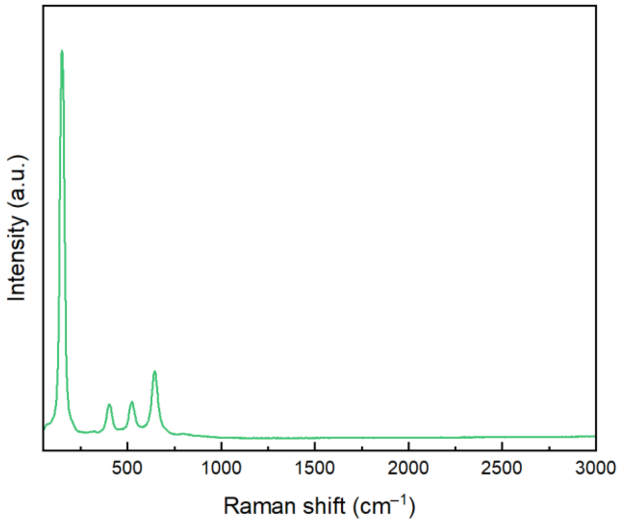

***Fig. S9** Raman spectrum of Ba:TiO_2, laser wavelength: 532 nm.*

Q18: *Captions of Figure 1b and 1c are muddled up.*

R18: Thank you for pointing this out. We have corrected the captions of **Fig. 1**.

Lines 142–148: Fig. 1 Investigation of thermochemical reaction energetics. (a) *Charge density difference*

mapping between $^*CHOHCH_2OH$ intermediate and catalyst surface on TiO_2 (left) and Ba:TiO_2 (right), the skyblue and yellow isosurfaces stand for the negative and positive charges, respectively; the isosurface of charge density is set to 0.002 e $Å^{-3}$. ***(b)*** *Free energy profile of lactic acid (LA) production on TiO_2 and Ba:TiO_2 at pH = 7 and U = 0 V vs. SHE.* ***(c)*** *Projected density of states (PDOS) of C and O 2p states of $^*CHOHCH_2OH$ intermediates, the dashed line stands for the Fermi level.* ***(d)*** *Total density of states (TDOS) and PDOS of Mg, Ca, Sr, and Ba-modified bulk TiO_2.*

Q19: *A careful language review is recommended. For example, "owing to the introduction of alkaline earth metals, the strength of Ti–O bonds is enhanced, leading to the absorption edges of M:TiO_2 (ca. 430 nm), which results in a slight blue shift compared with TiO_2 (ca. 450 nm)."*

R19: Thank you for pointing this out. The specific example mentioned has been corrected accordingly. In addition, the entire manuscript has been carefully edited to improve language precision and readability.

Reviewer #5 (Remarks to the Author):

I have accessed the revised manuscript and can conclude that a lot of work has been put in addressing the reviewers' comments. Specifically, to the comments of Referee 3, most of the technical comments have been clarified (data quality, additional measurements, QY calculations, clarification of the PL datasets and optimization of the statements made in the main manuscript). Nevertheless, several comments have been addressed less satisfactorily. As such:

Q1: *Ref 3, Q12: I would still agree with the referee's critisims: experimental data presented in the MS (also in its revised version) confirm Ba incorporation by detecting Ba-O bonds, but it is not an indicator for Ba single atoms. Ba-based oxo-clusters on TiO_2 would show a similar bonding situation and thus cannot be excluded. The authors conduct EXAFS and suggest "Especially, the fitting results of EXAFS further confirms the atomically dispersed nature of Ba coordinated with six O atoms, consistent with the above theoretical model", however it is unclear which model is meant. The one shown in Figure 1a features only surface Ba atoms which are clearly not 6-fold coordinated.*

R1: Thank you for your valuable comments. Directly identifying the surface Ba and bulk Ba atoms is a challenge in the characterization. However, the STEM firstly demonstrates the existence of atomically Ba species without observable *Ba-based oxo-clusters* (**Fig. 2f**). Additionally, the X-ray photoelectron spectroscopy (XPS) depth profiling and the focused ion beam-scanning electron microscopy (FIB-SEM) cross-sectional elemental distribution analysis directly revealed the uniform distribution of Ba atoms in the whole Ba:TiO_2 including surface and bulk sites (**Figs. S10** and **S11**). Moreover, the fitting results of EXAFS further confirms the atomically dispersed nature of Ba coordinated with 5.88 O atoms. It should be noted that the Ba species in bulk TiO_2 tend to replace Ti atom, which is coordinated with six O atoms (**Fig. R5a**). The surface Ba species are coordinated with five O atoms (**Fig. R5b**). Therefore, the average coordination number of Ba species in whole TiO_2 is located between 5 and 6, like the above experimental value (5.88). To improve understanding, we have revised the manuscript.

Lines 98–99: *Additionally, the thermodynamically stable anatase (1 0 1) facets were considered in the simulation. The alkaline-earth metals tend to coordinate with five O atoms in anatase (1 0 1) facets.*

Lines 135–136: *According to the structural feature of TiO_2 bulk, the Ba species in bulk TiO_2 tend to replace Ti atom, which is coordinated with six O atoms.*

Lines 179–182: *Especially, the fitting results of EXAFS further confirms the atomically dispersed nature of Ba coordinated with 5.88 O atoms. According to above models, the surface and bulk Ba species are coordinated with five and six O atoms. The theoretical average coordination number of Ba in whole TiO_2 is*

between five and six, consistent with the fitting result of EXAFS.

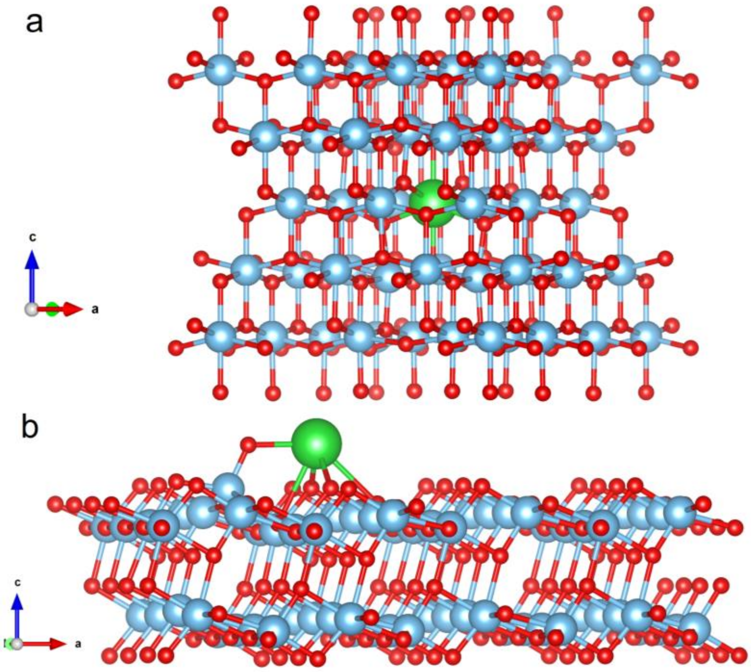

Fig. R5 *Optimized structures of (a) bulk and (b) surface Ba species upon TiO_2.*

Q2: *Ref 3, Q11: on a similar note, not enough information is given in the DFT section on the actual atomistic models used in the calculations: what are the explored positions of Ba, how have its optimized locations been determined? DFT models presented at the beginning of the MS seem to consider surface Ba (surface decoration and its effect on catalysis), but experiments shown later also reveal that Ba incorporates inside the material as a dopant. This seems not to be considered in the DFT models. This creates dissonance: when modelling surface reactions, Ba inside the lattice is ignored; when modelling the structure, Ba on the surface is ignored. This requires more clarification and the reveal of the details of the DFT models and their progressive optimisation.*

R2: Thank you for your valuable comments. According to R1 above, we have classified the positions of Ba species in TiO_2 surface and bulk. The structural feature of TiO_2 enables the coordination numbers of Ba species to be five in surface and six in bulk, which is obtained from the geometry optimization of DFT model. In the bulk region, we mainly consider the doping effect of Ba on the band structure and optical properties since the bulk TiO_2 dominates the photoexcitation. In contrast, the surface Ba species directly determines the surface catalysis. Therefore, the bulk Ba species produce little effect on surface catalysis compared to surface Ba species. Similarly, the surface Ba species produce little effect on photoexcitation compared to bulk Ba species.

Q3: *Scheme 1: right bottom: "sites", "cleaving"*

R3: Thank you for pointing this out. We have revised the **Scheme 1** according to the reviewer's suggestion.

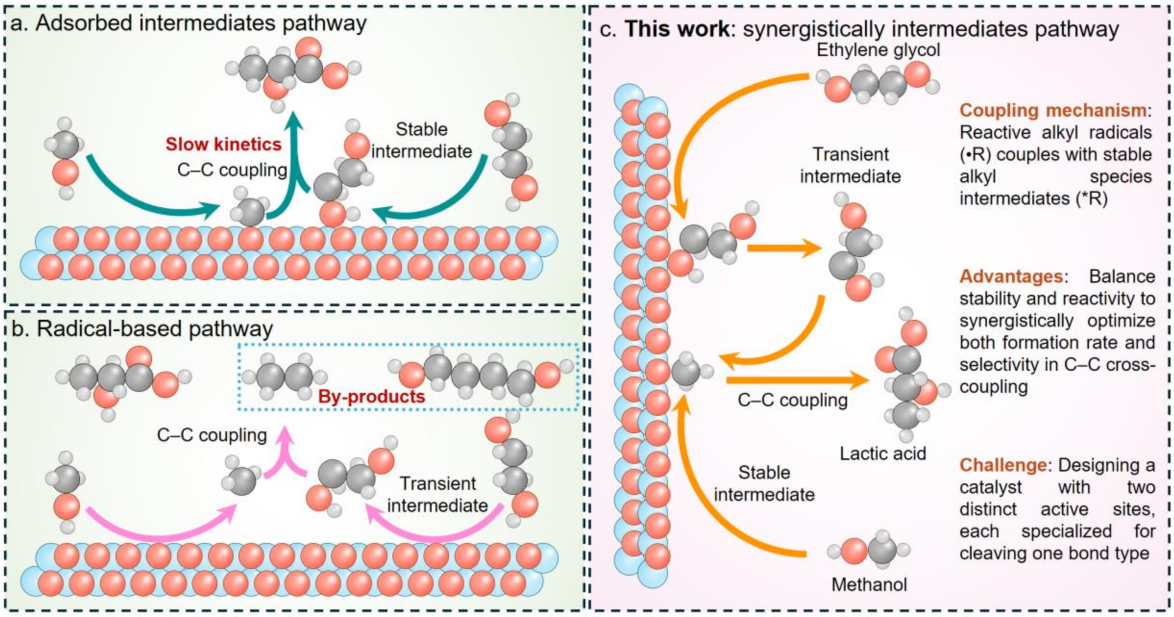

Scheme 1. *Illustration for the coupling of stable and transient intermediates for the synthesis of C_3 compounds from PET-derived ethylene glycol*

Q4: *Another comment on the choice of the DFT model: how strongly does the chosen anatase surface correspond to or represent the actual MIL-125-derived TiO_2 NPs? The surface shown in the simulation is flat. The derived NPs (Figure 2e) are not faceted. Ba location is thus not justified.*

R4: Thank you for your valuable comments. Firstly, the anatase (0 0 1) facet has been considered the most stable and representative surface for TiO_2 materials including nanoparticles, which has been widely used in many reported works (*Nat. Chem.*, 2025, 17, 890–896; *Angew. Chem. Int. Ed.*, 2025, 64, e202505630; *Nat. Commun.*, 2023, 14, 4431). Although some edge regions in TiO_2 nanoparticles may potentially act as catalytic sites, their instability and limited quantity cannot support effective catalysis. Additionally, directly identifying the three-dimensional atom configuration of those edge regions in TiO_2 NPs by experimental measurements is a challenge in this field. Therefore, we used the typical anatase (0 0 1) facet to simulate the surface of TiO_2 NPs.

Q5: *What are "dual" Ba–O bonds? Double?*

R5: Thank you for your valuable comments. The dual Ba–O bonds are two chemical Ba–O bonds formed between one Ba site and two O atoms of $*C_2H_5O_2$ intermediate from ethylene glycol dehydrogenation. To improve understanding, all "dual" words have been revised into "double".

Q6: *If the authors suggest that Ba replaces Ti in the lattice, this needs to be stated clearly throughout the MS text (refer to Ref 3, Q2).*

R6: Thank you for your valuable comments. Considering the atomic radius of Ba and geometry structure of TiO_2 crystal, Ba tends to replace Ti site in the lattice of TiO_2. The interstitial Ba or replacing lattice O site can be excluded. Therefore, Ba replaces Ti in the lattice. We have revised the section of Theoretical calculation

Lines 533–537: *The surface of anatase TiO_2 surface was described by its typical and stable (1 0 1) facet which was simulated by one 2 × 3 (1 0 1) supercells consisting of six O-Ti-O layers. Considering the atomic radius of Ba and geometry structure of TiO_2 crystal, Ba tends to replace Ti site in the lattice of TiO_2. Based on the atomic configuration of anatase TiO_2 crystal, the coordination numbers of Ba in TiO_2 bulk and surface were determined to be six and five, respectively.*

Q7: I don't see the indicated absorption edges from Figure S22. For example, TiO_2 does not absorb "from 450 nm". This would also go completely against the known anatase band gap values reported in the literature.

R7: Thank you for pointing this out. We have revised the sentence with "*The incorporation of alkaline earth metals enhances the Ti–O bond strength. As a consequence, the absorption edge of M:TiO_2 shifts to approximately 380 nm, exhibiting a slight blue shift compared to pristine TiO_2 (ca. 410 nm)*".

Reviewer #4

The reviewer appreciates the authors' response to the comments. They did some supplementary experiments to address my concerns, but some issues still remain.

Q1: *The authors attributed the inconsistence in activity data between normal conditions and AQY test conditions to the employment of different light source. To support their claim, they should provide the light intensity profiles of the broadband light source and the monochromatic light source.*

R1: We thank the reviewer for this helpful comment. Following the reviewer's suggestion, we measured the spectral profiles of the monochromatic light source used for the *AQY* tests and the broadband Xe lamp used for the photocatalytic performance measurements. As shown in **Fig. R1**, the monochromatic irradiation used in the *AQY* test exhibits a higher integrated spectral intensity than the broadband Xe lamp in the main absorption region of TiO_2 (300–400 nm), which reasonably explains the higher lactic acid production rate derived from the *AQY* measurement.

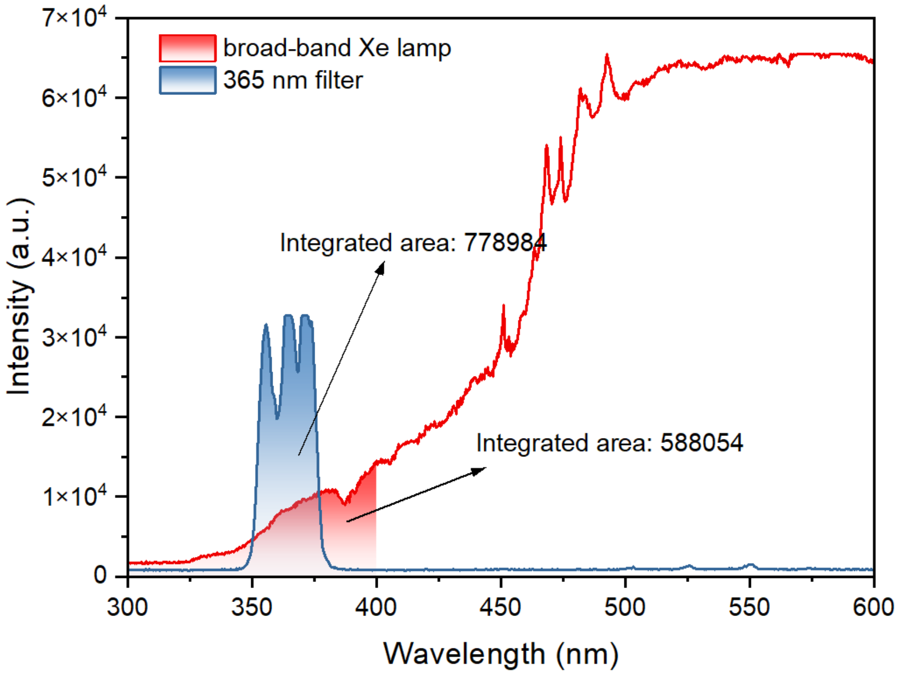

Fig. R1 *Adsorption spectra of Xe lamp (360 mW cm^{-2}) and monochromatic light source (365 nm band-pass filter with another Xe lamp, 470 mW cm^{-2}), measurement via fiber optic spectrometer (FX2000, Shanghai Fuxiang)*

Q2: *The authors attributed the non-zero AQY observed at 420 nm to the excitation of the residual high-energy light. However, the similar situation also happened for the AQY tests at other wavelengths. This means all reported AQY values are overestimated.*

R2: Thank you for this valuable comment. We agree that the spectral bandwidth of practical light sources may introduce a minor contribution from higher-energy photons in wavelength-dependent apparent quantum yield

(*AQY*) measurements. However, this issue only arises when the photon wavelength is located at the absorbance edge of the photocatalyst. For instance, anatase TiO_2 has an absorbance edge at 387 nm; theoretically, it cannot be directly photoexcited by 400 nm photons. Nevertheless, a portion of the photons emitted through a 400 nm filter extends to wavelengths around 387 nm (**Fig. R2**). This phenomenon is not unexpected, as filters allow a distribution of photons centered at 400 nm rather than strictly monochromatic 400 nm light—this is also the case for some LED lamps (**Fig. R3**). Consequently, anatase TiO_2 can be photoexcited by photons from a 400 nm filter, although its activity remains very low due to the small proportion of photons with wavelengths shorter than 400 nm (*i.e.*, higher energy). In contrast, when the wavelength of the filter or LED lamp is far from the absorbance edge of the photocatalyst, this effect exerts negligible influence on *AQY*. For example, when the filter or LED lamp wavelength is 450 nm, all emitted photons have wavelengths longer than 387 nm (**Fig. R4**), resulting in no observable activity in *AQY* measurements. On the other hand, when the wavelength is 365 nm, all photons have wavelengths shorter than 387 nm (**Fig. R5**). Although some photons with wavelengths shorter than 365 nm may slightly enhance the *AQY* at 365 nm, other photons with wavelengths longer than 365 nm (but still shorter than 387 nm) will slightly reduce it. As a result, the wavelength tailing effect leads to minimal changes in the *AQY* of TiO_2 at 365 nm.

In summary, *AQY* measurements should be conducted under light irradiation with wavelengths shorter than the absorbance edge of the photocatalyst. For this reason, we measured the *AQY* at 365 nm. We also agree that developing more rigorous methodologies for evaluating the energy utilization efficiency in photocatalysis would be highly valuable for the field.

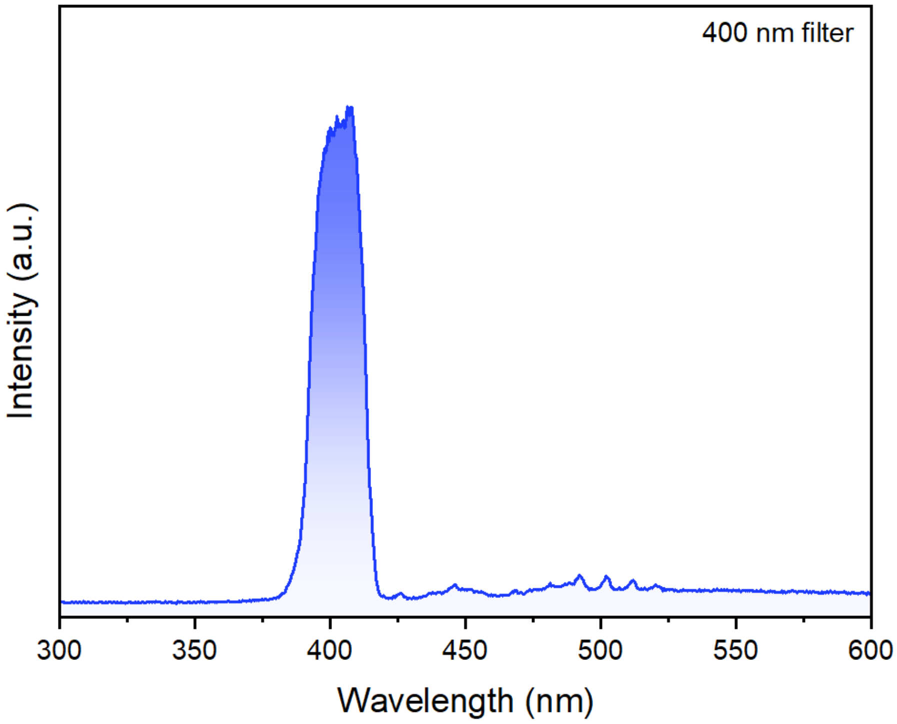

***Fig. R2** Adsorption spectra of monochromatic light source through using a 400 nm band-pass filter with Xe lamp (PLS-SXE300D, Beijing Perfectlight)*

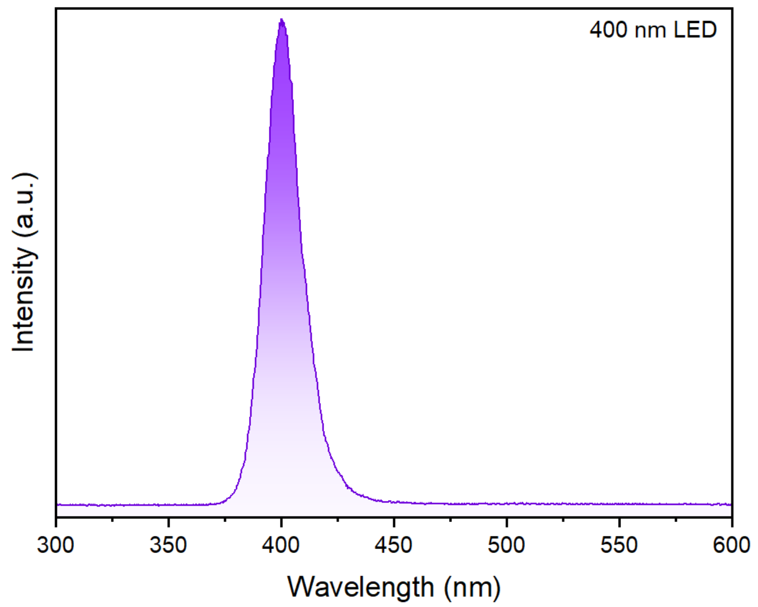

Fig. R3 *Adsorption spectra of a LED lamp (400 nm, MC-LED100H, Beijing Merry Change, China)*

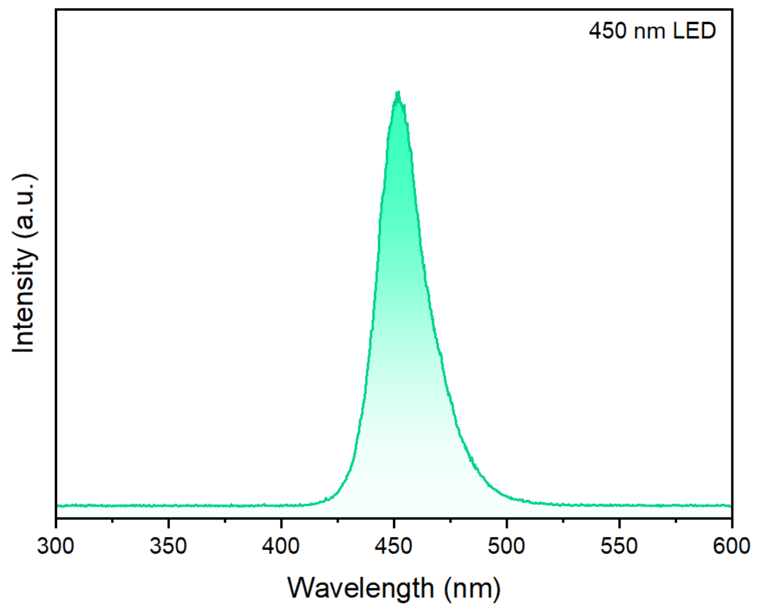

Fig. R4 *Adsorption spectra of a LED lamp (450 nm, MC-LED100H, Beijing Merry Change, China)*

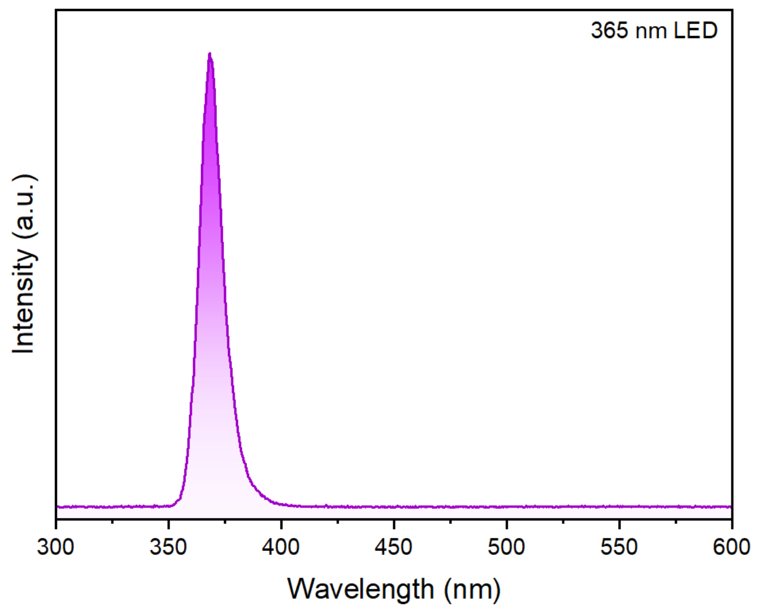

Fig. R5 *Adsorption spectra of a LED lamp (365 nm, PLS-LED 100C, Beijing PerfectLight)*

Q3: *The standard curves of different products were updated, but the activity data were not corrected.*

R3: Thank you for this helpful comment. The activity data has already been corrected according to the updated calibration curves in our previous revision. The revised values are now consistently reflected in the corresponding figures and tables in the manuscript. We have carefully rechecked the dataset to ensure that all reported catalytic activities are calculated using the updated standard curves.

Q4: *From the standard HPLC spectra of different products shown in Fig. S17, the peak positions for formic acid and glycolic acid are very close, which would interfere their quantitative analysis.*

R4: Thank you for this helpful comment. To evaluate the potential overlap between the glycolic acid and formic acid peaks, we calculated the chromatographic resolution (R_s) between two peaks according to the IUPAC definition:

$$R_s = \frac{2 \times (t_2 - t_1)}{W_1 + W_2}$$

where t represents the retention time and W denotes the peak width.

The calculated R_s value between the glycolic acid and formic acid peaks in **Fig. S23d** is 1.17, indicating the sufficient separation for quantitative analysis according to the established HPLC methodology (Lloyd R. Snyder *et. al.*, *Practical HPLC Method Development*). In principle, a spectral resolution above ~1.0 is generally sufficient for quantitative analysis. In particular, the peaks of formic acid and glycolic acid exhibit minimal overlap. Accordingly, the separation achieved between formic acid and glycolic acid under the actual analytical conditions is adequate to ensure reliable quantification.

Q5: *When checking the relative peak intensities for lactic acid, formic acid, and glycolic acid in the HPLC spectra of Ba:TiO_2 in Fig. S25, the selectivity of lactic acid seem to be not as high as the reported.*

R5: Thank you for this helpful comment. The discrepancy in the selectivity of lactic acid mainly arises from the background signal of glycolic acid before photocatalytic reaction because the reaction solution was prepared under aerobic conditions and strongly alkaline conditions, leading to the partial oxidation of ethylene glycol by oxygen into a small amount of glycolic acid. As shown in **Fig. R6**, the background signal of glycolic acid is present before light irradiation. However, when the prepared reaction solution was stored under an Ar atmosphere, no obvious increase in glycolic acid was observed. Upon light irradiation, both glycolic acid and lactic increased significantly, indicating that these products were generated under photocatalytic conditions. Therefore, in the quantitative HPLC analysis, the background signal of glycolic acid present before the photocatalytic reaction was subtracted to avoid overestimating its contribution to the photocatalytic products. We have now clarified this point in the revised *Supplementary Information.*

"A small background signal of glycolic acid may be present before photocatalysis due to partial oxidation of ethylene glycol under aerobic alkaline conditions; this contribution was subtracted during quantitative analysis".

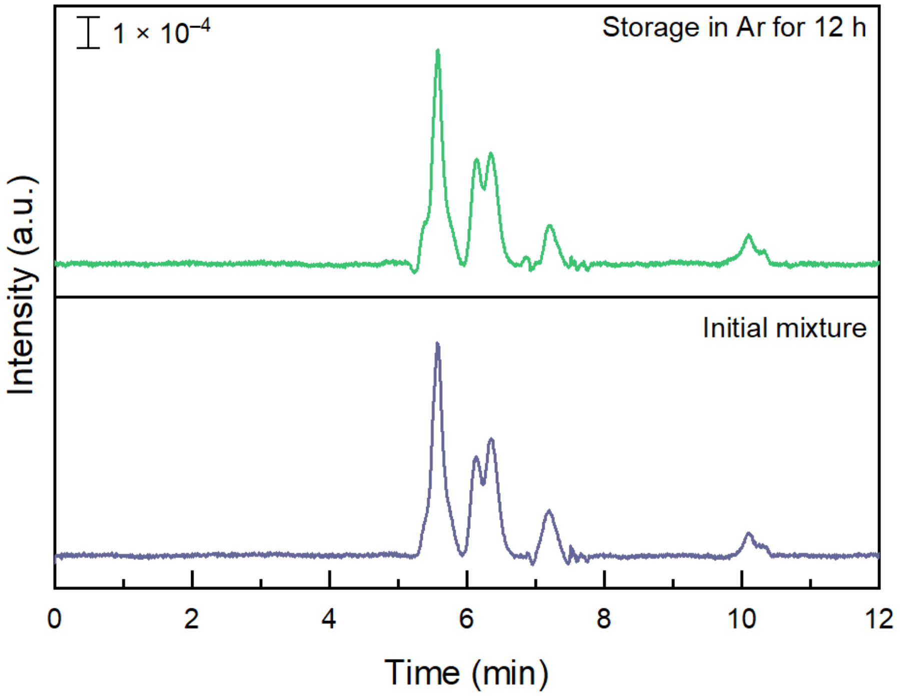

Fig. R6 *HPLC spectra of initial reaction mixture and the mixture storage in Ar for 12 hours*

Q6: *In the DOS plots of Ba:TiO_2, the interstitial states from Ba are close to the top of valence band, it means they prefer to accept holes from the valence band or the occurrence of the excitation from the interstitial states to the conduction band. This is against to the authors' core claim.*

R6: Thank you for your valuable comment. First, *in-situ* XPS measurements directly revealed a negative binding energy shift in the Ba 3*d* XPS spectrum, providing experimental evidence for electron accumulation at the Ba sites under photoexcitation. Second, from a theoretical perspective, Ba species are inherently positively charged and thus tend not to lose additional electrons. This is also demonstrated by the calculated PDOS in which most Ba 6*s* states are located above the Fermi level, suggesting that those Ba 6*s* states are mainly unfilled. Therefore, those Ba 6*s* states cannot effectively capture holes from the valence band though they are close to the top of valence band. Instead, those Ba species can capture the electrons from the top of valence band during photoexcitation and then only partial electrons can be further photoexcited into the conduction band of TiO_2. Thus, the left electrons in Ba species lead to the negative shift in its in-situ XPS spectrum. In this photoexcitation process, those Ba 6*s* states act as the charge bridge between valence and conduction band.

Q7: *The claim that the barium species create interstitial states near the Fermi level is not correct, because the*

Fermi level is set to energy zero. The relative positions of CB, VB and interstitial states to the Fermi level are invalid in the present DFT calculations. For example, the current calculations show the Fermi level is close to VB for all materials, but the actual situation is that the Fermi level is close to CB due to their n-type feature.

R7: Thank you for your valuable comment. Firstly, in the calculation of PDOS in VASP, all energy values, including PDOS and Fermi level, are subtracted by the value of Fermi level in the corresponding models. Therefore, the value of Fermi level is zero in the previous PDOS plots. In other words, the energy value of PDOS plots is calculated relative to the value of Fermi level. To accurately describe the energy value of PDOS plots, the caption of x-axis in the PDOS plots should be changed from "Energy (eV)" to "$E - E_f$ (eV)" in which E and E_f stands for the absolute values of PDOS and Fermi level relative to the vacuum energy level, respectively. It should be noted that the relative positions of the conduction band (CB), valence band (VB), and interstitial states with respect to the Fermi level can be compared within the same model, but not across different models.

Secondly, another issue should be noted that n-type or p-type semiconductors are the doped semiconductors in which the Fermi level is in the bottom of conduction band or the top of valence band, respectively. The semiconductor without doping or defect is the intrinsic semiconductor in which the Fermi level is commonly located in the center region of band gap. Commonly, TiO_2 is considered as the n-type semiconductor in the experiments because a few oxygen vacancies unavoidably exist in TiO_2 crystal since the perfect TiO_2 crystal cannot be achieved by the experimental method. However, in our model, we mainly consider the electronic state distribution of Ba. Owing to the lower number of valence electrons in Ba relative to Ti (like boron-doped silicon), the Ba 6s states are generally located above the valence band.

Q8: *The authors attributed the discrepancy between XRD and Raman to the fact that XRD and Raman spectroscopy probe different crystallographic directions and vibrational modes of the Ti–O framework. However, in fact, all XRD peaks of Ba:TiO_2 are shifted to larger angles relative the corresponding peaks of TiO_2.*

R8: We thank the reviewer for this insightful comment. We agree that the systematic shift of all XRD peaks towards lower angles indicates an overall lattice expansion after Ba incorporation into the TiO_2 lattice. This observation reflects an increase in the average lattice parameters of the crystal structure due to the longer Ba-O bond than pristine Ti–O bond. However, it should be noted that the existence of Ba-O bond would lead to the local extrusion stress in the neighboring Ti–O bond according to the DFT simulation, resulting in the shorter Ti–O bond (1.84 Å) compared to Ti–O bond (1.94–1.96 Å) in other regions (**Fig. S16**). Since Raman spectroscopy probes the vibrational properties of specific bonds (Ti–O bond) rather than the average lattice spacing, the shortening of the Ti–O bond can lead to a blue shift of the Ti–O vibrational mode even though

the average lattice (Ti–O and Ba–O) expands. Therefore, XRD and Raman spectroscopy show the moving toward low angle and high wavelength (blue shift), respectively. Similar behavior has also been reported in other doped TiO_2 systems in which lattice expansion detected by XRD is accompanied by a Raman blue shift due to local distortion and bond-strength redistribution rather than uniform bond weakening (*Angew. Chem. Int. Ed.*, 2025, 64, e202501837; *Adv. Mater.*, 2024, 36, 2312343; *ACS Nano*, 2025, 19, 31915–31928; *Energy Envrion. Sci.*, 2025,18, 9478–9489).

Lines 185–187*: The existence of Ba–O bond would lead to the local extrusion stress in the neighboring Ti–O bond, resulting in local distortion and bond-strength redistribution.*

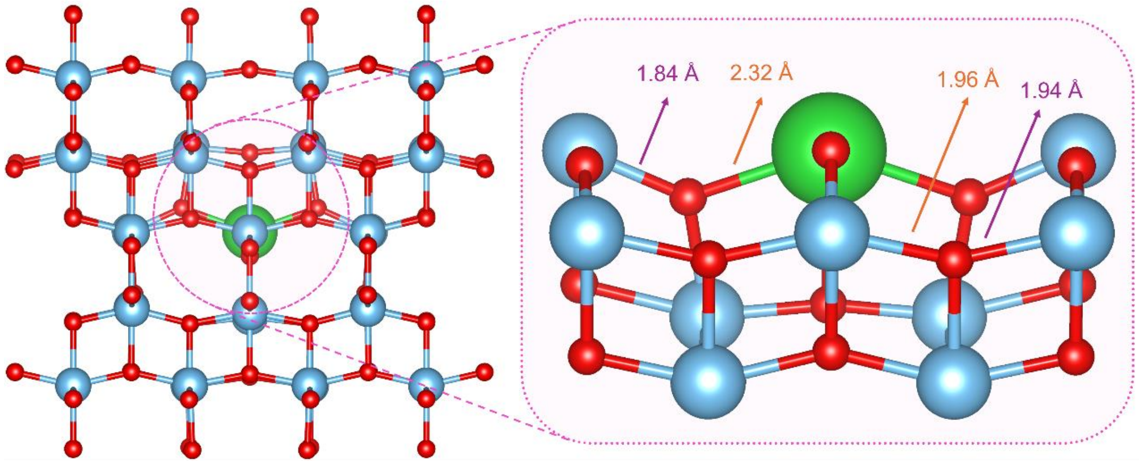

Fig. S16 *Optimized structures of bulk Ba:TiO_2.*

Reviewer #5

Q1: *Both my comments 1 and 2 (based on the original Ref 3 comments) have not been adequately addressed.* ***(a)*** *The presence of Ba-oxo clusters cannot be ruled out.* ***(b)*** *The details on the fundamental structural models have not been provided.*

R1: We thank the reviewer for this insightful comment.

(a) First, EXAFS analysis confirms the exclusive presence of Ba–O bonds in Ba–TiO_2 (**Fig. 2h**). If Ba species were predominantly present as Ba-oxo clusters, a strong Ba–Ba coordination signal would be expected in the second shell. However, such a feature is absent in the experimental EXAFS spectra. Second, atomically dispersed Ba species are clearly observed in numerous STEM images (**Fig. R7**), ruling out the possibility that the atomic dispersion of Ba arises from random or occasional occurrence.

(b) In the revised section of theoretical calculation, we added more detailed description on models of surface and bulk Ba.

Lines 528–541: *Considering the atomic radius of Ba and the structural characteristics of the TiO_2 crystal, a bulk Ba-doped TiO_2 model was constructed by substituting one Ti atom with a Ba atom in a 2 × 2 × 1 anatase TiO_2 supercell (**Fig. S41a**), yielding six-coordinated Ba species. The anatase TiO_2 surface was represented by its typical and stable (101) facet, modeled using a 2 × 3 (101) supercell composed of six O–Ti–O layers. Based on the local structure of the TiO_2 (101) surface, Ba is thermodynamically favored to occupy a five-oxygen-coordinated site (**Fig. S41b**), forming five-coordinated Ba species. Consequently, the average coordination number of Ba in the overall TiO_2 sample is between 5 and 6, consistent with the experimental EXAFS fitting result of 5.88. Given the dominant role of surface Ba species in surface catalytic reactions, bulk Ba species were not placed in the deep subsurface layers, and only surface Ba sites were employed in the calculations of the surface reaction pathway. Besides, given that bulk photoexcitation in photocatalysts is predominantly governed by the bulk structure rather than the surface structure, the influence of surface Ba species on bulk photoexcitation was neglected in the projected density of states (PDOS) calculations. Accordingly, surface Ba species were employed in modeling surface catalytic reactions, while bulk Ba species were used to evaluate bulk photoexcitation properties.*

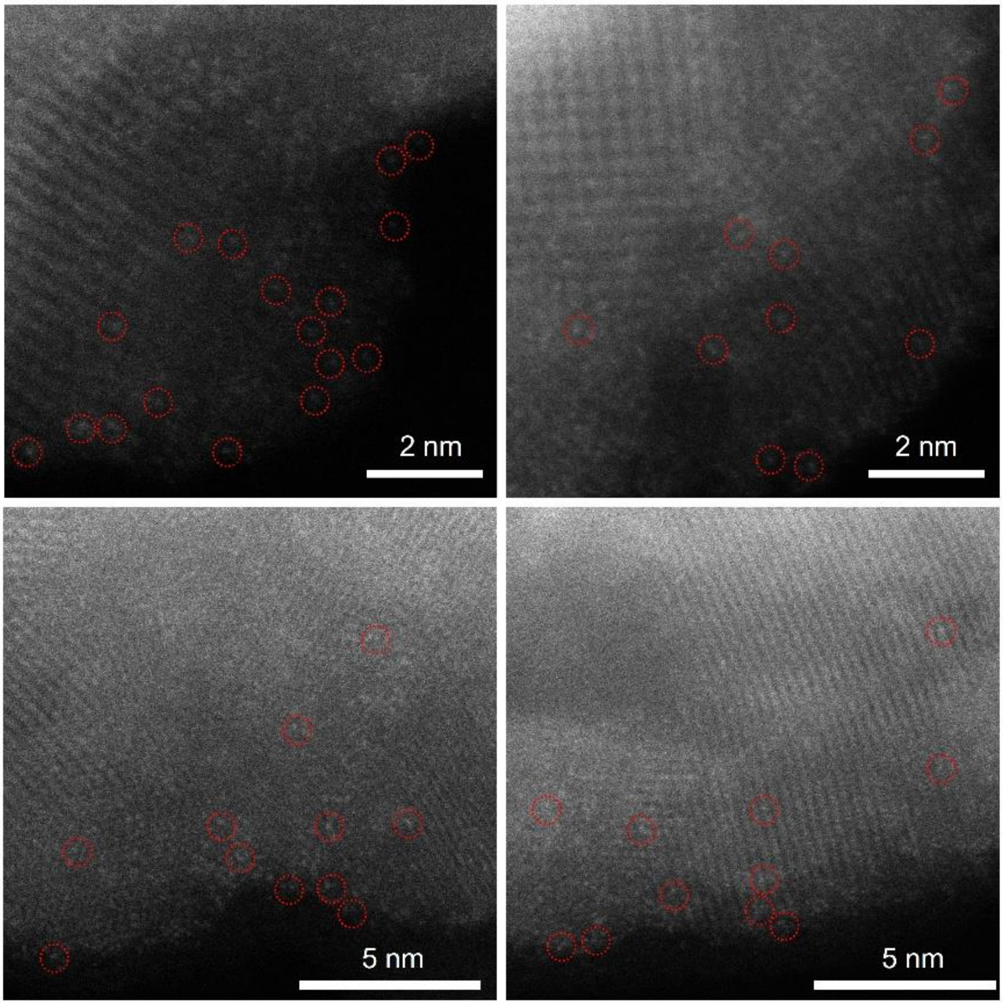

Fig. R7 *The aberration-corrected scanning transmission electron microscopy of Ba:TiO_2.*

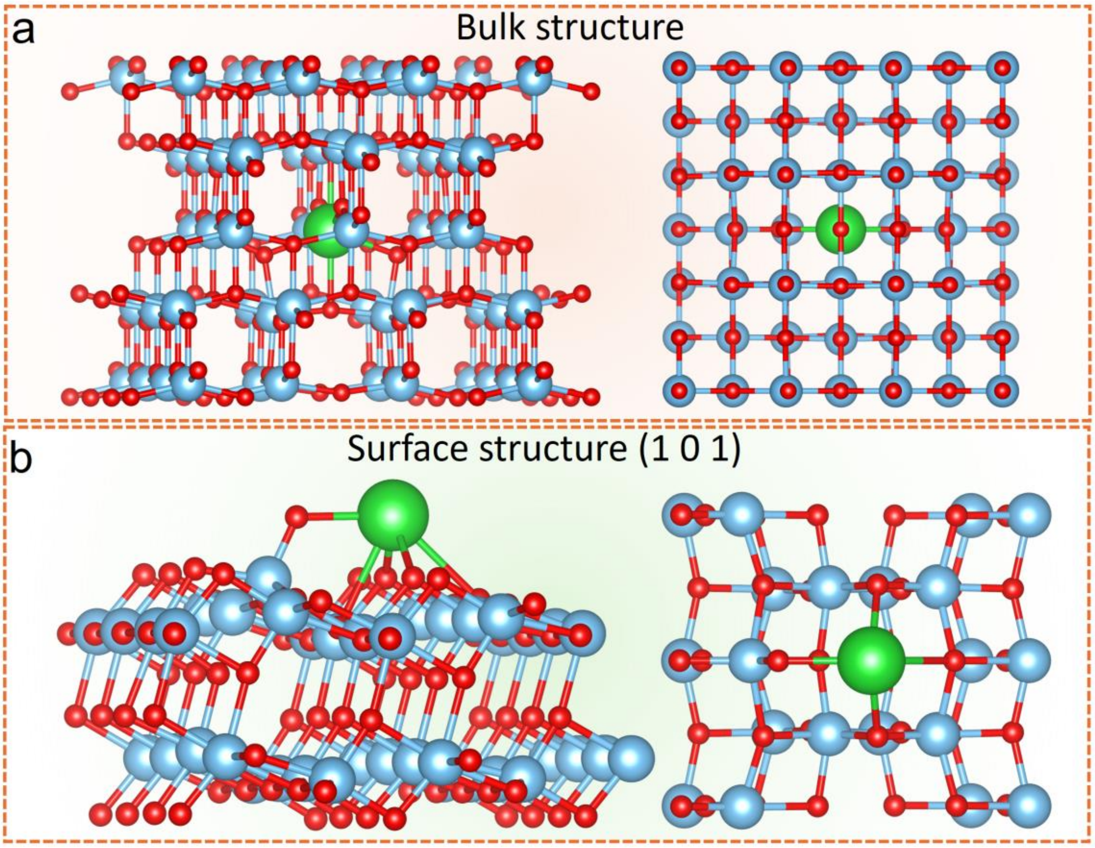

Fig. S41 *Optimized structures of (a) bulk and (b) surface incorporation of Ba upon TiO_2.*

Q2: *Ref 3, Q11: on a similar note, not enough information is given in the DFT section on the actual atomistic models used in the calculations: what are the explored positions of Ba, how have its optimized locations been determined? DFT models presented at the beginning of the MS seem to consider surface Ba (surface decoration and its effect on catalysis), but experiments shown later also reveal that Ba incorporates inside the material as a dopant. This seems not to be considered in the DFT models. This creates dissonance: when modelling surface reactions, Ba inside the lattice is ignored; when modelling the structure, Ba on the surface is ignored. This requires more clarification and the reveal of the details of the DFT models and their progressive optimisation.*

R2: Thank you for your valuable comments. According to R1 above, we have classified the positions of Ba species in TiO_2 surface and bulk. The structural feature of TiO_2 enables the coordination numbers of Ba species to be five in surface and six in bulk, which is obtained from the geometry optimization of DFT model. Within the bulk region, we focus primarily on the doping effect of Ba on the band structure and optical properties, as bulk TiO_2 dominates the photoexcitation process. In contrast, surface Ba species directly govern surface catalysis by acting as the genuine reaction centers, rather than exerting a long-range influence from bulk Ba dopants. Therefore, the bulk Ba species produce little effect on surface catalysis compared to surface Ba species. Similarly, the surface Ba species produce little effect on photoexcitation compared to bulk Ba species.

Q3: *In response to my comment 4, the authors accept my criticism that the model of Ba surface atoms on Ti(001) is not consistent with the realistic structure of TiO_2 obtained after MIL-125 calcination. This fact calls into question all mechanistic results from DFT.*

R3: We thank the reviewer for this insightful comment. Firstly, we make a mistake on describing the type of surface used in the calculation in the previous R4. "Firstly, **the anatase (0 0 1) facet** has been considered the most stable and representative surface for TiO_2 materials including nanoparticles". The "anatase (0 0 1) facet" should be corrected into "**anatase (1 0 1) facet**" since the stable anatase (1 0 1) facet have been used in all calculations. Besides, due to the high stability on the thermodynamics, the stable anatase (1 0 1) facet has been widely considered as the main exposed surface in many reported works (*Nat. Chem.*, 2025, 17, 890–896; *Nat. Mater.,* 2023, 22, 619–626; *Nat. Energy*, 2025, 10, 1095–1106) in which the morphology of TiO_2 nanomaterials is nanoparticle and anatase (1 0 1) facet is used in the simulation. Additionally, the reviewer raises concern that the sample morphology is not perfectly flat, making the direct use of the (1 0 1) facet as the exposed surface potentially unreasonable. Regarding this issue, it should be emphasized that for TiO_2 nanomaterials larger than 500 nm, the local structure at the ~2 nm scale (corresponding to our supercell model) can be reasonably regarded as a flat surface. This is analogous to perceiving the Earth's surface as flat despite

its global spherical curvature. Furthermore, calcination or other high-temperature treatment during the synthesis of TiO_2 effectively enhances its crystallinity and optimizes the surface structure by reducing defects. Especially, some other unstable surfaces, such as unstable (0 0 1) facet, is probably converted into the thermodynamically more stable TiO_2 (1 0 1) facet (*Surf. Sci.*, 2000, 460, 162–169; *Chem. Mater.*, 2011, 23, 3486–3494; *Chem. Eur. J.*, 2011, 17, 5949–5957). Based on the above considerations, the TiO_2 (1 0 1) facet was adopted as the representative surface model in our calculations.

Reviewer #5

Q1: *To R1(a): the authors show no evidence of the absence of Ba-Ba distances in the second coordination shell - neither in their response, nor in their revised MS or ESI. The sentence "However, such a feature is absent in the experimental EXAFS spectra" is thus not supported by the data or the presented analyses.*

R1: We thank the reviewer for this important comment. To address this concern, we have included synchrotron EXAFS data of BaO as a reference in the revised manuscript (**Fig. 2g**). In BaO, a pronounced Ba–O scattering contribution at 2.08 Å is clearly observed in the first coordination shell in R-space. However, the Ba–O scattering contribution in Ba:TiO_2 is observed at 2.18 Å, which is different from that in BaO. It should be noted that a weak peak at 2.93 Å is attributed to the scattering contribution of partial long Ba–O bonds according to the structural feature of surface Ba species and TiO_2 crystal (**left image**). Besides, the second-shell Ba–Ba scattering contribution of BaO is clearly observed at 3.32 Å, while the R-space spectra of Ba:TiO_2 show only a weaker feature assignable to Ba–Ti scattering at 3.45 Å. Moreover, from the supplementary STEM image of **R2**, it is evident that high-density Ba single atoms are uniformly dispersed within the TiO_2 matrix. This distribution pattern is clearly distinguishable from that of bulk BaO or BaO particles. Therefore, based on these combined experimental observations and fitting results, we have revised the statement in the manuscript to more precisely reflect that no detectable Ba–Ba scattering contribution is present.

Lines 181–185: *Notably, the peak at 2.93 Å can be attributed to the scattering signals of Ba–O bonds with different bond lengths. Meanwhile, the R-space spectra of Ba:TiO_2 exhibit only a weaker feature assignable to Ba–Ti scattering at 3.45 Å, while the second-shell Ba–Ba scattering contribution of BaO is clearly observed at 3.32 Å. The overall suppressed Ba–Ba coordination signal indicates that Ba species predominantly exist as atomically dispersed single atoms within the TiO_2 matrix.*

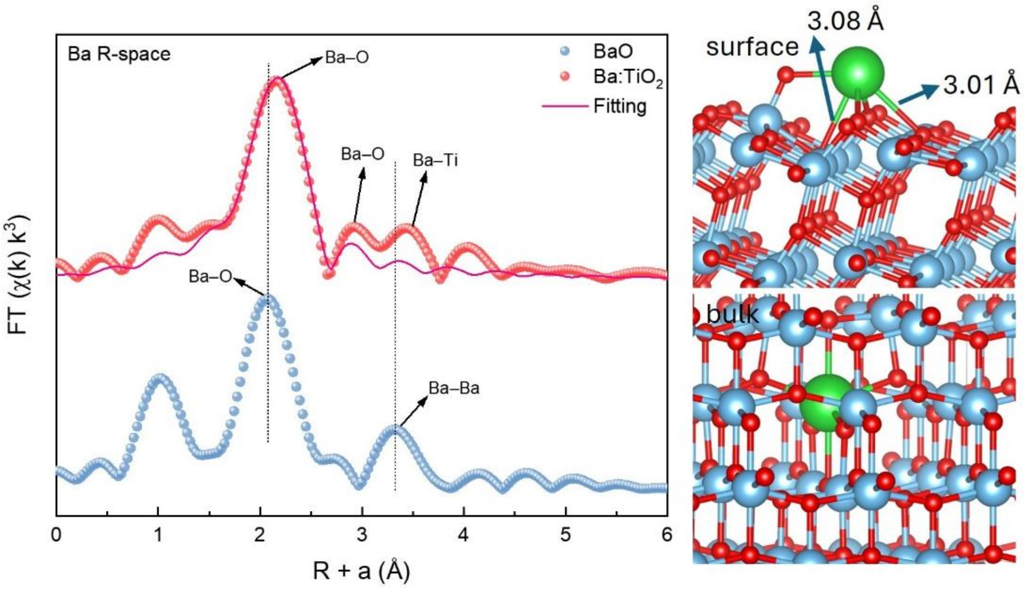

***Fig. 2g** Fourier transform of k^3-weighted EXAFS spectra at Ba K-edge of Ba:TiO_2 and BaO*

Q2: *Figure R7: Given the high atomic number of Ba vs Ti, I would expect the single atoms of Ba to be clearly visible in the TEM image. However, even if the authors highlight the "single atoms" with circles, the contrast differences are marginal, rendering these images unreliable as proof.*

R2: We thank the reviewer for this insightful comment. We agree that, in principle Ba atom ($Z = 56$) should exhibit stronger contrast than Ti atom ($Z = 22$) in HADDF-STEM imaging. We performed the STEM measurement with higher resolution and also provide a three-dimensional surface intensity mapping of the STEM images (***Supplementary* Fig. 12**). This analysis reveals that the localized intensity maxima is clearly distinguishable from the surrounding TiO_2 matrix. These isolated high-intensity features are consistent with the presence of atomically dispersed heavy elements. We note that the 3D intensity mapping is used here as a supplementary visualization tool to enhance contrast identification, rather than as standalone proof. Combined with the absence of Ba–Ba coordination in EXAFS, these observations collectively support the assignment of atomically dispersed Ba species.

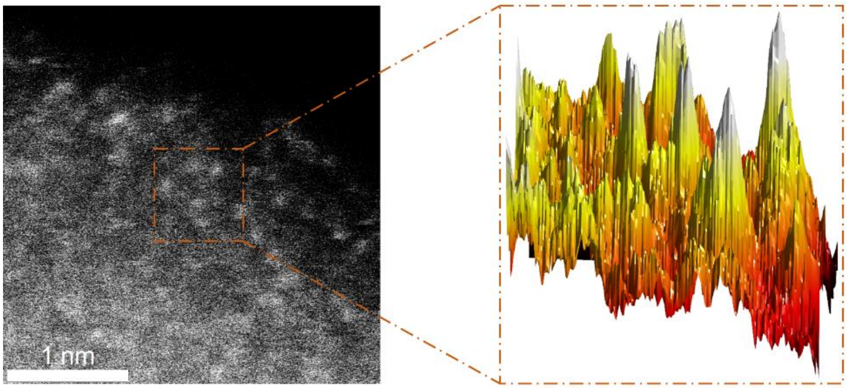

***Supplementary* Fig. 12** AC-STEM image of Ba:TiO_2, inset: 3D color map of the cyan-outline region, the protruding tip being Ba single atoms.
